# Pathogenic lineage of Perkinsea associated with mass mortality of frogs across the United States

**DOI:** 10.1038/s41598-017-10456-1

**Published:** 2017-08-31

**Authors:** Marcos Isidoro-Ayza, Jeffrey M. Lorch, Daniel A. Grear, Megan Winzeler, Daniel L. Calhoun, William J. Barichivich

**Affiliations:** 1 National Wildlife Health Center-U.S. Geological Survey, Madison, 53711 Wisconsin USA; 2 University of Wisconsin, School of Veterinary Medicine, Department of Pathobiological Sciences, Madison, 53706 Wisconsin USA; 3 South Atlantic Water Science Center-U.S. Geological Survey, Norcross, 30093 Georgia USA; 4 Wetland and Aquatic Research Center-U.S. Geological Survey, Gainesville, 32653 Florida USA

## Abstract

Emerging infectious diseases such as chytridiomycosis and ranavirus infections are important contributors to the worldwide decline of amphibian populations. We reviewed data on 247 anuran mortality events in 43 States of the United States from 1999–2015. Our findings suggest that a severe infectious disease of tadpoles caused by a protist belonging to the phylum Perkinsea might represent the third most common infectious disease of anurans after ranavirus infections and chytridiomycosis. Severe Perkinsea infections (SPI) were systemic and led to multiorganic failure and death. The SPI mortality events affected numerous anuran species and occurred over a broad geographic area, from boreal to subtropical habitats. Livers from all PCR-tested SPI-tadpoles (n = 19) were positive for the Novel Alveolate Group 01 (NAG01) of Perkinsea, while only 2.5% histologically normal tadpole livers tested positive (2/81), suggesting that subclinical infections are uncommon. Phylogenetic analysis demonstrated that SPI is associated with a phylogenetically distinct clade of NAG01 Perkinsea. These data suggest that this virulent Perkinsea clade is an important pathogen of frogs in the United States. Given its association with mortality events and tendency to be overlooked, the potential role of this emerging pathogen in amphibian declines on a broad geographic scale warrants further investigation.

## Introduction

Human-related activities are responsible for profound and rapid changes to natural environments that lead to habitat destruction, degradation, fragmentation, and the emergence of infectious diseases, all of which can mediate extinction of amphibian populations^1, 2^. From a disease standpoint, *Batrachochytrium dendrobatidis*, has significantly contributed to global amphibian declines and loss of biodiversity^3, 4^. Other diseases such as infections with ranaviruses are also becoming recognized causes of amphibian mortality worldwide and are linked to population declines in North America and Europe^5^.

Since 1999, infections of tadpoles by a protozoon with direct life cycle, belonging to the superphylum Alveolata, phylum Perkinsea have been reported on multiple continents^6–8^. Based on the sequence of the 18S SSU ribosomal RNA encoding gene (18S rRNA gene), Chambouvet *et al*.^7^ identified a very diverse monophyletic group of Perkinsea organisms referred to as Novel Alveolate Group 01 (NAG01) from freshwater and livers of tadpoles collected in North America, South America, Africa and Europe. The Perkinsea identified in tadpole livers from that study grouped into four phylogenetic clades within NAG01. Three of those clades were geographically widespread and caused cryptic infections with no signs of pathology to the host. The fourth clade was represented by a single location in Georgia, United States (US), where the infections by Perkinsea were associated with a mortality event^9^.

In the US, severe Perkinsea infections (SPI) have been reported in association with mass tadpole die-offs in Florida^10^, Georgia^9^, Minnesota, Mississippi, and New Hampshire^11^. Mortality rates as high as 95% have been reported during these outbreaks^11^. Affected tadpoles exhibited gross and histopathologic lesions in the liver, mesonephros, spleen, pancreas, gills, gastrointestinal tract, skeletal muscle, dermis and peritoneum^8–11^. However, the extents to which SPI is an important contributor to mortality over a broader geographic area and the range of host species susceptible to the infections have not been investigated. In addition, it is unknown whether the Perkinsea associated with these mortality events are genetically similar (i.e., the same species or strain) or whether multiple Perkinsea taxa are involved.

This study provides a description of the frequency, geographical, seasonal, and host distribution of SPI events from 1999 to 2015. These data provide evidence to suggest that SPI is a previously overlooked threat for anurans of North America. In addition, we demonstrate that a pathogenic Perkinsea clade (PPC) within the NAG01 group is responsible for all SPI-associated frog mortalities for which molecular characterization of the pathogen was conducted.

## Results

### Epidemiology of SPI

**Figure 1:**
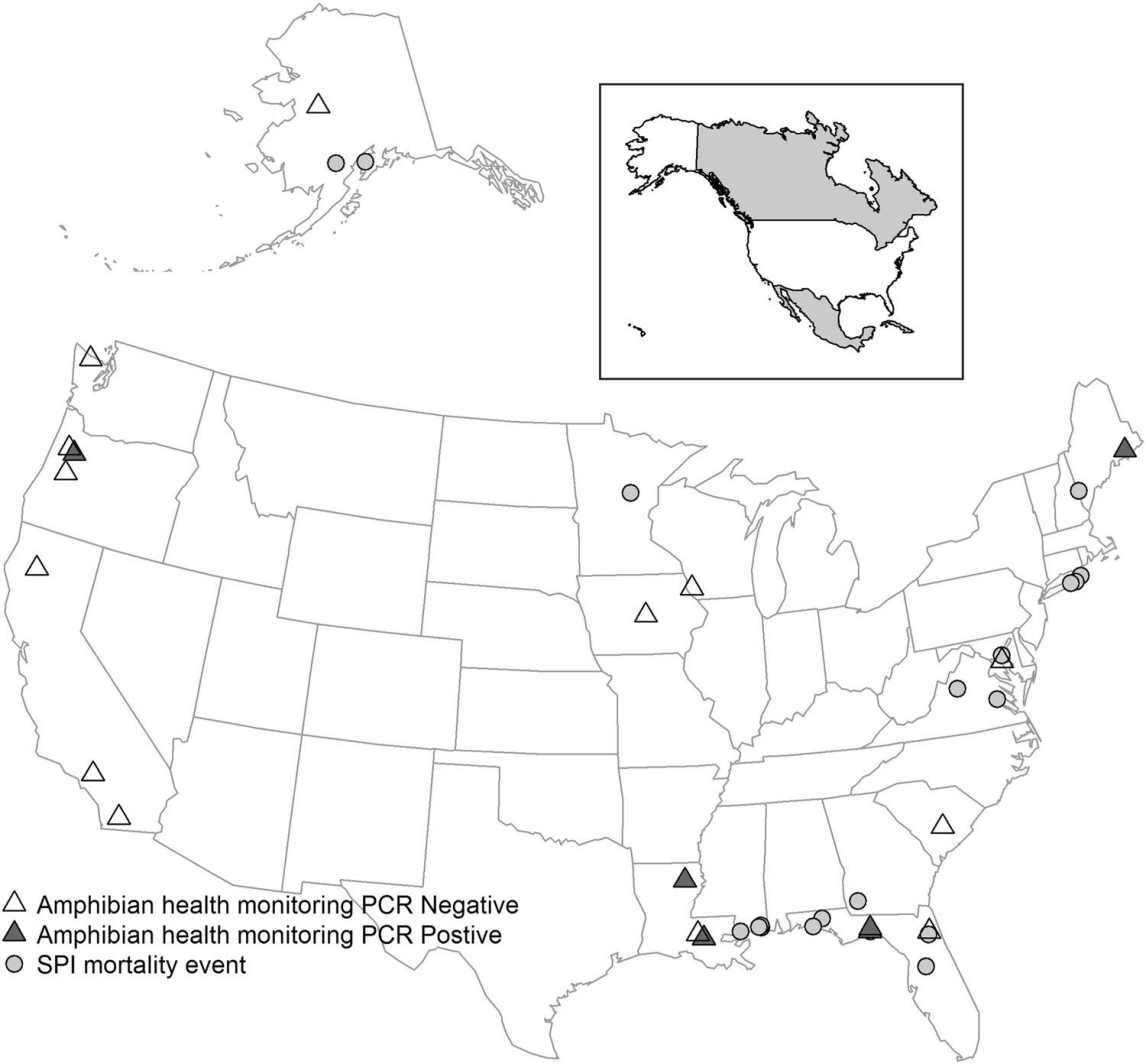
Geographic distribution of mortality events associated with severe Perkinsea infection (SPI) and detections of Novel Alveolate Group 01 (NAG01) Perkinsea by polymerase chain reaction amplification (PCR) in tadpoles collected from mortality events and health monitoring studies investigated by the U.S. Geological Survey-National Wildlife Health Center (NWHC), and the U.S. Geological Survey Amphibian Research and Monitoring Initiative (ARMI) in the United States, 1999–2015. Grey circles represent sites where SPI associated mortality events took place. Black triangles denote sites where NAG01 Perkinsea was detected in tadpoles collected during the course of amphibian health monitoring studies. Empty triangles represent sites from where apparently normal tadpoles were collected as part of amphibian health monitoring studies and screened for NAG01 Perkinsea with negative results. Map produced using R and the maps package v3.1.1^40^.

Of the 247 wild anuran mortality events we investigated in 43 States of the US from 1999 to 2015, 168 were associated with infectious diseases. Twenty-one of these mortality events, all involving tadpoles, were attributed to SPI. Estimated mortalities in these events were categorized as <100 tadpoles in 11 events (52%), 100–999 tadpoles in seven events (33%), and ≥1000 tadpoles in 3 events (14%). The geographic distribution of the events was broad, including 10 States within the US that spanned from Alaska to Florida (Fig. 1, Supplementary Table S1). Most SPI events occurred in States bordering the Atlantic Ocean and Gulf of Mexico (Fig. 1). However, SPI was also detected on the West Coast (Alaska and Oregon) and in the upper Midwest (Minnesota). Two events in Alaska in 2004 and 2005 represent the highest latitude where SPI has been detected. Repeated SPI events in consecutive or non-consecutive years were detected at three different sites. Nine out of the 11 events that occurred in boreal and temperate regions (82%) took place between June and September, and the remaining two events occurred in November. Conversely, nine of the ten SPI events investigated in States of the Southeastern US with subtropical climate (90%) occurred from December to May, with the remaining event occurring between the end of June and the beginning of July (Supplementary Table S1). Eleven different species of frogs belonging to the family Hylidae and Ranidae were affected, including the critically endangered dusky gopher frog (*Rana sevosa*) in its only remaining breeding locations (Supplementary Table S1). Mortality events were generally characterized by rapid onset. Sick tadpoles were noted as being bloated, unable to dive, and exhibited unusual behaviours such as swimming in circles or gaping. In addition to these 21 mortality events, SPI was also detected during the course of eight amphibian health monitoring studies in which mortality may have occurred but was not specifically tracked or estimated (Supplementary Table S1).

### Pathology of SPI

**Figure 2:**
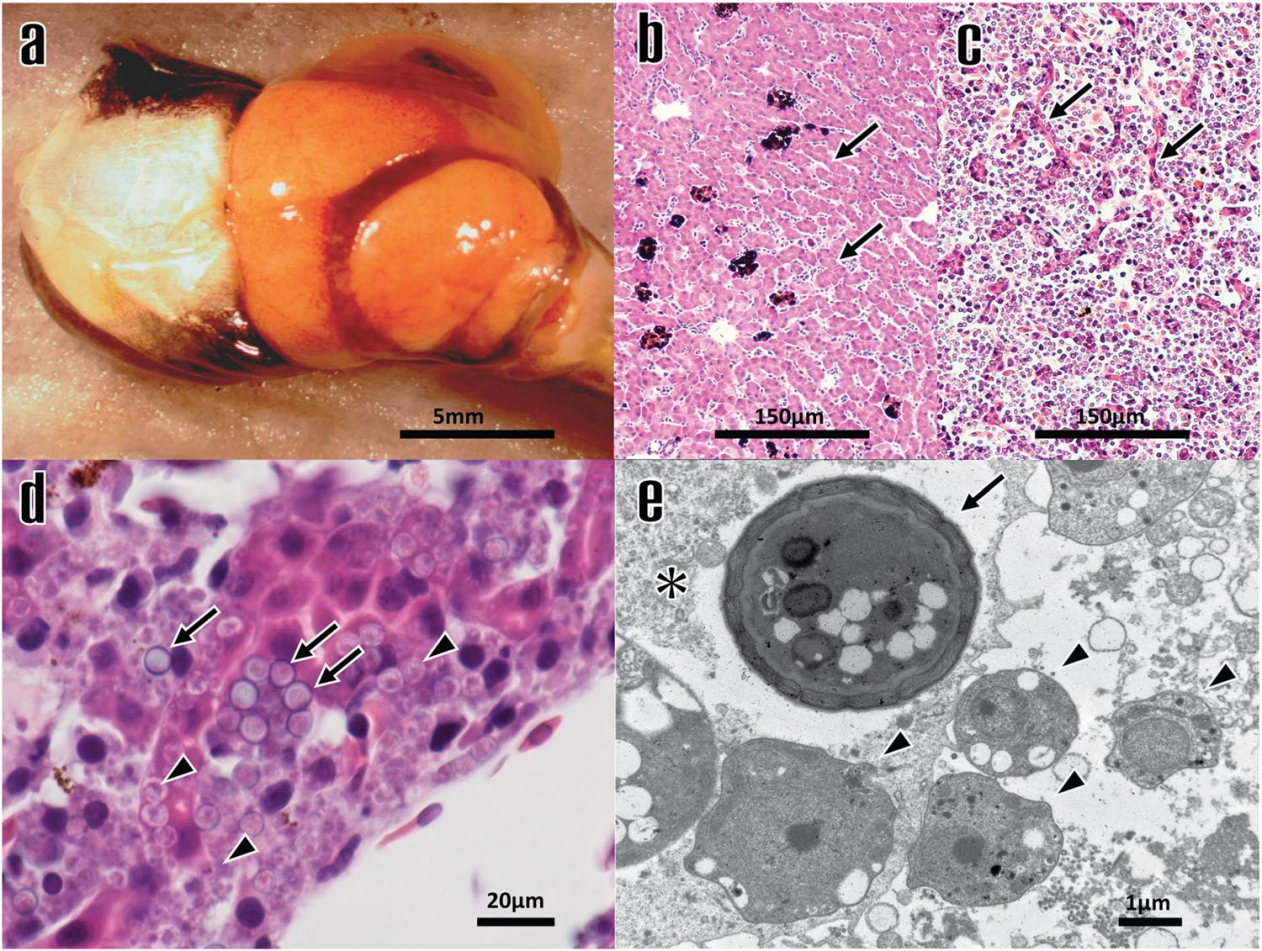
(**a**) Gross photograph of liver from Severe Perkinsea infection (SPI)-positive American bullfrog tadpole (*Rana catesbeiana*; NWHC #4824-453). Note that the liver is severely enlarged (hepatomegaly), approximately three times its normal size and exhibits pale-yellow discoloration. (**b**) Photomicrograph of liver from a non-infected Southern leopard frog tadpole (*Rana sphenocephala*; NWHC #18761-004), showing normal hepatic architecture. The arrows show normal hepatocytes forming hepatic cords. (**c**) Photomicrograph of liver from an SPI-positive *Rana catesbeiana* (NWHC #16407-009). Note that approximately 90% of the hepatic parenchyma is diffusely replaced by large numbers of Perkinsea-like protozoa. Arrows indicate few remaining, degenerating hepatic cords. (**d**) Photomicrograph of liver from SPI-positive *Rana sphenocephala* (NWHC #18967-001). Note that there are two distinct Perkinsea-like stages invading hepatocytes and causing degeneration, necrosis and disruption of hepatic cords; a spore-like stage, characterized by 4 to 6-µm diameter spherical structures with thick, deep basophilic wall, and granular pale basophilic cytoplasm (arrows); and a 1.8 to 3-µm diameter amoeboid, pale basophilic trophozoite-like stage (arrow heads). (**e**) Transmission electron photomicrograph of liver from an SPI-positive *Rana catesbeiana* (NWHC #16407-009). The cytoplasm of one hepatocyte (asterisk) is occupied by one Perkinsea-like spore (arrow) and two Perkinsea-like trophozoites (arrow head). In the extracellular space there are four trophozoite-like structures (arrow heads). Three of them are attached to the infected hepatocyte cell membrane.

Severe Perkinsea infections were diagnosed in a total of 225 tadpoles (182 tadpoles from the 21 mortality events and 43 tadpoles from the amphibian health monitoring studies referenced above). Diagnosis was based on gross and microscopic examination of affected organs in 209 tadpoles (93%). Gross examination of internal organs was used to diagnose the disease in 16 tadpoles (7%) for which post-mortem state was unsuitable for microscopic evaluation; these 16 tadpoles all originated from mortality events or health monitoring studies where the disease was also confirmed by histopathology in individuals with better post-mortem preservation state. Severe Perkinsea infection presented as an overt, severe and systemic disease and pathological changes were consistent with previous reports^9–11^. Tadpole life stage^12^ was determined and reported in 215 out of 225 SPI tadpoles (96%). Seventy-seven of them (36%) were hatchlings (Gosner stages 25–30) and 138 (64%) were larvae and metamorphs (Gosner stages 31–46). No adult frogs were diagnosed with SPI. In most cases, fat bodies (body fat stores) were present at the moment of death which suggested quick onset and acute course of disease. The most severely affected organ was the liver followed by mesonephros. Grossly, these organs often presented moderate to severe enlargement with pale-yellow discoloration (Fig. 2a). Histologically these gross changes corresponded with replacement of 50 to 90% of the pre-existent tissues by variable amounts of necrotic debris, fibrin and erythrocytes (haemorrhages), and massive numbers of Perkinsea-like organisms (Fig. 2c). Other commonly affected tissues were spleen, pancreas, gills, and digestive tract (stomach and intestine). In some cases skeletal muscle, superficial dermis, peritoneum, leptomeninges, choroid plexus, retina, and lungs (when present), were variably affected. Two distinct Perkinsea-like stages were identified by light microscopy and electron microscopy in the affected organs: a usually prevailing spore-like stage and a typically less abundant (but occasionally dominant) trophozoite-like stage that may have remained undescribed until now (Fig. 2d,e). However, additional work is necessary to confirm that these different structures represent various stages of the same Perkinsea organism. More detailed descriptions of the ultrastructural characteristics of the two Perkinsea-like stages are presented in the Supplementary Material, (Supplementary Fig. S1a, S1b).

### Ranavirus infections and chytridiomycosis screening in SPI mortality events

As part of the diagnostic process, screening for other relevant infectious agents of frogs was carried out to determine the relative importance of co-infections in SPI-related mortalities. In 13 out of 21 (62%) SPI mortality events, we screened a subset of tadpoles for ranavirus infection by polymerase chain reaction (PCR)^13^. Ranavirus was detected by PCR in only one out of the 13 SPI-related mortality events (in two out of the three tadpoles screened from that mortality event). One of these two tadpoles that was ranavirus-positive was also overwhelmingly infected by Perkinsea organisms, which obscured possible ranavirus-associated histological lesions; no histologic examination was performed on the second PCR-positive tadpole. In two out of the eight SPI events in which PCR screening for ranavirus was not carried out, systemic ranavirus infection was suspected based on histopathology in a subset of SPI-negative tadpoles (five tadpoles) collected during the outbreak. In addition, in four out of 21 SPI events there was a subset of tadpoles (seven in total) with presumptively concurrent oral chytridiomycosis (histological diagnosis without attempted molecular identification of *B. dendrobatidis*).

### NAG01 Perkinsea molecular screening of SPI tadpoles (clinical infections)

To confirm that the organisms observed microscopically were Perkinsea, livers from 19 affected tadpoles were screened for the presence of NAG01 Perkinsea using a PCR targeting the small subunit (SSU) ribosomal RNA gene^7^. Fourteen of these tadpoles were from 11 SPI mortality events and the remaining five were from four SPI-positive health monitoring studies (Supplementary Table S1). All 19 samples (100%) that were histologically positive for SPI were also PCR-positive for NAG01 Perkinsea. For ten SPI mortality events and four SPI positive health monitoring studies, samples could not be screened by PCR because frozen tissues were not available.

### NAG01 Perkinsea molecular screening of apparently normal tadpoles (subclinical infections)

In order to determine the prevalence of subclinical infections in the US, livers from 81 grossly and microscopically normal tadpoles from different States, years, species and life stage were also screened for NAG01 Perkinsea organisms (29 from health monitoring studies and 52 from mortality events) (Supplementary Table S1). Of the 81 apparently normal tadpoles screened, 38 were collected from sites with no known history of SPI, 14 were collected from sites with a known history of SPI but without observed mortality at the time of collection, and 29 were collected from SPI sites during an outbreak but were not infected by Perkinsea based on gross and histologic examination. Only two (2.5%) apparently normal tadpoles were PCR-positive for NAG01 Perkinsea organisms. These two positive tadpoles were collected as part of health monitoring studies from two wetlands where we had previously confirmed SPI mortality events. Specifically, one tadpole was collected from a site where, six years prior, SPI caused mortality in thousands of tadpoles; the other tadpole was collected from a site where, eight years prior, hundreds of tadpoles died as a consequence of an SPI outbreak. The remaining 79 apparently normal tadpoles were PCR negative, including those from sites with ongoing SPI outbreaks.

### Genetic characterization of Perkinsea associated with SPI

**Figure 3:**
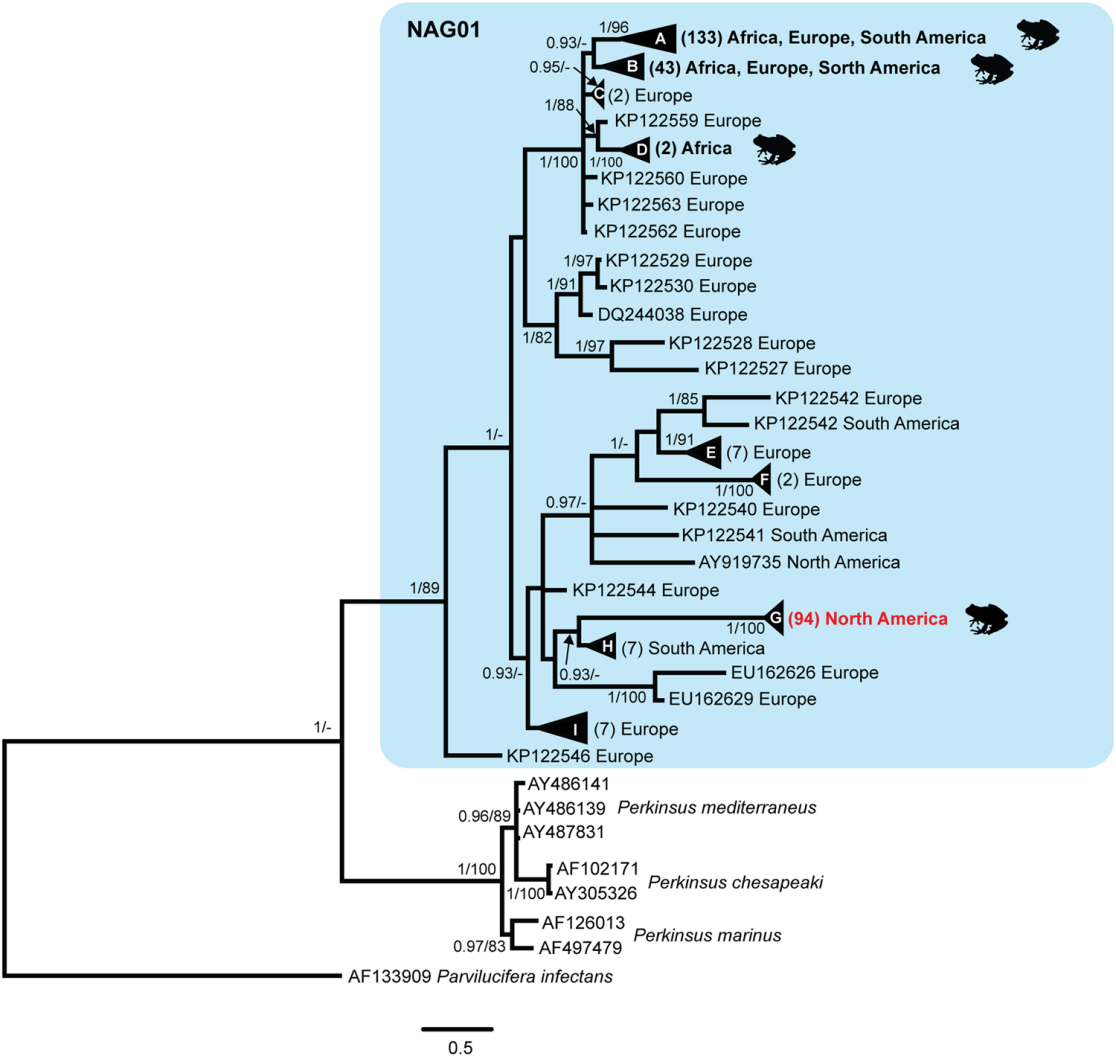
Phylogenetic tree of the NAG01 group of Perkinsea based on DNA sequences of a portion of the 18S SSU ribosomal RNA encoding gene including 755 characters. The tree from the Bayesian analysis is shown (the tree from the maximum likelihood analysis had a similar topology). Posterior probabilities (Bayesian)/bootstrap values (maximum likelihood) are shown at nodes when the support values were above 0.9 and 80, respectively. Sequences used to generate the tree are listed by GenBank number and the geographic location from which the sample containing the Perkinsea originated. Lettered triangles denote collapsed clades. The number of sequences represented in those collapsed clades is presented in parentheses; the samples residing in those clades can be found in Supplementary Table S3. Clades marked with a frog symbol contain Perkinsea representatives detected from the internal organs of tadpoles; all other Perkinsea within the NAG01 group were previously detected in environmental (i.e., freshwater) samples and are not known to be infectious agents of amphibians. All Perkinsea sequences derived from North American frogs with severe Perkinsea infections in this study resided within a unique clade (clade G; marked in red); the other clades of Perkinsea found in frogs have only been documented to cause cryptic infections.

A portion of the parasite’s 18S rRNA gene from all the PCR positive tadpoles, including the 19 SPI tadpoles (representing Perkinsea from 11 mortality events and four SPI-positive health monitoring studies) and the two PCR positive apparently normal tadpoles, was sequenced in both directions. All sequences generated (including cloned sequences) had 99.5% or higher identity with each other and with previously published Perkinsea sequences from an SPI outbreak in Georgia in 2006^9^. Phylogenetic analyses using maximum likelihood and Bayesian approaches on an alignment containing 755 characters confirmed that this pathogenic Perkinsea from North American frogs formed a strongly supported clade within the NAG01 that was distant from the other Perkinsea sequences previously published in GenBank (Fig. 3). Single nucleotide polymorphisms (SNPs) that were observed between the non-cloned PCR products often had ambiguous peaks at the SNP locations when the original chromatograms were examined. Sequences generated from the clones suggested that the SNPs in the non-cloned products may have represented intragenomic variation in the 18S rRNA gene as suggested by Chambouvet *et al*.^7^. It is also possible that some frogs may have been co-infected with multiple strains of the Perkinsea that differed slightly in the sequence of the 18S rRNA gene.

## Discussion

Severe Perkinsea infection (21 events) represents the third most common infectious disease associated with the wild anuran mortalities that we investigated from 1999 to 2015 after ranavirus infections (92 events) and chytridiomycosis (50 events) (Supplementary Table S2). The striking pathological findings observed with SPI establish a clear link between this disease and mortality in wild North American tadpoles. NAG01 Perkinsea PCR was positive for all tested SPI tadpoles and negative for 97.5% of the apparently normal tadpoles from a diverse geographic, temporal and life-stage range tested for this study. These results suggest that NAG01 Perkinsea organisms are strongly associated with severe disease in North America and do not likely persist as subclinical infections. Instead, the pathogen may persist in the environment^8, 14^, in paratenic hosts^8^, or infect only a small proportion of tadpoles in some years.

In contrast to the diversity of Perkinsea identified in asymptomatic frogs from Africa, Europe, and South America^7^, our findings suggest that a single type of Perkinsea is likely responsible for the severe infections observed across the US. We tentatively refer to this virulent lineage as the “pathogenic Perkinsea clade” (PPC) since infections with these strains were associated with overt disease and were obtained from tadpoles during SPI mortality events, or rarely, from apparently normal tadpoles residing in wetlands with a history of SPI mortality events. Pathogenic Perkinsea clade appears to be genetically divergent from described species within the same class for which genetic data were available for comparison. However, taxonomy for this group can be challenging and failed attempts to isolate and grow PPC in culture have hampered the ability to more fully characterize the organism. Thus, an official description, naming, and precise taxonomic placement of PPC were beyond the scope of this project. That a single species or lineage is responsible for SPI allows future efforts to focus on one particular taxon. In this respect, the finding is similar to the discovery of a global panzootic lineage of *B. dendrobatidis* being responsible for epizootic events of chytridiomycosis^15^. However, further genetic analyses are needed to better elucidate the evolutionary history and virulence of PPC.

Based on current knowledge of host-parasite ecology, SPI fulfills several criteria consistent with a disease capable of causing local population declines and extinctions^16–19^. First, PPC has the capacity to survive at a low threshold host population density. The spores are highly persistent in harsh environmental conditions and remain viable during desiccation of wetlands^8^. Second, PPC can infect a wide range of frog hosts. We confirmed SPI in 11 anuran species in the US (Supplementary Table S1). These species include representatives from the families Hylidae and Ranidae, two diverse families with a nearly worldwide distribution. Although most events were reported affecting common anuran species, two outbreaks of SPI in critically endangered dusky gopher frogs highlight the risk this disease can pose for threatened species^20^. In the case of these two outbreaks, radical management measures had to be adopted to prevent recruitment failure and preserve the species^8, 14^. Furthermore, the recognized number of susceptible host species and geographic range of SPI will likely broaden as this disease gains more attention. Third, SPI outbreaks exhibit high mortality rates along with local recurrence. For the events that we reviewed, SPI caused mass mortalities involving an estimated hundreds or thousands of animals in 43% (6/14) of the events in which it was the sole aetiological agent detected (Supplementary Table S1). The magnitude of these events is similar to mortality described in the SPI die-off previously reported in Georgia^9^. Although SPI has not been reported outside of the US, the presence of events in Alaska and other northern States indicates that this disease is likely distributed throughout much of the Nearctic region. Therefore, efforts should be made to screen for the pathogen elsewhere and determine whether introduction of PPC to different parts of the world could have devastating impacts on naïve amphibian populations in those areas.

Infections caused by Perkinsea apparently affect only tadpoles. The lack of SPI in adult frogs in this study and in previous reports of SPI mortality events^9, 10^ suggests: that the immune system either eliminates the pathogen as the animal matures due to a more competent immune response to fend off the infection^21^; or that most infected individuals die prior to completing metamorphosis. A single report describing Perkinsea-like spores in a granulomatous lesion in the leg of an adult frog^22^ supports the hypothesis that Perkinsea infections are rare and localized in post-metamorphic stages.

The SPI outbreaks occurred mostly from summer to early autumn in boreal and temperate regions, and from late winter to early spring (and occasionally during the summer) in States with a subtropical climate. This seasonality coincides with temperature and rainfall dependent breeding patterns of most amphibian species in the different regions^23^. This might be the result of phenological synchrony between parasite and host by which high abundance of the infective form of the parasite concurs with the most susceptible stage of the host (tadpoles) in shared aquatic environments. Infection dynamics of Perkinsea have been best-studied in *Perkinsus marinus*, which causes mortality (dermo disease) in eastern oysters (*Crassostrea virginica*), leading to large economic losses for this fishery industry along the East Coast of North America, and as PPC, also has a direct life cycle^24–26^. Abundance of *P. marinus* in the water environment and the infection rate of oysters increase with temperature^27^. Likewise, PPC abundance might increase above a certain water temperature range coinciding with the tadpole season. Nevertheless, since our phylogenetic analysis suggests a significant distance in the evolutionary history of PPC and *P. marinus*, it is difficult to extrapolate disease dynamics between these two distantly related Perkinsea. Therefore, specific studies on SPI dynamics focused on understanding environmental conditions (including those influenced by anthropogenic activities) that trigger outbreaks are needed to better understand the ecology of this disease and hence, to make management decisions to minimize associated tadpole mortalities.

In most events in which SPI was diagnosed, it was considered the only aetiologic agent responsible for mortality (14/21; 67%). However, ranavirus was detected by PCR in tadpoles from one SPI event; in two additional SPI events, ranavirus infection was suspected based on histopathology in a subset of SPI-negative tadpoles collected during the outbreak. Severe Perkinsea infections and ranaviruses that affect anurans in North America^5^ preferentially target tadpole stages and might cause synergistic deleterious effects in some frog populations. For example, concurrent outbreaks of SPI and ranavirus could decimate an entire age class within a wetland, and alternating outbreaks could have more chronic population effects due to decreased recruitment. Furthermore, in four SPI events, histopathology revealed that a subset of tadpoles had concurrent chytridiomycosis of the mouthparts. Although the role of oral chytridiomycosis in mortality events of tadpoles of some anuran species has been questioned^28^, *B. dendrobatidis* may cause severe disease and death of adult frogs within the same population. Thus, the additive impacts of all three diseases on a population could stretch across multiple life stages.

Amphibian, particularly tadpole, mortality events are easily overlooked due to rapid scavenging and decomposition of carcasses^29–31^. In addition, limited laboratory testing that often focuses solely on PCR-based assays to common disease agents such as *B. dendrobatidis* and ranaviruses may result in PPC remaining undetected in some regions of the United States and abroad. For these reasons, the frequency, magnitude, and extent of SPI events may be greatly underestimated.

Long term studies were necessary to demonstrate that chytridiomycosis and ranavirus infections were emerging diseases with catastrophic effects on global amphibian populations. This study indicates that a third important pathogen – PPC – could also be a significant contributor to amphibian declines in certain areas, and more work is needed to understand its impacts on a global scale. Until this is achieved, increased screening for PPC and development of biosecurity protocols should be considered to prevent potential spread of this deadly disease.

## Materials and Methods

### Anuran mortality event investigation

The data for this study were compiled through investigation of wildlife mortality events by the U.S. Geological Survey – National Wildlife Health Center (NWHC) and the U.S. Geological Survey - Amphibian Research and Monitoring Initiative (ARMI) that involved species from the order Anura from 1999 to 2015. Events that received diagnostic and epidemiological investigation at the NWHC are those considered out of the normal range of observed mortality. The “normal amount” of mortality is rarely defined for amphibian species; hence the events were investigated if more than five individuals were observed dead at a single site within a short period of time (subjectively defined by the observer). Perkinsea infections in frogs detected as part of health monitoring studies were also included in this project. Health monitoring studies consisted of tadpoles collected for disease screening in the absence of a documented mortality event.

Epidemiological information was compiled from each anuran sample, including the detection type (mortality event or health monitoring study), location (State and county), anuran species reported in the event, life-stage of species reported in the event (tadpoles or adults), tadpole life-stage of specimen submitted for examination^12^, date of first observation of the event, estimate of the size of the event (based on total number of moribund and dead tadpoles encountered), and aetiology of the event. Anuran species and life-stage identification were confirmed at arrival by an NWHC expert herpetologist and pathologist based on external morphological features^12, 32, 33^. Event information was summarized from reports submitted to the NWHC by field biologists and from diagnostic reports generated by NWHC staff pathologists and laboratory diagnosticians. As such, event summaries had variable detail. We reported event date as the month and year of the first observation and collection of specimen. Event aetiologies were those considered to significantly contribute to mortality or morbidity based on expert epidemiological and diagnostic interpretation of pathological findings, bacteriology, parasitology and virology results (i.e., pathogens found in low abundance or without evidence of significant pathological impact to the host were not reported). We also compared the frequency of Perkinsea aetiology relative to all other anuran mortality event aetiologies investigated by the NWHC. Anuran mortality events were identified from the NWHC epidemiology records as any mortality event where a species in the order Anura was reported, and the event was investigated by the NWHC. We categorized event aetiologies into ranavirus infections, infections with non-ranavirus viruses, chytridiomycosis, non-chytrid fungal infections, SPI, infections with other parasites (including Protista and Animalia organisms), bacterial infections, and non-infectious sources (undetermined cause, predation, toxicity). We further classified events as either affecting tadpole life-stages (Gosner stage 20–46) or affecting only adults. Mortality of egg masses and embryos (Gosner stage 0–20) are not routinely investigated by the NWHC and therefore were not included in this study.

### Pathological investigation of SPI

SPI confirmation was based on the observation of characteristic gross and microscopic lesions and the morphological identification of Perkinsea organisms within the lesions. Necropsies and gross evaluation of carcasses were carried out under a dissecting microscope. Microscopic diagnosis of SPI was determined in most tadpoles (n = 174) by histologic examination of all major organs including brain, eyes, gastrointestinal tract, gills, heart, liver, lungs (when present), mesonephros, pancreas, skeletal muscle, skin, and spleen. Touch print cytology from liver sections was occasionally used (22 tadpoles from two SPI events and 13 tadpoles from four health monitoring studies) for identification of Perkinsea spores in poorly preserved tadpole carcasses with gross lesions suggestive of SPI. In seven frogs from three SPI events and nine frogs from two health monitoring studies in which SPI had been microscopically confirmed in other better preserved tadpole carcasses, the diagnosis of SPI was based on characteristic gross findings (severe hepatomegaly with yellow discoloration of the liver).

For histopathology, samples were fixed in 10% formaldehyde for at least 48 h. When the animals were late-stage tadpoles and had partially or completely ossified skeletons, the specimens were decalcified overnight in formic acid-sodium citrate mixture. Fixed samples were dehydrated with a graded ethanol series, embedded in paraffin and sectioned at a thickness of 4 μm with a rotary microtome. Sections were then stained using Mayer’s haematoxylin and eosin (H&E) method. For electron microscopy, a fragment of liver from a tadpole diagnosed with SPI was preserved in paraformaldehyde-glutaraldehyde solution (Karnovsky’s Fixative). Tissues were subsequently dehydrated in a graded ethanol series, infiltrated with epoxy propylene oxide, and embedded in epoxy resin. The epoxy block was then sectioned with an ultra-microtome at a thickness of 1 μm (semi-thin sections). Semi-thin sections were then stained with uranyl acetate followed by lead citrate, and examined with transmission electron microscope equipped with a digital photomicrograph (Hamamatsu ORCA HR Camera).

### NAG01 Perkinsea molecular screening

Tadpole liver tissue that had been stored at −80 °C was screened for the presence of Perkinsea within the NAG01 clade. DNA was extracted from the liver samples using the Gentra®Puregene® Tissue Kit (Qiagen Inc., Valencia, California, USA) according to the manufacturer’s instructions. To amplify DNA of the 18S rRNA gene of Perkinsea-like organisms within NAG01, primers 86F-B, 300F-B, 1294 R, and 1282 R were used^7^. Polymerase chain reaction was carried out using GoTaq® Flexi DNA polymerase (Promega Corporation, Madison, Wisconsin, USA). Each 25-µl reaction contained 13.375 µl nuclease-free water, 5 µl buffer, 2 µl dNTPs (2.5 mM each), 1.5 µl MgCl_2_ (25 mM), 1.25 µl of each primer (20 µM), 0.125 µl enzyme, and 0.5 µl undiluted or 10-fold diluted DNA template. Cycling conditions were: 95 °C for 2 min; 40 cycles of 95 °C for 30 sec, 55 °C for 30 sec, 72 °C for 2 min; and a final extension of 72 °C for 10 min. Each sample was run in four separate reactions (one for each possible primer pairing). Presence of potential Perkinsea-like DNA was assessed by the generation of an approximately 1-kb amplicon as viewed on a 1% agarose gel.

To ensure that samples contained adequate DNA for detection of Perkinsea, an approximately 2-kb portion of mitochondrial 12S-16S ribosomal DNA of the anuran host was amplified for all samples using primers 12L1 and 16H1^34^. Reaction chemistry was as described above, except that 1–5 µl of template was added per 50 µl reaction and the cycling conditions were: 95 °C for 5 min; 40–45 cycles of 95 °C for 1 min, 55–70 °C for 1 min, 72 °C for 2.5 min; and final extension of 72 °C for 10 min. If host DNA failed to amplify, the sample was excluded from further molecular analysis.

### Perkinsea DNA sequencing and phylogenetic analyses

PCR product was purified, when necessary, by using the QIAquick gel extraction kit (Qiagen Inc., Valencia, California, US) and subjected to double stranded DNA sequencing with the same primers used for amplification. In some instances, the amount of PCR product was insufficient for sequencing and nested PCR was used to generate more product. The same reaction and cycling conditions were used as described above using 0.5 µl of initial PCR product as template and primers that were internal to those used in the first round of amplification.

To determine if tadpoles might be co-infected with multiple types of Perkinsea organisms, a subset of nine samples (each representing a different mortality event) were amplified using a proofreading DNA polymerase and cloned to sequence individual amplicons. Each PCR reaction included 0.3 µl Pfx50TM DNA Polymerase (Invitrogen, Carlsbad, California, USA), 2.5 µl 10X Pfx50TM PCR mix, 0.375 µl each primer, 19.45 µl nuclease-free water, and 1 µl of undiluted, 1:10 diluted, or 1:100 diluted template (i.e., original DNA extracted from liver samples). Cycling conditions were as described above, except that the number of cycles was increased to 45 and temperatures were increased to 60 °C for the annealing and reduced to 68 °C for the extension steps. The resulting PCR product was cloned using the Zero Blunt® TOPO® PCR Cloning Kit (Invitrogen, Carlsbad, California, US). Eight clones containing an insert of the correct size were sequenced in both directions using the M13 primers. All Perkinsea sequences that were newly generated in this study were deposited in GenBank (Supplementary Table S3). Phylogenetic analyses were conducted using sequences for NAG01 Perkinsea available in GenBank and the novel sequences generated during this study. Sequences from both PCR product that was directly sequenced and that which was cloned prior to sequencing were included. Reference sequences for three *Perkinsus* species were also included, and a reference sequence for *Parvilucifera infectans* was used to root the tree. All sequences used in the analyses are listed in Supplementary Table S3. An alignment of the sequence data was generated using MUSCLE in the program MEGA version 6.0^35^ and all gaps were deleted to generate the final alignment of 755 characters. Maximum likelihood and Bayesian methods were performed using RAxML-HPC2^36^ and MrBayes version 3.2.6^37^, respectively, through the CIPRES Science Gateway^38^. For the maximum likelihood analysis, a general time reversible model with gamma distribution was used and 1000 bootstrap iterations were performed. For the Bayesian analysis, a Kimura 2 parameter model with gamma distribution was used (the best model for the alignment according to MEGA), and the number of generations was set to 5,000,000. All other parameters not specified above were left as default.

### Host species genetic identification

To confirm the morphology-based species identification of tadpoles, a subset (one representative of each host species from each site) of PCR amplicons representing host mitochondrial DNA (12S-16S ribosomal DNA) were sequenced. Double-stranded sequencing was conducted as described above using primers 12L1, 16H1, 12Sm, 16Sc, 16Sh, and 16Sa^34, 39^. In some instances, sequences could not be interpreted due to presence of multiple overlaid peaks. This was thought to be the result of primers binding to multiple locations on the amplicon. When this occurred, smaller products were generated from the original sample by using internal sequencing primers for initial amplification (i.e., primer pairs 12L1 and 16Sh, 12L1 and 16Sa, 12Sm and 16Sa, 12Sm and 16H1, and 16Sc and 16H1) and modified cycling conditions (95 °C for 5 min; 40 cycles of 95 °C for 1 min, 55–60 °C for 1 min, 72 °C for 2 min; final extension 72 °C for 10 min); then the fragments were sequenced with those same primers. All sequences generated from anurans in this study have been deposited in GenBank (Supplementary Table S4). Results of host identification based on DNA sequencing are presented in Supplementary Table S1. The currently-recognized host range of severe Perkinsea infections (SPI) in frogs is illustrated as a phylogenetic tree in Supplementary Figure S2.

Phylogenetic analyses were conducted to help assess the species to which anurans examined in this study belonged. A sampling of 40 species that occur within the geographic region covered by this project were included in the analysis using reference sequences available in GenBank (Supplementary Table S4). Sequence alignment was performed using MEGA as described above with the final alignment consisting of 1,718 characters. A general time reversible model with gamma distribution was used for both the maximum likelihood and Bayesian analyses, which were otherwise run as detailed for the Perkinsea analysis. Anurans from this study were assigned to a given species if they grouped in a clade with a reference sequence of that species with a posterior probability ≥0.95 or a bootstrap value ≥85. Scientific names used to designate true frog species are those suggested by Yuan *et al*.^39^.

### Ranavirus molecular screening

Mesonephros, liver or pooled internal organs of fresh chilled or frozen tadpoles were collected in viral transport medium and tested for the presence of ranaviruses by viral isolation in a fathead minnow fish cell line followed by frog virus-3 major capsid protein PCR as previously described^13^.

### Institutional Animal Care and Use Committee (IACUC) protocol

All samples used for this study were from tissue archives and originated from wildlife disease investigations conducted on amphibian carcasses from 1999 to 2015. Euthanasia of frogs included in this study was covered under IACUC protocol number EP080707.

### Data availability statement

All data generated or analysed during this study are included in this published article (and its Supplementary Information files).

## Electronic supplementary material

Supplementary Information
