# Supplementary material for: Pathogenic lineage of Perkinsea associated with mass mortality of frogs across the United States
Source: Sci Rep. 2017 Aug 31;7:10288. doi: 10.1038/s41598-017-10456-1 (PMC5579288; doi:10.1038/s41598-017-10456-1)
Supplement: Supplementary file 1 — Supplementary Information [file 41598_2017_10456_MOESM1_ESM.doc]

**Supplementary Information: Pathogenic lineage of Perkinsea causes mass mortality of frogs across the United States**

Marcos Isidoro-Ayza^1, 2, *^, Jeffrey M Lorch^1^, Daniel A Grear^1^, Megan Winzeler^1^, Daniel L Calhoun^3^, William Barichivich^4^

^1^ National Wildlife Health Center-U.S. Geological Survey, Madison, Wisconsin 53711, USA

^2^ University of Wisconsin, School of Veterinary Medicine, Department of Pathobiological Sciences, Madison, Wisconsin 53706, USA

^3^South Atlantic Water Science Center-U.S. Geological Survey, Norcross, Georgia 30093, USA

^4^Wetland and Aquatic Research Center-U.S. Geological Survey, Gainesville, Florida 32653, USA

[^*^marcos.isidoro.ayza@gmail.com](mailto:*marcos.isidoro.ayza@gmail.com)

**Supplementary Tables:**

| **Table S1:** Specimen data for carcasses examined via pathology for severe Perkinsea infection (SPI) and screened by PCR for Perkinsea Novel Alveolate Group-1 (NAG1) by the U.S. Geological Survey-National Wildlife Health Center (NWHC), and the U.S. Geological Survey Amphibian Research and Monitoring Initiative (ARMI).  Abbreviations: NA, non-available or non-applicable; Und, specimens for which no cause of death was determined after postmortem investigation (undiagnosed); NEG, negative result; POS, positive result.  ^a^Identification number for SPI associated mortality events (ME) or SPI positive anuran health monitoring studies (AHM) investigated in this study; ^b^NWHC epizootic database identification number; ^c^NWHC carcass identification number; ^d^Estimated number of tadpoles dead based on field observations; ^e^Carcass collection month; ^f^Anuran species identification confirmation by PCR. | | | | | | | | | | | | | | | | |
| --- | --- | --- | --- | --- | --- | --- | --- | --- | --- | --- | --- | --- | --- | --- | --- | --- |
| **ME/AHM #^a^** | **Epizoo Event #^b^** | **Carcass #^c^** | **Estimated field mortality^d^** | **State** | **Year** | **Month^e^** | **County or Parrish** | **Species** | **Gosner stage** | **Perkinsea NAG1 PCR** | **Anuran PCR^f^** | **SPI (histopathology based)** | **SPI (gross exam & cytology based)** | **SPI (gross exam based)** | **Primary diagnosis** | **Relevant co-infections** |
| 1 | 2004-074 | 19157-003 | 3 | AK | 2004 | Jul | Bethel | *Rana sylvatica* | 37 | NA | NO | YES | NO | NO | SPI | NA |
| 2 | 2004-073 | 19156-004 | 25 | AK | 2004 | Jul | Kenai Peninsula | *R. sylvatica* | 41 | NEG | YES | NA | NA | NA | Und | NA |
| 2 | 2004-073 | 19215-008 | 25 | AK | 2004 | Jul | Kenai Peninsula | *R. sylvatica* | 43 | NA | NO | YES | NO | NO | SPI | NA |
| 2 | 2004-073 | 19215-006 | 25 | AK | 2004 | Jul | Kenai Peninsula | *R. sylvatica* | 40 | NA | NO | YES | NO | NO | SPI | NA |
| 2 | 2004-073 | 19215-005 | 25 | AK | 2004 | Jul | Kenai Peninsula | *R. sylvatica* | 45 | NA | NO | YES | NO | NO | SPI | NA |
| 2 | 2004-073 | 19215-002 | 25 | AK | 2004 | Jul | Kenai Peninsula | *R. sylvatica* | 36 | POS | YES | YES | NO | NO | SPI | NA |
| 2 | 2004-073 | 19156-016 | 25 | AK | 2004 | Jul | Kenai Peninsula | *R. sylvatica* | 42 | NA | NO | YES | NO | NO | SPI | NA |
| 2 | 2004-073 | 19156-013 | 25 | AK | 2004 | Jul | Kenai Peninsula | *R. sylvatica* | 42 | NA | NO | YES | NO | NO | SPI | NA |
| 2 | 2004-073 | 19156-011 | 25 | AK | 2004 | Jul | Kenai Peninsula | *R. sylvatica* | 43 | NA | NO | YES | NO | NO | SPI | NA |
| 2 | 2004-073 | 19156-006 | 25 | AK | 2004 | Jul | Kenai Peninsula | *R. sylvatica* | 45 | NA | NO | YES | NO | NO | SPI | NA |
| 2 | 2004-073 | 19156-005 | 25 | AK | 2004 | Jul | Kenai Peninsula | *R. sylvatica* | 45 | NA | NO | YES | NO | NO | SPI | NA |
| 2 | 2004-073 | 19156-003 | 25 | AK | 2004 | Jul | Kenai Peninsula | *R. sylvatica* | 41 | NA | NO | YES | NO | NO | SPI | NA |
| 2 | 2004-073 | 19156-001 | 25 | AK | 2004 | Jul | Kenai Peninsula | *R. sylvatica* | 40 | POS | YES | YES | NO | NO | SPI | NA |
| 3 | 2005-050 | 19456-002 | 20 | AK | 2005 | Jun | Kenai Peninsula | *R. sylvatica* | 30 | NA | NO | YES | NO | NO | SPI | NA |
| 3 | 2005-050 | 19456-001 | 20 | AK | 2005 | Jun | Kenai Peninsula | *R. sylvatica* | 31 | NA | NO | YES | NO | NO | SPI | NA |
| 4 | 2002-149 | 18487-009 | 200 | FL | 2002 | Dec | Wakulla | *Rana sphenocephala* | 29 | NEG | YES | NA | NA | NA | Und | NA |
| 4 | 2002-149 | 18487-008 | 200 | FL | 2002 | Dec | Wakulla | *R. sphenocephala* | 26 | NEG | YES | NA | NA | NA | Und | NA |
| 4 | 2002-149 | 18487-007 | 200 | FL | 2002 | Dec | Wakulla | *R. sphenocephala* | 28 | NEG | YES | NA | NA | NA | Und | NA |
| 4 | 2002-149 | 18487-023 | 200 | FL | 2002 | Dec | Wakulla | *R. sphenocephala* | 25 | NA | NO | YES | NO | NO | SPI | NA |
| 4 | 2002-149 | 18487-022 | 200 | FL | 2002 | Dec | Wakulla | *R. sphenocephala* | 26 | NA | NO | YES | NO | NO | SPI | NA |
| 4 | 2002-149 | 18487-021 | 200 | FL | 2002 | Dec | Wakulla | *R. sphenocephala* | 26 | NA | NO | YES | NO | NO | SPI | NA |
| 4 | 2002-149 | 18487-020 | 200 | FL | 2002 | Dec | Wakulla | *R. sphenocephala* | 26 | NA | NO | YES | NO | NO | SPI | NA |
| 4 | 2002-149 | 18487-019 | 200 | FL | 2002 | Dec | Wakulla | *R. sphenocephala* | 25 | NA | NO | YES | NO | NO | SPI | NA |
| 4 | 2002-149 | 18487-017 | 200 | FL | 2002 | Dec | Wakulla | *R. sphenocephala* | 26 | NA | NO | YES | NO | NO | SPI | NA |
| 4 | 2002-149 | 18487-016 | 200 | FL | 2002 | Dec | Wakulla | *R. sphenocephala* | 26 | NA | NO | YES | NO | NO | SPI | NA |
| 4 | 2002-149 | 18487-015 | 200 | FL | 2002 | Dec | Wakulla | *R. sphenocephala* | 26 | NA | NO | YES | NO | NO | SPI | NA |
| 4 | 2002-149 | 18487-012 | 200 | FL | 2002 | Dec | Wakulla | *R. sphenocephala* | 25 | NA | NO | YES | NO | NO | SPI | NA |
| 4 | 2002-149 | 18487-011 | 200 | FL | 2002 | Dec | Wakulla | *R. sphenocephala* | 26 | NA | NO | YES | NO | NO | SPI | NA |
| 4 | 2002-149 | 18487-010 | 200 | FL | 2002 | Dec | Wakulla | *R. sphenocephala* | 26 | NA | NO | YES | NO | NO | SPI | NA |
| 4 | NA | 4864-012 | 200 | FL | 2003 | Feb | Wakulla | *R. sphenocephala* | 27 | NA | NO | YES | NO | NO | SPI | NA |
| 4 | NA | 4864-009 | 200 | FL | 2003 | Feb | Wakulla | *R. sphenocephala* | NA | NA | NO | YES | NO | NO | SPI | NA |
| 4 | NA | 4864-008 | 200 | FL | 2003 | Feb | Wakulla | *R. sphenocephala* | NA | NA | NO | YES | NO | NO | SPI | NA |
| 4 | NA | 4864-003 | 200 | FL | 2003 | Feb | Wakulla | *R. sphenocephala* | 26 | POS | YES | YES | NO | NO | SPI | NA |
| 5 | NA | 4864-072 | NA | FL | 2003 | May | Wakulla | *Acris gryllus* | 31 | NA | NO | NO | YES | NO | SPI | NA |
| 5 | NA | 4864-067 | NA | FL | 2003 | May | Wakulla | *R. sphenocephala* | 25 | NA | NO | YES | NO | NO | SPI | NA |
| 6 | 2005-016 | 19369-034 | 1000 | FL | 2005 | Mar | Hernando | *Rana catesbeiana* | 40 | NA | NO | NO | YES | NO | SPI | NA |
| 6 | 2005-016 | 19369-031 | 1000 | FL | 2005 | Mar | Hernando | *R. catesbeiana* | 26 | NA | NO | NO | YES | NO | SPI | NA |
| 6 | 2005-016 | 19369-030 | 1000 | FL | 2005 | Mar | Hernando | *R. catesbeiana* | 27 | NA | NO | NO | YES | NO | SPI | NA |
| 6 | 2005-016 | 19369-029 | 1000 | FL | 2005 | Mar | Hernando | *R. catesbeiana* | 29 | NA | NO | NO | YES | NO | SPI | NA |
| 6 | 2005-016 | 19369-028 | 1000 | FL | 2005 | Mar | Hernando | *R. catesbeiana* | 34 | NA | NO | NO | YES | NO | SPI | NA |
| 6 | 2005-016 | 19369-027 | 1000 | FL | 2005 | Mar | Hernando | *R. catesbeiana* | 39 | NA | NO | NO | YES | NO | SPI | NA |
| 6 | 2005-016 | 19369-026 | 1000 | FL | 2005 | Mar | Hernando | *R. catesbeiana* | 36 | NA | NO | NO | YES | NO | SPI | NA |
| 6 | 2005-016 | 19369-025 | 1000 | FL | 2005 | Mar | Hernando | *R. catesbeiana* | 39 | NA | NO | NO | YES | NO | SPI | NA |
| 6 | 2005-016 | 19369-024 | 1000 | FL | 2005 | Mar | Hernando | *R. catesbeiana* | 34 | NA | NO | NO | YES | NO | SPI | NA |
| 6 | 2005-016 | 19369-023 | 1000 | FL | 2005 | Mar | Hernando | *R. catesbeiana* | 26 | NA | NO | NO | YES | NO | SPI | NA |
| 6 | 2005-016 | 19369-022 | 1000 | FL | 2005 | Mar | Hernando | *R. catesbeiana* | 38 | NA | NO | NO | YES | NO | SPI | NA |
| 6 | 2005-016 | 19369-021 | 1000 | FL | 2005 | Mar | Hernando | *R. catesbeiana* | 39 | NA | NO | NO | YES | NO | SPI | NA |
| 6 | 2005-016 | 19369-019 | 1000 | FL | 2005 | Mar | Hernando | *R. catesbeiana* | 39 | NA | NO | NO | YES | NO | SPI | NA |
| 6 | 2005-016 | 19369-017 | 1000 | FL | 2005 | Mar | Hernando | *R. sphenocephala* | 26 | NA | NO | NO | YES | NO | SPI | NA |
| 6 | 2005-016 | 19369-015 | 1000 | FL | 2005 | Mar | Hernando | *R. sphenocephala* | 25 | NA | NO | NO | YES | NO | SPI | NA |
| 6 | 2005-016 | 19369-012 | 1000 | FL | 2005 | Mar | Hernando | *R. sphenocephala* | 27 | NA | NO | NO | YES | NO | SPI | NA |
| 6 | 2005-016 | 19369-010 | 1000 | FL | 2005 | Mar | Hernando | *R. sphenocephala* | 27 | NA | NO | NO | YES | NO | SPI | NA |
| 6 | 2005-016 | 19369-008 | 1000 | FL | 2005 | Mar | Hernando | *R. sphenocephala* | 27 | NA | NO | NO | YES | NO | SPI | NA |
| 6 | 2005-016 | 19369-007 | 1000 | FL | 2005 | Mar | Hernando | *R. sphenocephala* | 26 | NA | NO | NO | YES | NO | SPI | NA |
| 6 | 2005-016 | 19369-006 | 1000 | FL | 2005 | Mar | Hernando | *Rana capito* | 25 | NA | NO | YES | NO | NO | SPI | NA |
| 6 | 2005-016 | 19369-005 | 1000 | FL | 2005 | Mar | Hernando | *R. capito* | 25 | NA | NO | YES | NO | NO | SPI | NA |
| 6 | 2005-016 | 19369-004 | 1000 | FL | 2005 | Mar | Hernando | *R. capito* | 26 | NA | NO | YES | NO | NO | SPI | NA |
| 6 | 2005-016 | 19369-003 | 1000 | FL | 2005 | Mar | Hernando | *R. capito* | 26 | NA | NO | YES | NO | NO | SPI | NA |
| 6 | 2005-016 | 19369-002 | 1000 | FL | 2005 | Mar | Hernando | *R. capito* | 26 | POS | YES | YES | NO | NO | SPI | NA |
| 6 | 2005-016 | 19369-001 | 1000 | FL | 2005 | Mar | Hernando | *R. capito* | NA | NA | NO | YES | NO | NO | SPI | NA |
| 7 | 2005-028 | 19384-015 | 31 | FL | 2005 | Mar | Putnam | *R. sphenocephala* | 34 | NEG | YES | NA | NA | NA | Und | NA |
| 7 | 2005-028 | 19384-014 | 31 | FL | 2005 | Mar | Putnam | *R. sphenocephala* | 26 | NEG | YES | NA | NA | NA | Und | NA |
| 7 | 2005-028 | 19384-017 | 31 | FL | 2005 | Mar | Putnam | *R. sphenocephala* | 27 | NA | NO | YES | NO | NO | SPI | NA |
| 7 | 2005-028 | 19384-009 | 31 | FL | 2005 | Mar | Putnam | *R. sphenocephala* | 26 | POS | YES | YES | NO | NO | SPI | NA |
| 8 | NA | 44277-011 | NA | FL | 2010 | Mar | Wakulla | *R. sphenocephala* | 35 | POS | YES | NA | NA | NA | Und | NA |
| 8 | NA | 44277-006 | NA | FL | 2010 | Mar | Wakulla | *R. sphenocephala* | 38 | NEG | YES | NA | NA | NA | Und | NA |
| 8 | NA | 44277-002 | NA | FL | 2010 | Mar | Wakulla | *R. sphenocephala* | 34 | NEG | YES | NA | NA | NA | Und | NA |
| 8 | NA | 44277-012 | NA | FL | 2010 | Mar | Wakulla | *R. sphenocephala* | 35 | NA | NO | YES | NO | NO | SPI | NA |
| 8 | NA | 44277-008 | NA | FL | 2010 | Mar | Wakulla | *R. sphenocephala* | 37 | NA | NO | YES | NO | NO | SPI | NA |
| 8 | NA | 44277-004 | NA | FL | 2010 | Mar | Wakulla | *R. sphenocephala* | 40 | NA | NO | YES | NO | NO | SPI | NA |
| 8 | NA | 44277-003 | NA | FL | 2010 | Mar | Wakulla | *R. sphenocephala* | 33 | POS | YES | YES | NO | NO | SPI | NA |
| 8 | NA | 44277-001 | NA | FL | 2010 | Mar | Wakulla | *R. sphenocephala* | 32 | NA | NO | YES | NO | NO | SPI | NA |
| 9 | 2015-019 | 26186-005 | 26 | FL | 2015 | Mar | Okaloosa | *R. sphenocephala* | NA | NA | NO | YES | NO | NO | SPI | Chytridiomycosis, Saprolegniasis |
| 10 | 2006-012 | 19709-005 | 12 | GA | 2006 | Mar | Miller | *Rana heckscheri* | 37 | NA | NO | NA | NA | NA | Und | NA |
| 10 | 2006-012 | 19709-007 | 12 | GA | 2006 | Mar | Miller | *R. heckscheri* | 36 | NA | NO | YES | NO | NO | SPI | Saprolegniasis |
| 10 | 2006-012 | 19709-006 | 12 | GA | 2006 | Mar | Miller | *R. heckscheri* | 36 | POS | YES | YES | NO | NO | SPI | Saprolegniasis |
| 10 | 2006-012 | 19709-003 | 12 | GA | 2006 | Mar | Miller | *R. heckscheri* | 37 | NA | NO | YES | NO | NO | SPI | Ranavirus (isolated) |
| 10 | 2006-012 | 19709-001 | 12 | GA | 2006 | Mar | Miller | *R. heckscheri* | 36 | NA | NO | YES | NO | NO | SPI | NA |
| 10 | 2006-012 | 19709-002 | 12 | GA | 2006 | Mar | Miller | *R. heckscheri* | 38 | NA | NO | NA | NA | NA | Ranavirus (isolated) | NA |
| 11 | 2003-034 | 18626-007 | 500 | LA | 2003 | Apr | St. Tammany | *R. sphenocephala* | 42 | NEG | YES | NA | NA | NA | Und | NA |
| 11 | 2003-034 | 18626-005 | 500 | LA | 2003 | Apr | St. Tammany | *R. sphenocephala* | 40 | NEG | YES | NA | NA | NA | Und | NA |
| 11 | 2003-034 | 18626-004 | 500 | LA | 2003 | Apr | St. Tammany | *R. sphenocephala* | 39 | NEG | YES | NA | NA | NA | Und | NA |
| 11 | 2003-034 | 18626-003 | 500 | LA | 2003 | Apr | St. Tammany | *R. sphenocephala* | 37 | NEG | YES | NA | NA | NA | Und | NA |
| 11 | 2003-034 | 18626-002 | 500 | LA | 2003 | Apr | St. Tammany | *R. sphenocephala* | 37 | NEG | YES | NA | NA | NA | Und | NA |
| 11 | 2003-034 | 18626-001 | 500 | LA | 2003 | Apr | St. Tammany | *R. sphenocephala* | 35 | NEG | YES | NA | NA | NA | Und | NA |
| 11 | 2003-034 | 18626-010 | 500 | LA | 2003 | Apr | St. Tammany | *R. sphenocephala* | 35 | NA | NO | YES | NO | NO | SPI | NA |
| 12 | NA | 4957-187 | NA | LA | 2005 | Apr | St. Martin | *R. sphenocephala* | 35 | NEG | YES | NA | NA | NA | Und | NA |
| 12 | NA | 4957-186 | NA | LA | 2005 | Apr | St. Martin | *R. sphenocephala* | 35 | NEG | YES | NA | NA | NA | Und | NA |
| 12 | NA | 4957-139 | NA | LA | 2005 | Mar | St. Martin | *A. gryllus* | 36 | NA | NO | NO | YES | NO | SPI | NA |
| 13 | NA | 4957-323 | NA | LA | 2005 | May | Union | *R. sphenocephala* | 35 | NA | NO | YES | NO | NO | SPI | NA |
| 13 | NA | 4957-322 | NA | LA | 2005 | May | Union | *R. sphenocephala* | 34 | NA | NO | YES | NO | NO | SPI | NA |
| 13 | NA | 4957-319 | NA | LA | 2005 | May | Union | *R. sphenocephala* | 35 | NA | NO | YES | NO | NO | SPI | NA |
| 13 | NA | 4957-318 | NA | LA | 2005 | May | Union | *R. sphenocephala* | 33 | NA | NO | YES | NO | NO | SPI | NA |
| 13 | NA | 4957-312 | NA | LA | 2005 | May | Union | *R. sphenocephala* | 36 | NA | NO | YES | NO | NO | SPI | NA |
| 13 | NA | 4957-311 | NA | LA | 2005 | May | Union | *R. sphenocephala* | 35 | POS | YES | YES | NO | NO | SPI | NA |
| 13 | NA | 4957-288 | NA | LA | 2005 | May | Union | *R. sphenocephala* | 35 | NA | NO | YES | NO | NO | SPI | NA |
| 14 | 2003-068 | 18761-012 | 1000 | MD | 2003 | Aug | Prince George's | *R. sphenocephala* | 45 | NA | NO | NO | YES | NO | SPI | NA |
| 14 | 2003-068 | 18761-005 | 1000 | MD | 2003 | Aug | Prince George's | *R. sphenocephala* | 46 | POS | YES | NO | YES | NO | SPI | NA |
| 14 | 2003-068 | 18761-004 | 1000 | MD | 2003 | Aug | Prince George's | *R. sphenocephala* | 46 | NA | NO | NO | YES | NO | SPI | NA |
| 14 | 2003-068 | 18709-018 | 1000 | MD | 2003 | Jul | Prince George's | *R. sphenocephala* | 41 | NA | NO | YES | NO | NO | SPI | NA |
| 14 | 2003-068 | 18709-017 | 1000 | MD | 2003 | Jul | Prince George's | *R. sphenocephala* | 45 | NA | NO | YES | NO | NO | SPI | NA |
| 14 | 2003-068 | 18709-016 | 1000 | MD | 2003 | Jul | Prince George's | *R. sphenocephala* | 41 | NA | NO | YES | NO | NO | SPI | NA |
| 14 | 2003-068 | 18709-015 | 1000 | MD | 2003 | Jul | Prince George's | *R. sphenocephala* | 41 | NA | NO | YES | NO | NO | SPI | NA |
| 14 | 2003-068 | 18709-014 | 1000 | MD | 2003 | Jul | Prince George's | *R. sphenocephala* | 41 | NA | NO | YES | NO | NO | SPI | NA |
| 14 | 2003-068 | 18709-006 | 1000 | MD | 2003 | Jul | Prince George's | *R. sphenocephala* | 41 | NA | NO | YES | NO | NO | SPI | NA |
| 14 | 2003-068 | 18709-005 | 1000 | MD | 2003 | Jul | Prince George's | *R. sphenocephala* | 42 | NA | NO | YES | NO | NO | SPI | NA |
| 14 | 2003-068 | 18709-004 | 1000 | MD | 2003 | Jul | Prince George's | *R. sphenocephala* | 42 | NA | NO | YES | NO | NO | SPI | NA |
| 14 | 2003-068 | 18709-003 | 1000 | MD | 2003 | Jul | Prince George's | *R. sphenocephala* | 43 | NA | NO | YES | NO | NO | SPI | NA |
| 14 | 2003-068 | 18709-002 | 1000 | MD | 2003 | Jul | Prince George's | *R. sphenocephala* | 41 | POS | YES | YES | NO | NO | SPI | NA |
| 14 | 2003-068 | 18709-001 | 1000 | MD | 2003 | Jul | Prince George's | *R. sphenocephala* | 41 | NA | NO | YES | NO | NO | SPI | NA |
| 15 | NA | 4824-454 | NA | ME | 2003 | Jul | Hancock | *Rana clamitans* | 46 | NEG | YES | NA | NA | NA | Und | NA |
| 15 | NA | 4824-375 | NA | ME | 2003 | Jun | Hancock | *R. catesbeiana* | 46 | NEG | YES | NA | NA | NA | Und | NA |
| 15 | NA | 4824-339 | NA | ME | 2003 | Jun | Hancock | *R. sylvatica* | 34 | NEG | YES | NA | NA | NA | Und | NA |
| 15 | NA | 4824-329 | NA | ME | 2003 | Jun | Hancock | *R. sylvatica* | 34 | NEG | YES | NA | NA | NA | Und | NA |
| 15 | NA | 4824-327 | NA | ME | 2003 | Jun | Hancock | *R. sylvatica* | 38 | NEG | YES | NA | NA | NA | Und | NA |
| 15 | NA | 4824-326 | NA | ME | 2003 | Jun | Hancock | *R. sylvatica* | 38 | NEG | YES | NA | NA | NA | Und | NA |
| 15 | NA | 4824-303 | NA | ME | 2003 | Jun | Hancock | *R. sylvatica* | 34 | NEG | YES | NA | NA | NA | Und | NA |
| 15 | NA | 4824-181 | NA | ME | 2003 | May | Hancock | *R. sylvatica* | NA | NEG | YES | NA | NA | NA | Und | NA |
| 15 | NA | 4824-508 | NA | ME | 2003 | Jul | Hancock | *Pseudacris crucifer* | 38 | NA | NO | YES | NO | NO | SPI | NA |
| 15 | NA | 4824-484 | NA | ME | 2003 | Aug | Hancock | *R. clamitans* | 27 | NA | NO | NO | YES | NO | SPI | NA |
| 15 | NA | 4824-468 | NA | ME | 2003 | Aug | Hancock | *R. clamitans* | 29 | NA | NO | NO | YES | NO | SPI | NA |
| 15 | NA | 4824-467 | NA | ME | 2003 | Aug | Hancock | *R. clamitans* | 28 | NA | NO | NO | NO | YES | SPI | NA |
| 15 | NA | 4824-466 | NA | ME | 2003 | Aug | Hancock | *R. clamitans* | 28 | NA | NO | NO | YES | NO | SPI | NA |
| 15 | NA | 4824-464 | NA | ME | 2003 | Aug | Hancock | *R. clamitans* | 28 | NA | NO | NO | YES | NO | SPI | NA |
| 15 | NA | 4824-463 | NA | ME | 2003 | Aug | Hancock | *R. clamitans* | 27 | POS | YES | NO | NO | YES | SPI | NA |
| 15 | NA | 4824-457 | NA | ME | 2003 | Aug | Hancock | *A. gryllus* | 38 | NA | NO | NO | NO | YES | SPI | NA |
| 15 | NA | 4824-453 | NA | ME | 2003 | Jul | Hancock | *R. catesbeiana* | 27 | NA | NO | NO | YES | NO | SPI | NA |
| 15 | NA | 4824-452 | NA | ME | 2003 | Jul | Hancock | *R. catesbeiana* | 25 | NA | NO | NO | YES | NO | SPI | NA |
| 15 | NA | 4824-451 | NA | ME | 2003 | Jul | Hancock | *R. catesbeiana* | 38 | NA | NO | NO | NO | YES | SPI | NA |
| 15 | NA | 4824-406 | NA | ME | 2003 | Jul | Hancock | *R. catesbeiana* | NA | NA | NO | NO | YES | NO | SPI | NA |
| 15 | NA | 4824-379 | NA | ME | 2003 | Jul | Hancock | *R. sylvatica* | 46 | NA | NO | NO | NO | YES | SPI | NA |
| 15 | NA | 4824-370 | NA | ME | 2003 | Jun | Hancock | *R. catesbeiana* | 33 | NA | NO | NO | NO | YES | SPI | NA |
| 15 | NA | 4824-369 | NA | ME | 2003 | Jun | Hancock | *R. catesbeiana* | 26 | NA | NO | NO | NO | YES | SPI | NA |
| 15 | NA | 4824-341 | NA | ME | 2003 | Jun | Hancock | *R. sylvatica* | 38 | POS | YES | NO | NO | YES | SPI | NA |
| 15 | NA | 4824-368 | NA | ME | 2003 | Jun | Hancock | *R. catesbeiana* | 29 | NA | NO | NA | NA | NA | Ranavirus (isolated) | NA |
| 15 | NA | 4824-367 | NA | ME | 2003 | Jun | Hancock | *R. catesbeiana* | 27 | NA | NO | NA | NA | NA | Ranavirus (isolated) | NA |
| 16 | NA | 4911-278 | NA | ME | 2004 | Jul | Hancock | *R. sylvatica* | 46 | NA | NO | NO | YES | NO | SPI | NA |
| 16 | NA | 4911-275 | NA | ME | 2004 | Jul | Hancock | *A. gryllus* | 37 | NA | NO | NO | YES | NO | SPI | NA |
| 16 | NA | 4911-269 | NA | ME | 2004 | Jul | Hancock | *A. gryllus* | 41 | NA | NO | NO | YES | NO | SPI | NA |
| 16 | NA | 4911-158 | NA | ME | 2004 | Jun | Hancock | *R. catesbeiana* | 39 | NA | NO | NO | YES | NO | SPI | NA |
| 17 | NA | 4960-244 | NA | ME | 2005 | Aug | Hancock | *R. clamitans* | 27 | NEG | YES | NA | NA | NA | Und | NA |
| 17 | NA | 4960-243 | NA | ME | 2005 | Aug | Hancock | *R. clamitans* | 26 | NEG | YES | NA | NA | NA | Und | NA |
| 17 | NA | 4960-295 | NA | ME | 2005 | Aug | Hancock | *R. clamitans* | 25 | NA | NO | YES | NO | NO | SPI | NA |
| 17 | NA | 4960-291 | NA | ME | 2005 | Aug | Hancock | *R. clamitans* | 27 | POS | YES | YES | NO | NO | SPI | NA |
| 17 | NA | 4960-290 | NA | ME | 2005 | Aug | Hancock | *R. clamitans* | 45 | NA | NO | YES | NO | NO | SPI | NA |
| 17 | NA | 4960-265 | NA | ME | 2005 | Aug | Hancock | *R. clamitans* | 25 | NA | NO | YES | NO | NO | SPI | NA |
| 17 | NA | 4960-224 | NA | ME | 2005 | Jul | Hancock | *R. clamitans* | 44 | NA | NO | NO | NO | YES | SPI | NA |
| 17 | NA | 4960-183 | NA | ME | 2005 | Jul | Hancock | *R. clamitans* | 44 | NA | NO | YES | NO | NO | SPI | NA |
| 17 | NA | 4960-182 | NA | ME | 2005 | Jul | Hancock | *R. clamitans* | 44 | NA | NO | YES | NO | NO | SPI | NA |
| 18 | 2000-073 | 16869-003 | 100 | MN | 2000 | Jun | Crow Wing | *R. clamitans* | 35 | NA | NO | YES | NO | NO | SPI | NA |
| 18 | 2000-073 | 16869-002 | 100 | MN | 2000 | Jun | Crow Wing | *R. clamitans* | 37 | NA | NO | YES | NO | NO | SPI | NA |
| 18 | 2000-073 | 16869-001 | 100 | MN | 2000 | Jun | Crow Wing | *Rana septentrionalis* | 38 | NA | NO | YES | NO | NO | SPI | NA |
| 18 | 2000-073 | 17023-032 | 100 | MN | 2000 | Jun | Crow Wing | *R. septentrionalis* | 35 | NA | NO | YES | NO | NO | SPI | NA |
| 18 | 2000-073 | 17023-031 | 100 | MN | 2000 | Jun | Crow Wing | *R. septentrionalis* | 26 | NA | NO | YES | NO | NO | SPI | NA |
| 18 | 2000-073 | 17023-030 | 100 | MN | 2000 | Jun | Crow Wing | *R. septentrionalis* | 41 | NA | NO | YES | NO | NO | SPI | NA |
| 18 | 2000-073 | 17023-023 | 100 | MN | 2000 | Jun | Crow Wing | *R. septentrionalis* | 40 | NA | NO | YES | NO | NO | SPI | NA |
| 18 | 2000-073 | 17023-022 | 100 | MN | 2000 | Jul | Crow Wing | *R. clamitans* | 29 | NA | NO | YES | NO | NO | SPI | NA |
| 18 | 2000-073 | 17023-021 | 100 | MN | 2000 | Jul | Crow Wing | *R. clamitans* | 34 | NA | NO | YES | NO | NO | SPI | NA |
| 18 | 2000-073 | 17023-020 | 100 | MN | 2000 | Jul | Crow Wing | *R. clamitans* | 35 | NA | NO | YES | NO | NO | SPI | NA |
| 18 | 2000-073 | 17023-019 | 100 | MN | 2000 | Jun | Crow Wing | *R. clamitans* | 35 | NA | NO | YES | NO | NO | SPI | NA |
| 18 | 2000-073 | 17023-018 | 100 | MN | 2000 | Jun | Crow Wing | *R. clamitans* | 36 | NA | NO | YES | NO | NO | SPI | NA |
| 18 | 2000-073 | 17023-017 | 100 | MN | 2000 | Jun | Crow Wing | *R. clamitans* | 27 | NA | NO | YES | NO | NO | SPI | Chytridiomycosis |
| 18 | 2000-073 | 17023-016 | 100 | MN | 2000 | Jun | Crow Wing | *R. clamitans* | 40 | NA | NO | YES | NO | NO | SPI | NA |
| 18 | 2000-073 | 17023-015 | 100 | MN | 2000 | Jun | Crow Wing | *R. clamitans* | 40 | NA | NO | YES | NO | NO | SPI | NA |
| 18 | 2000-073 | 17023-014 | 100 | MN | 2000 | Jun | Crow Wing | *R. clamitans* | 40 | NA | NO | YES | NO | NO | SPI | NA |
| 18 | 2000-073 | 17023-013 | 100 | MN | 2000 | Jun | Crow Wing | *Rana pipiens* | NA | NA | NO | YES | NO | NO | SPI | NA |
| 18 | 2000-073 | 17023-012 | 100 | MN | 2000 | Jun | Crow Wing | *R. pipiens* | 28 | NA | NO | YES | NO | NO | SPI | Chytridiomycosis |
| 18 | 2000-073 | 17023-011 | 100 | MN | 2000 | Jul | Crow Wing | *R. clamitans* | 28 | NA | NO | YES | NO | NO | SPI | NA |
| 18 | 2000-073 | 17023-006 | 100 | MN | 2000 | Jun | Crow Wing | *R. clamitans* | 37 | NA | NO | YES | NO | NO | SPI | NA |
| 18 | 2000-073 | 17023-002 | 100 | MN | 2000 | Jun | Crow Wing | *R. septentrionalis* | NA | NA | NO | YES | NO | NO | SPI | NA |
| 18 | 2000-073 | 17023-001 | 100 | MN | 2000 | Jun | Crow Wing | *R. septentrionalis* | 41 | NA | NO | YES | NO | NO | SPI | NA |
| 18 | 2000-073 | 17023-035 | 100 | MN | 2000 | Jul | Crow Wing | *R. septentrionalis* | 45 | NA | NO | NA | NA | NA | Ranavirus suspected based on histopathology | NA |
| 18 | 2000-073 | 17023-034 | 100 | MN | 2000 | Jul | Crow Wing | *R. septentrionalis* | 45 | NA | NO | NA | NA | NA | Ranavirus suspected based on histopathology | NA |
| 18 | 2000-073 | 17023-028 | 100 | MN | 2000 | Jun | Crow Wing | *R. septentrionalis* | NA | NA | NO | NA | NA | NA | Ranavirus suspected based on histopathology | NA |
| 18 | 2000-073 | 17023-027 | 100 | MN | 2000 | Jun | Crow Wing | *R. septentrionalis* | 38 | NA | NO | NA | NA | NA | Ranavirus suspected based on histopathology | NA |
| 18 | 2000-073 | 17023-026 | 100 | MN | 2000 | Jun | Crow Wing | *R. septentrionalis* | 30 | NA | NO | NA | NA | NA | Ranavirus suspected based on histopathology | NA |
| 19 | 2001-108 | 17161-006 | 10 | MS | 2001 | Mar | Harrison | *R. sphenocephala* | 30 | NA | NO | NO | NO | YES | SPI | NA |
| 19 | 2001-108 | 17161-005 | 10 | MS | 2001 | Mar | Harrison | *R. sphenocephala* | 28 | NA | NO | YES | NO | NO | SPI | NA |
| 19 | 2001-108 | 17161-004 | 10 | MS | 2001 | Mar | Harrison | *R. sphenocephala* | 30 | NA | NO | YES | NO | NO | SPI | NA |
| 19 | 2001-108 | 17161-003 | 10 | MS | 2001 | Mar | Harrison | *R. sphenocephala* | 30 | NA | NO | YES | NO | NO | SPI | NA |
| 19 | 2001-108 | 17161-002 | 10 | MS | 2001 | Mar | Harrison | *R. sphenocephala* | 31 | NA | NO | YES | NO | NO | SPI | NA |
| 19 | 2001-108 | 17161-001 | 10 | MS | 2001 | Mar | Harrison | *R. sphenocephala* | 34 | NA | NO | YES | NO | NO | SPI | NA |
| 20 | 2003-021 | 18613-022 | 1000 | MS | 2003 | Apr | Harrison | *R. sphenocephala* | NA | NEG | YES | NA | NA | NA | Und | NA |
| 20 | 2003-021 | 18610-057 | 1000 | MS | 2003 | May | Harrison | *R. sphenocephala* | 35 | NEG | YES | NA | NA | NA | Und | NA |
| 20 | 2003-021 | 18642-005 | 1000 | MS | 2003 | May | Harrison | *R. sphenocephala* | 26 | NA | NO | YES | NO | NO | SPI | NA |
| 20 | 2003-021 | 18613-021 | 1000 | MS | 2003 | Apr | Harrison | *R. sphenocephala* | 36 | NA | NO | YES | NO | NO | SPI | NA |
| 20 | NA | 18612-030 | 1000 | MS | 2003 | May | Harrison | *Rana sevosa* | 31 | NA | NO | YES | NO | NO | SPI | NA |
| 20 | NA | 18612-029 | 1000 | MS | 2003 | May | Harrison | *R. sevosa* | 29 | NA | NO | YES | NO | NO | SPI | NA |
| 20 | NA | 18612-028 | 1000 | MS | 2003 | May | Harrison | *R. sevosa* | 30 | NA | NO | YES | NO | NO | SPI | NA |
| 20 | NA | 18612-027 | 1000 | MS | 2003 | May | Harrison | *R. sevosa* | 30 | NA | NO | YES | NO | NO | SPI | NA |
| 20 | NA | 18612-026 | 1000 | MS | 2003 | May | Harrison | *R. sevosa* | 34 | NA | NO | YES | NO | NO | SPI | NA |
| 20 | NA | 18612-020 | 1000 | MS | 2003 | May | Harrison | *R. sevosa* | 27 | NA | NO | NO | NO | YES | SPI | NA |
| 20 | NA | 18612-018 | 1000 | MS | 2003 | May | Harrison | *R. sevosa* | 28 | NA | NO | NO | NO | YES | SPI | NA |
| 20 | NA | 18612-013 | 1000 | MS | 2003 | Apr | Harrison | *R. sevosa* | 33 | POS | YES | YES | NO | NO | SPI | NA |
| 20 | 2003-021 | 18611-009 | 1000 | MS | 2003 | Apr | Harrison | *R. sphenocephala* | 26 | NA | NO | YES | NO | NO | SPI | NA |
| 20 | 2003-021 | 18611-008 | 1000 | MS | 2003 | Apr | Harrison | *R. sphenocephala* | 29 | NA | NO | YES | NO | NO | SPI | NA |
| 20 | 2003-021 | 18611-007 | 1000 | MS | 2003 | Apr | Harrison | *R. sphenocephala* | NA | NA | NO | YES | NO | NO | SPI | NA |
| 20 | 2003-021 | 18611-006 | 1000 | MS | 2003 | Apr | Harrison | *R. sphenocephala* | 32 | NA | NO | YES | NO | NO | SPI | NA |
| 20 | 2003-021 | 18611-005 | 1000 | MS | 2003 | Apr | Harrison | *R. sphenocephala* | 31 | NA | NO | YES | NO | NO | SPI | NA |
| 20 | 2003-021 | 18611-004 | 1000 | MS | 2003 | Apr | Harrison | *R. sphenocephala* | 31 | NA | NO | YES | NO | NO | SPI | NA |
| 20 | 2003-021 | 18610-016 | 1000 | MS | 2003 | Apr | Harrison | *R. sphenocephala* | 26 | NA | NO | YES | NO | NO | SPI | NA |
| 20 | 2003-021 | 18587-014 | 1000 | MS | 2003 | Mar | Harrison | *R. sphenocephala* | 33 | NA | NO | YES | NO | NO | SPI | NA |
| 20 | 2003-021 | 18587-013 | 1000 | MS | 2003 | Mar | Harrison | *R. sphenocephala* | 32 | NA | NO | NO | NO | YES | SPI | NA |
| 20 | 2003-021 | 18587-012 | 1000 | MS | 2003 | Mar | Harrison | *R. sphenocephala* | 31 | NA | NO | NO | NO | YES | SPI | NA |
| 20 | 2003-021 | 18587-011 | 1000 | MS | 2003 | Mar | Harrison | *R. sphenocephala* | NA | NA | NO | NO | NO | YES | SPI | NA |
| 20 | 2003-021 | 18587-008 | 1000 | MS | 2003 | Mar | Harrison | *R. sevosa* | 28 | NA | NO | YES | NO | NO | SPI | NA |
| 20 | 2003-021 | 18587-007 | 1000 | MS | 2003 | Mar | Harrison | *R. sevosa* | 32 | NA | NO | YES | NO | NO | SPI | NA |
| 20 | 2003-021 | 18587-006 | 1000 | MS | 2003 | Mar | Harrison | *R. sevosa* | 32 | NA | NO | YES | NO | NO | SPI | NA |
| 20 | 2003-021 | 18587-005 | 1000 | MS | 2003 | Mar | Harrison | *R. sevosa* | 25 | POS | YES | YES | NO | NO | SPI | NA |
| 20 | 2003-021 | 18587-004 | 1000 | MS | 2003 | Mar | Harrison | *R. sevosa* | 30 | NA | NO | YES | NO | NO | SPI | NA |
| 20 | 2003-021 | 18587-003 | 1000 | MS | 2003 | Mar | Harrison | *R. sevosa* | 31 | NA | NO | YES | NO | NO | SPI | NA |
| 20 | 2003-021 | 18587-002 | 1000 | MS | 2003 | Mar | Harrison | *R. sevosa* | 33 | NA | NO | YES | NO | NO | SPI | NA |
| 20 | 2003-021 | 18587-001 | 1000 | MS | 2003 | Mar | Harrison | *R. sevosa* | 32 | NA | NO | YES | NO | NO | SPI | NA |
| 21 | 2004-011 | 18967-006 | 30 | MS | 2004 | Feb | Harrison | *R. sphenocephala* | 26 | NA | NO | YES | NO | NO | SPI | NA |
| 21 | 2004-011 | 18967-005 | 30 | MS | 2004 | Feb | Harrison | *R. sphenocephala* | 27 | NA | NO | YES | NO | NO | SPI | NA |
| 21 | 2004-011 | 18967-004 | 30 | MS | 2004 | Feb | Harrison | *R. sphenocephala* | 26 | NA | NO | YES | NO | NO | SPI | NA |
| 21 | 2004-011 | 18967-003 | 30 | MS | 2004 | Feb | Harrison | *R. sphenocephala* | 28 | NA | NO | YES | NO | NO | SPI | NA |
| 21 | 2004-011 | 18967-002 | 30 | MS | 2004 | Feb | Harrison | *R. sphenocephala* | 25 | NA | NO | YES | NO | NO | SPI | NA |
| 21 | 2004-011 | 18967-001 | 30 | MS | 2004 | Feb | Harrison | *R. sphenocephala* | 26 | NA | NO | YES | NO | NO | SPI | NA |
| 22 | 2014-189 | 24988-009 | 39 | MS | 2014 | Jul | Harrison | *R. sevosa* | 39 | NA | NO | YES | NO | NO | SPI | NA |
| 22 | 2014-189 | 24988-008 | 39 | MS | 2014 | Jun | Harrison | *R. sevosa* | 44 | NA | NO | YES | NO | NO | SPI | NA |
| 22 | 2014-189 | 24988-002 | 39 | MS | 2014 | May | Harrison | *R. sevosa* | 31 | POS | YES | YES | NO | NO | SPI | NA |
| 23 | 1999-110 | 16407-011 | 100 | NH | 1999 | Sep | Carroll | *R. catesbeiana* | 35 | NA | NO | YES | NO | NO | SPI | NA |
| 23 | 1999-110 | 16407-010 | 100 | NH | 1999 | Sep | Carroll | *R. catesbeiana* | 35 | NA | NO | YES | NO | NO | SPI | NA |
| 23 | 1999-110 | 16407-009 | 100 | NH | 1999 | Sep | Carroll | *R. catesbeiana* | 36 | NA | NO | YES | NO | NO | SPI | Chytridiomycosis |
| 23 | 1999-110 | 16407-008 | 100 | NH | 1999 | Sep | Carroll | *R. catesbeiana* | 35 | NA | NO | YES | NO | NO | SPI | NA |
| 23 | 1999-110 | 16407-007 | 100 | NH | 1999 | Sep | Carroll | *R. catesbeiana* | 35 | NA | NO | YES | NO | NO | SPI | NA |
| 23 | 1999-110 | 16407-006 | 100 | NH | 1999 | Sep | Carroll | *R. catesbeiana* | 35 | NA | NO | YES | NO | NO | SPI | NA |
| 23 | 1999-110 | 16407-005 | 100 | NH | 1999 | Sep | Carroll | *R. catesbeiana* | 35 | NA | NO | YES | NO | NO | SPI | NA |
| 23 | 1999-110 | 16407-004 | 100 | NH | 1999 | Sep | Carroll | *R. catesbeiana* | 35 | NA | NO | YES | NO | NO | SPI | NA |
| 23 | 1999-110 | 16407-003 | 100 | NH | 1999 | Sep | Carroll | *R. catesbeiana* | 35 | POS | YES | YES | NO | NO | SPI | NA |
| 23 | 1999-110 | 16407-002 | 100 | NH | 1999 | Sep | Carroll | *R. catesbeiana* | 35 | NA | NO | YES | NO | NO | SPI | NA |
| 23 | 1999-110 | 16407-001 | 100 | NH | 1999 | Sep | Carroll | *R. catesbeiana* | 36 | NA | NO | YES | NO | NO | SPI | NA |
| 24 | 2008-216 | 20626-003 | 180 | NY | 2007 | Nov | Suffolk | *R. sphenocephala* | 28 | NA | NO | YES | NO | NO | SPI | NA |
| 24 | 2008-216 | 20626-002 | 180 | NY | 2007 | Nov | Suffolk | *R. sphenocephala* | 28 | NA | NO | YES | NO | NO | SPI | NA |
| 24 | 2008-216 | 20626-001 | 180 | NY | 2007 | Nov | Suffolk | *R. sphenocephala* | NA | NA | NO | YES | NO | NO | SPI | NA |
| 24 | 2008-216 | 20626-010 | 180 | NY | 2007 | Oct | Suffolk | *R. sphenocephala* | 46 | NA | NO | NA | NA | NA | Ranavirus suspected based on histopathology | NA |
| 24 | 2008-216 | 20626-009 | 180 | NY | 2007 | Oct | Suffolk | *R. sphenocephala* | 46 | NA | NO | NA | NA | NA | Ranavirus suspected based on histopathology | NA |
| 24 | 2008-216 | 20626-008 | 180 | NY | 2007 | Oct | Suffolk | *R. sphenocephala* | 46 | NA | NO | NA | NA | NA | Ranavirus suspected based on histopathology | NA |
| 24 | 2008-216 | 20626-014 | 180 | NY | 2007 | Nov | Suffolk | *R. sphenocephala* | 37 | NA | NO | NA | NA | NA | Chytridiomycosis | NA |
| 24 | 2008-216 | 20626-013 | 180 | NY | 2007 | Nov | Suffolk | *R. sphenocephala* | 36 | NA | NO | NA | NA | NA | Chytridiomycosis | NA |
| 24 | 2008-216 | 20626-012 | 180 | NY | 2007 | Oct | Suffolk | *R. sphenocephala* | 46 | NA | NO | NA | NA | NA | Chytridiomycosis | NA |
| 24 | 2008-216 | 20626-011 | 180 | NY | 2007 | Nov | Suffolk | *R. sphenocephala* | 46 | NA | NO | NA | NA | NA | Chytridiomycosis | NA |
| 25 | 2008-220 | 22445-005 | 165 | NY | 2007 | Jul | Suffolk | *R. sphenocephala* | 36 | NA | NO | YES | NO | NO | SPI | NA |
| 25 | 2008-220 | 22445-004 | 165 | NY | 2007 | Jul | Suffolk | *R. sphenocephala* | 43 | POS | YES | YES | NO | NO | SPI | NA |
| 25 | 2008-220 | 22445-006 | 165 | NY | 2008 | Jul | Suffolk | *R. sphenocephala* | 37 | NA | NO | YES | NO | NO | SPI | NA |
| 25 | 2008-220 | 22445-002 | 165 | NY | 2008 | Sep | Suffolk | *R. sphenocephala* | 46 | NA | NO | NA | NA | NA | Chytridiomycosis | NA |
| 25 | 2008-220 | 22445-001 | 165 | NY | 2008 | Sep | Suffolk | *R. sphenocephala* | 46 | NA | NO | NA | NA | NA | Chytridiomycosis | NA |
| 26 | 2009-075 | 22916-017 | 34 | NY | 2009 | Nov | Suffolk | *R. catesbeiana* | 26 | NA | NO | YES | NO | NO | SPI | NA |
| 26 | 2009-075 | 22916-016 | 34 | NY | 2009 | Sep | Suffolk | *R. sphenocephala* | 37 | NA | NO | YES | NO | NO | SPI | NA |
| 26 | 2009-075 | 22916-015 | 34 | NY | 2009 | Sep | Suffolk | *R. sphenocephala* | 25 | NA | NO | YES | NO | NO | SPI | NA |
| 26 | 2009-075 | 22916-014 | 34 | NY | 2009 | Sep | Suffolk | *R. sphenocephala* | 37 | NA | NO | YES | NO | NO | SPI | NA |
| 26 | 2009-075 | 22916-013 | 34 | NY | 2009 | Jul | Suffolk | *R. sphenocephala* | 27 | NA | NO | YES | NO | NO | SPI | NA |
| 26 | 2009-075 | 22916-006 | 34 | NY | 2009 | Jul | Suffolk | *R. sphenocephala* | 39 | NA | NO | YES | NO | NO | SPI | NA |
| 26 | 2009-075 | 22916-005 | 34 | NY | 2009 | Jul | Suffolk | *R. sphenocephala* | 42 | NA | NO | YES | NO | NO | SPI | NA |
| 26 | 2009-075 | 22916-004 | 34 | NY | 2009 | Jul | Suffolk | *R. sphenocephala* | 41 | NA | NO | YES | NO | NO | SPI | NA |
| 26 | 2009-075 | 22916-003 | 34 | NY | 2009 | Jul | Suffolk | *R. sphenocephala* | 43 | NA | NO | YES | NO | NO | SPI | NA |
| 26 | 2009-075 | 22916-002 | 34 | NY | 2009 | Jul | Suffolk | *R. sphenocephala* | 33 | NA | NO | YES | NO | NO | SPI | NA |
| 26 | 2009-075 | 22916-001 | 34 | NY | 2009 | Jul | Suffolk | *R. sphenocephala* | 34 | NA | NO | YES | NO | NO | SPI | NA |
| 27 | NA | 44276-040 | NA | OR | 2007 | May | Marion | *R. catesbeiana* | 28 | NA | NO | YES | NO | NO | SPI | NA |
| 27 | NA | 44276-039 | NA | OR | 2007 | May | Marion | *R. catesbeiana* | 32 | NA | NO | NA | NA | NA | Chytridiomycosis | NA |
| 28 | 1999-179 | 18029-011 | 19 | VA | 1999 | Jun | Henrico | *R. catesbeiana* | 37 | NA | NO | YES | NO | NO | SPI | Chytridiomycosis |
| 28 | 1999-179 | 18029-007 | 19 | VA | 1999 | Jun | Henrico | *R. catesbeiana* | 34 | NA | NO | YES | NO | NO | SPI | Chytridiomycosis |
| 28 | 1999-179 | 18029-005 | 19 | VA | 1999 | Jun | Henrico | *R. catesbeiana* | 38 | NA | NO | YES | NO | NO | SPI | Chytridiomycosis |
| 28 | 1999-179 | 18029-019 | 19 | VA | 1999 | Jun | Henrico | *R. catesbeiana* | 39 | NA | NO | NA | NA | NA | Chytridiomycosis | NA |
| 28 | 1999-179 | 18029-018 | 19 | VA | 1999 | Jun | Henrico | *R. catesbeiana* | 35 | NA | NO | NA | NA | NA | Chytridiomycosis | NA |
| 28 | 1999-179 | 18029-017 | 19 | VA | 1999 | Jun | Henrico | *R. catesbeiana* | 35 | NA | NO | NA | NA | NA | Chytridiomycosis | NA |
| 28 | 1999-179 | 18029-016 | 19 | VA | 1999 | Jun | Henrico | *R. catesbeiana* | 40 | NA | NO | NA | NA | NA | Chytridiomycosis | NA |
| 28 | 1999-179 | 18029-015 | 19 | VA | 1999 | Jun | Henrico | *R. catesbeiana* | 46 | NA | NO | NA | NA | NA | Chytridiomycosis | NA |
| 28 | 1999-179 | 18029-014 | 19 | VA | 1999 | Jun | Henrico | *R. catesbeiana* | 40 | NA | NO | NA | NA | NA | Chytridiomycosis | NA |
| 28 | 1999-179 | 18029-013 | 19 | VA | 1999 | Jun | Henrico | *R. catesbeiana* | 30 | NA | NO | NA | NA | NA | Chytridiomycosis | NA |
| 28 | 1999-179 | 18029-012 | 19 | VA | 1999 | Jun | Henrico | *R. catesbeiana* | 41 | NA | NO | NA | NA | NA | Chytridiomycosis | NA |
| 28 | 1999-179 | 18029-009 | 19 | VA | 1999 | Jun | Henrico | *R. catesbeiana* | 34 | NA | NO | NA | NA | NA | Chytridiomycosis | NA |
| 28 | 1999-179 | 18029-008 | 19 | VA | 1999 | Jun | Henrico | *R. catesbeiana* | 46 | NA | NO | NA | NA | NA | Chytridiomycosis | NA |
| 28 | 1999-179 | 18029-006 | 19 | VA | 1999 | Jun | Henrico | *R. catesbeiana* | 35 | NA | NO | NA | NA | NA | Chytridiomycosis | NA |
| 28 | 1999-179 | 18029-004 | 19 | VA | 1999 | Jun | Henrico | *R. catesbeiana* | 41 | NA | NO | NA | NA | NA | Chytridiomycosis | NA |
| 28 | 1999-179 | 18029-003 | 19 | VA | 1999 | Jun | Henrico | *R. catesbeiana* | 36 | NA | NO | NA | NA | NA | Chytridiomycosis | NA |
| 28 | 1999-179 | 18029-002 | 19 | VA | 1999 | Jun | Henrico | *R. catesbeiana* | 26 | NA | NO | NA | NA | NA | Chytridiomycosis | NA |
| 28 | 1999-179 | 18029-001 | 19 | VA | 1999 | Jun | Henrico | *R. catesbeiana* | 35 | NA | NO | NA | NA | NA | Chytridiomycosis | NA |
| 29 | 2001-053 | 17272-015 | 15 | VA | 2001 | Jun | Augusta | *R. sylvatica* | 41 | POS | YES | YES | NO | NO | SPI | NA |
| 29 | 2001-053 | 17272-014 | 15 | VA | 2001 | Jun | Augusta | *R. sylvatica* | 42 | NA | NO | YES | NO | NO | SPI | NA |
| 29 | 2001-053 | 17272-013 | 15 | VA | 2001 | Jun | Augusta | *R. sylvatica* | 42 | NA | NO | YES | NO | NO | SPI | NA |
| 29 | 2001-053 | 17272-012 | 15 | VA | 2001 | Jun | Augusta | *R. sylvatica* | 41 | NA | NO | NO | NO | YES | SPI | NA |
| 29 | 2001-053 | 17272-011 | 15 | VA | 2001 | Jun | Augusta | *R. sylvatica* | 41 | NA | NO | YES | NO | NO | SPI | NA |
| 29 | 2001-053 | 17272-010 | 15 | VA | 2001 | Jun | Augusta | *R. sylvatica* | 38 | NA | NO | YES | NO | NO | SPI | NA |
| 29 | 2001-053 | 17272-003 | 15 | VA | 2001 | Jun | Augusta | *R. sylvatica* | 37 | NA | NO | YES | NO | NO | SPI | NA |
| 29 | 2001-053 | 17272-002 | 15 | VA | 2001 | Jun | Augusta | *R. sylvatica* | 40 | NA | NO | YES | NO | NO | SPI | NA |
| 29 | 2001-053 | 17272-001 | 15 | VA | 2001 | Jun | Augusta | *R. sylvatica* | 40 | NA | NO | YES | NO | NO | SPI | NA |
| NA | NA | 19323-008 | NA | CA | 2005 | Jan | Trinity | *R. catesbeiana* | 26 | NEG | YES | NA | NA | NA | Und | NA |
| NA | NA | 4963-091 | NA | IA | 2013 | Jul | Story | *R. pipiens* | NA | NEG | YES | NA | NA | NA | Und | NA |
| NA | NA | 4963-070 | NA | IA | 2013 | Jul | Story | *R. pipiens* | 44 | NEG | YES | NA | NA | NA | Und | NA |
| NA | NA | 44614-020 | NA | LA | 2012 | Mar | St. Martin | *R. catesbeiana* | 36 | NEG | YES | NA | NA | NA | Und | NA |
| NA | NA | 44614-019 | NA | LA | 2012 | Mar | St. Martin | *R. catesbeiana* | 35 | NEG | YES | NA | NA | NA | Und | NA |
| NA | NA | 4866-091 | NA | CA | 2003 | Aug | San Diego | *Xenopus laevis* | 46 | NEG | YES | NA | NA | NA | Und | NA |
| NA | NA | 4766-047 | NA | MD | 2002 | Jun | Prince George's | *R. sylvatica* | 41 | NEG | YES | NA | NA | NA | Und | NA |
| NA | NA | 4766-046 | NA | MD | 2002 | Jun | Prince George's | *R. sylvatica* | 42 | NEG | YES | NA | NA | NA | Und | NA |
| NA | NA | 4766-038 | NA | MD | 2002 | Jun | Prince George's | *R. sylvatica* | 40 | NEG | YES | NA | NA | NA | Und | NA |
| NA | NA | 4865-021 | NA | MD | 2004 | Jul | Prince George's | *Rana palustris* | 41 | NEG | YES | NA | NA | NA | Und | NA |
| NA | NA | 44274-008 | NA | MD | 2008 | Jul | Prince George's | *Acris crepitans* | 36 | NEG | YES | NA | NA | NA | Und | NA |
| NA | NA | 44274-007 | NA | MD | 2008 | Jul | Prince George's | *A. crepitans* | 38 | NEG | YES | NA | NA | NA | Und | NA |
| NA | NA | 44274-005 | NA | MD | 2008 | Jul | Prince George's | *R. sphenocephala* | 25 | NEG | YES | NA | NA | NA | Und | NA |
| NA | NA | 44274-004 | NA | MD | 2008 | Jul | Prince George's | *R. sphenocephala* | 25 | POS | YES | NA | NA | NA | Und | NA |
| NA | NA | 44274-002 | NA | MD | 2008 | Jul | Prince George's | *A. crepitans* | 29 | NEG | YES | NA | NA | NA | Und | NA |
| NA | NA | 4893-060 | NA | OR | 2004 | May | Polk | *Pseudacris regilla* | 36 | NEG | YES | NA | NA | NA | Und | NA |
| NA | NA | 4893-059 | NA | OR | 2004 | May | Polk | *P. regilla* | 37 | NEG | YES | NA | NA | NA | Und | NA |
| NA | NA | 4912-159 | NA | SC | 2005 | Jun | Orangeburg | *R. sphenocephala* | 36 | NEG | YES | NA | NA | NA | Und | NA |
| NA | NA | 4912-158 | NA | SC | 2005 | Jun | Orangeburg | *R. sphenocephala* | 38 | NEG | YES | NA | NA | NA | Und | NA |
| NA | 2005-042 | 19423-006 | 25 | FL | 2005 | May | Okaloosa | *R. sphenocephala* | 45 | NEG | YES | NA | NA | NA | Und | NA |
| NA | NA | 4756-014 | NA | CA | 2001 | Jun | Los Angeles | *P. regilla* | 46 | NEG | YES | NA | NA | NA | Und | NA |
| NA | NA | 4866-030 | NA | CA | 2003 | Jul | Los Angeles | *P. regilla* | 46 | NEG | YES | NA | NA | NA | Und | NA |
| NA | NA | 4866-028 | NA | CA | 2003 | Jul | Los Angeles | *P. regilla* | 44 | NEG | YES | NA | NA | NA | Und | NA |
| NA | NA | 4866-021 | NA | CA | 2003 | Jul | Los Angeles | *P. regilla* | 44 | NEG | YES | NA | NA | NA | Und | NA |
| NA | NA | 4866-016 | NA | CA | 2003 | Jul | Los Angeles | *P. regilla* | 46 | NEG | YES | NA | NA | NA | Und | NA |
| NA | NA | 4893-057 | NA | OR | 2004 | May | Linn | *Rana aurora* | 29 | NEG | YES | NA | NA | NA | Und | NA |
| NA | NA | 4893-114 | NA | OR | 2004 | Oct | Lane | *R. catesbeiana* | 36 | NEG | YES | NA | NA | NA | Und | NA |
| NA | NA | 4893-111 | NA | OR | 2004 | Oct | Lane | *R. catesbeiana* | 42 | NEG | YES | NA | NA | NA | Und | NA |
| NA | NA | 4893-106 | NA | OR | 2004 | Oct | Lane | *R. catesbeiana* | 37 | NEG | YES | NA | NA | NA | Und | NA |
| NA | NA | 4893-102 | NA | OR | 2004 | Oct | Lane | *R. catesbeiana* | 37 | NEG | YES | NA | NA | NA | Und | NA |
| NA | NA | 4893-100 | NA | OR | 2004 | Oct | Lane | *R. catesbeiana* | 37 | NEG | YES | NA | NA | NA | Und | NA |
| NA | NA | 4893-099 | NA | OR | 2004 | Oct | Lane | *R. catesbeiana* | 34 | NEG | YES | NA | NA | NA | Und | NA |
| NA | NA | 4893-034 | NA | OR | 2004 | May | Lane | *R. aurora* | 34 | NEG | YES | NA | NA | NA | Und | NA |
| NA | NA | 4893-270 | NA | OR | 2005 | May | Lane | *P. regilla* | 38 | NEG | YES | NA | NA | NA | Und | NA |
| NA | NA | 4824-077 | NA | ME | 2002 | Jun | Hancock | *R. sylvatica* | 39 | NEG | YES | NA | NA | NA | Und | NA |
| NA | NA | 4824-069 | NA | ME | 2002 | Jun | Hancock | *R. sylvatica* | 35 | NEG | YES | NA | NA | NA | Und | NA |
| NA | NA | 4963-077 | NA | IA | 2013 | Jul | Hamilton | *R. pipiens* | 45 | NEG | YES | NA | NA | NA | Und | NA |
| NA | NA | 44617-007 | NA | FL | 2011 | Aug | Clay | *R. catesbeiana* | 29 | NEG | YES | NA | NA | NA | Und | NA |
| NA | NA | 44617-004 | NA | FL | 2011 | Aug | Clay | *R. catesbeiana* | 40 | NEG | YES | NA | NA | NA | Und | NA |
| NA | NA | 44617-003 | NA | FL | 2011 | Aug | Clay | *R. catesbeiana* | 31 | NEG | YES | NA | NA | NA | Und | NA |
| NA | NA | 4772-007 | NA | WA | 2001 | Jul | Clallam | *Rana cascadae* | 35 | NEG | YES | NA | NA | NA | Und | NA |
| NA | NA | 4772-006 | NA | WA | 2001 | Jul | Clallam | *R. cascadae* | 34 | NEG | YES | NA | NA | NA | Und | NA |
| NA | NA | 4963-082 | NA | IA | 2013 | Jul | Boone | *R. pipiens* | 45 | NEG | YES | NA | NA | NA | Und | NA |
| NA | NA | 4823-082 | NA | WI | 2002 | Oct | NA | *R. clamitans* | NA | NEG | YES | NA | NA | NA | Und | NA |
| NA | NA | 4823-079 | NA | WI | 2002 | Oct | NA | *R. clamitans* | 46 | NEG | YES | NA | NA | NA | Und | NA |
| NA | NA | 4823-075 | NA | WI | 2002 | Oct | NA | *R. clamitans* | 36 | NEG | YES | NA | NA | NA | Und | NA |
| NA | NA | 4823-073 | NA | WI | 2002 | Oct | NA | *R. clamitans* | 35 | NEG | YES | NA | NA | NA | Und | NA |
| NA | NA | 4823-067 | NA | WI | 2002 | Oct | NA | *R. catesbeiana* | NA | NEG | YES | NA | NA | NA | Und | NA |
| NA | NA | 4823-066 | NA | WI | 2002 | Oct | NA | *R. catesbeiana* | 46 | NEG | YES | NA | NA | NA | Und | NA |
| NA | NA | 4823-062 | NA | WI | 2002 | Oct | NA | *R. catesbeiana* | 32 | NEG | YES | NA | NA | NA | Und | NA |
| NA | NA | 4823-061 | NA | WI | 2002 | Oct | NA | *R. catesbeiana* | 32 | NEG | YES | NA | NA | NA | Und | NA |
| NA | NA | 18216-007 | NA | AK | 2002 | Jun | NA | *R. sylvatica* | NA | NEG | YES | NA | NA | NA | Und | NA |

| **Table S2:** Anuran mortality event data 1999-2015, investigated by the U.S. Geological Survey-National Wildlife Health Center (NWHC), and the U.S. Geological Survey Amphibian Research and Monitoring Initiative (ARMI).  ^a^Mortality involving tadpole stages; ^b^Mortality involving adult stages; ^c^NWHC epizootic database identification number. | | | | | | | | |
| --- | --- | --- | --- | --- | --- | --- | --- | --- |
| **Event Year** | **Larval stages^a^** | **Adult stages^b^** | **Epizoo Event #^c^** | **Species** | **County or Parish** | **State** | **Diagnosis** | **Etiology** |
| 1999 | YES | NO | 1999-011 | *Rana sylvatica* | Nelson | ND | Viral infection | Ranavirus |
| 1999 | YES | NO | 1999-024 | *Rana catesbeiana* | Lucas | OH | Parasitism | *Lernaea sp.* |
| 1999 | NO | YES | 1999-049 | *R. catesbeiana* | Williamson | IL | Viral infection | Ranavirus |
| 1999 | YES | NO | 1999-050 | *Rana clamitans* | Crow Wing | MN | Viral infection | Ranavirus |
| 1999 | YES | NO | 1999-050 | *Rana septentrionalis* | Crow Wing | MN | Viral infection | Ranavirus |
| 1999 | YES | NO | 1999-050 | *Rana pipiens* | Crow Wing | MN | Viral infection | Ranavirus |
| 1999 | YES | NO | 1999-050 | Unidentified | Crow Wing | MN | Viral infection | Ranavirus |
| 1999 | YES | NO | 1999-053 | *Bufo boreas* | Clear Creek | CO | Fungal infection | *Batrachochytrium dendrobatidis* |
| 1999 | YES | NO | 1999-054 | *R. catesbeiana* | Mendocino | CA | Fungal infection | *B. dendrobatidis* |
| 1999 | YES | NO | 1999-054 | *R. catesbeiana* | Lake | CA | Fungal infection | *B. dendrobatidis* |
| 1999 | YES | NO | 1999-054 | *R. catesbeiana* | Fresno | CA | Fungal infection | *B. dendrobatidis* |
| 1999 | YES | NO | 1999-054 | *R. catesbeiana* | Tuolumne | CA | Fungal infection | *B. dendrobatidis* |
| 1999 | YES | NO | 1999-054 | *Rana draytonii* | Mendocino | CA | Fungal infection | *B. dendrobatidis* |
| 1999 | YES | NO | 1999-054 | *R. draytonii* | Lake | CA | Fungal infection | *B. dendrobatidis* |
| 1999 | YES | NO | 1999-054 | *R. draytonii* | Fresno | CA | Fungal infection | *B. dendrobatidis* |
| 1999 | YES | NO | 1999-054 | *R. draytonii* | Tuolumne | CA | Fungal infection | *B. dendrobatidis* |
| 1999 | YES | NO | 1999-054 | *Rana muscosa* | Mendocino | CA | Fungal infection | *B. dendrobatidis* |
| 1999 | YES | NO | 1999-054 | *R. muscosa* | Lake | CA | Fungal infection | *B. dendrobatidis* |
| 1999 | YES | NO | 1999-054 | *R. muscosa* | Fresno | CA | Fungal infection | *B. dendrobatidis* |
| 1999 | YES | NO | 1999-054 | *R. muscosa* | Tuolumne | CA | Fungal infection | *B. dendrobatidis* |
| 1999 | YES | NO | 1999-054 | *Spea hammondii* | Mendocino | CA | Fungal infection | *B. dendrobatidis* |
| 1999 | YES | NO | 1999-054 | *S. hammondii* | Lake | CA | Fungal infection | *B. dendrobatidis* |
| 1999 | YES | NO | 1999-054 | *S. hammondii* | Fresno | CA | Fungal infection | *B. dendrobatidis* |
| 1999 | YES | NO | 1999-054 | *S. hammondii* | Tuolumne | CA | Fungal infection | *B. dendrobatidis* |
| 1999 | YES | NO | 1999-054 | *Bufo canorus* | Mendocino | CA | Fungal infection | *B. dendrobatidis* |
| 1999 | YES | NO | 1999-054 | *B. canorus* | Lake | CA | Fungal infection | *B. dendrobatidis* |
| 1999 | YES | NO | 1999-054 | *B. canorus* | Fresno | CA | Fungal infection | *B. dendrobatidis* |
| 1999 | YES | NO | 1999-054 | *B. canorus* | Tuolumne | CA | Fungal infection | *B. dendrobatidis* |
| 1999 | YES | NO | 1999-054 | *Pseudacris regilla* | Mendocino | CA | Fungal infection | *B. dendrobatidis* |
| 1999 | YES | NO | 1999-054 | *P. regilla* | Lake | CA | Fungal infection | *B. dendrobatidis* |
| 1999 | YES | NO | 1999-054 | *P. regilla* | Fresno | CA | Fungal infection | *B. dendrobatidis* |
| 1999 | YES | NO | 1999-054 | *P. regilla* | Tuolumne | CA | Fungal infection | *B. dendrobatidis* |
| 1999 | YES | NO | 1999-058 | *R. sylvatica* | Aroostook | ME | Viral infection | Ranavirus |
| 1999 | YES | NO | 1999-060 | *R. septentrionalis* | Aroostook | ME | Undetermined | NA |
| 1999 | YES | NO | 1999-070 | *R. sylvatica* | Aroostook | ME | Viral infection | Ranavirus |
| 1999 | NO | YES | 1999-086 | *R. pipiens* | Boulder | CO | Undetermined | NA |
| 1999 | NO | YES | 1999-090 | *Rana palustris* | Blount | TN | Viral infection | Ranavirus |
| 1999 | YES | NO | 1999-098 | *R. catesbeiana* | Barnstable | MA | Viral infection | Ranavirus |
| 1999 | YES | NO | 1999-099 | *R. clamitans* | Strafford | NH | Viral infection | Ranavirus |
| 1999 | YES | NO | 1999-099 | *R. palustris* | Strafford | NH | Viral infection | Ranavirus |
| 1999 | YES | NO | 1999-110 | *R. catesbeiana* | Carroll | NH | Parasitism | Perkinsea protozoa |
| 1999 | NO | YES | 1999-123 | *R. muscosa* | Fresno | CA | Undetermined | NA |
| 1999 | YES | NO | 1999-130 | *R. pipiens* | McLean | ND | Fungal infection | *B. dendrobatidis* |
| 1999 | YES | NO | 1999-132 | *R. catesbeiana* | Porter | IN | Undetermined | NA |
| 1999 | NA | NA | 1999-133 | *Bufo woodhousii* | Mesa | CO | Undetermined | NA |
| 1999 | NO | YES | 1999-134 | *Rana cascadae* | Jefferson | WA | Undetermined | NA |
| 1999 | YES | NO | 1999-179 | *R. catesbeiana* | Richmond | VA | Parasitism | Perkinsea protozoa |
| 1999 | YES | NO | 1999-179 | *R. catesbeiana* | Richmond | VA | Fungal infection | *B. dendrobatidis* |
| 1999 | YES | NO | 1999-179 | *R. catesbeiana* | Henrico | VA | Fungal infection | *B. dendrobatidis* |
| 2000 | YES | NO | 2000-026 | *B. boreas* | Deschutes | OR | Parasitism | Protozoan, not otherwise specified |
| 2000 | YES | NO | 2000-026 | *P. regilla* | Deschutes | OR | Parasitism | Protozoan, not otherwise specified |
| 2000 | YES | NO | 2000-026 | *B. boreas* | Deschutes | OR | Fungal Infection | *Saprolegnia sp.* |
| 2000 | YES | NO | 2000-026 | *P. regilla* | Deschutes | OR | Fungal Infection | *Saprolegnia sp.* |
| 2000 | YES | NO | 2000-036 | *Rana aurora* | Humboldt | CA | Fungal infection | *B. dendrobatidis* |
| 2000 | YES | NO | 2000-044 | *R. sylvatica* | Graham | NC | Viral infection | Ranavirus |
| 2000 | YES | NO | 2000-048 | *R. septentrionalis* | Crow Wing | MN | Viral infection | Ranavirus |
| 2000 | NO | YES | 2000-053 | *R. pipiens* | Winona | MN | Fungal infection | *B. dendrobatidis* |
| 2000 | NO | YES | 2000-053 | *R. palustris* | Winona | MN | Fungal infection | *B. dendrobatidis* |
| 2000 | NO | YES | 2000-053 | *R. pipiens* | Winona | MN | Undetermined | NA |
| 2000 | NO | YES | 2000-053 | *R. palustris* | Winona | MN | Undetermined | NA |
| 2000 | YES | NO | 2000-054 | Unidentified | Blount | TN | Viral infection | Ranavirus |
| 2000 | YES | NO | 2000-054 | *R. sylvatica* | Blount | TN | Viral infection | Ranavirus |
| 2000 | YES | NO | 2000-067 | *R. catesbeiana* | Graham | NC | Fungal infection | *B. dendrobatidis* |
| 2000 | YES | NO | 2000-067 | *R. catesbeiana* | Graham | NC | Viral infection | Ranavirus |
| 2000 | YES | NO | 2000-068 | *R. catesbeiana* | Hancock | ME | Viral infection | Ranavirus |
| 2000 | YES | NO | 2000-068 | *R. clamitans* | Hancock | ME | Viral infection | Ranavirus |
| 2000 | YES | NO | 2000-068 | *Pseudacris crucifer* | Hancock | ME | Viral infection | Ranavirus |
| 2000 | YES | NO | 2000-070 | *R. catesbeiana* | Marin | CA | Fungal infection | *B. dendrobatidis* |
| 2000 | YES | NO | 2000-070 | *R. draytonii* | Marin | CA | Fungal infection | *B. dendrobatidis* |
| 2000 | YES | NO | 2000-070 | *Rana boylii* | Marin | CA | Fungal infection | *B. dendrobatidis* |
| 2000 | YES | NO | 2000-070 | *B. boreas* | Marin | CA | Fungal infection | *B. dendrobatidis* |
| 2000 | YES | NO | 2000-070 | *P. regilla* | Marin | CA | Fungal infection | *B. dendrobatidis* |
| 2000 | YES | NO | 2000-070 | *R. catesbeiana* | Marin | CA | Undetermined | NA |
| 2000 | YES | NO | 2000-070 | *R. draytonii* | Marin | CA | Undetermined | NA |
| 2000 | YES | NO | 2000-070 | *R. boylii* | Marin | CA | Undetermined | NA |
| 2000 | YES | NO | 2000-070 | *B. boreas* | Marin | CA | Undetermined | NA |
| 2000 | YES | NO | 2000-070 | *P. regilla* | Marin | CA | Undetermined | NA |
| 2000 | YES | NO | 2000-073 | *R. clamitans* | Crow Wing | MN | Parasitism | Perkinsea protozoa |
| 2000 | YES | NO | 2000-073 | *R. septentrionalis* | Crow Wing | MN | Parasitism | Perkinsea protozoa |
| 2000 | YES | NO | 2000-073 | *R. pipiens* | Crow Wing | MN | Parasitism | Perkinsea protozoa |
| 2000 | YES | NO | 2000-073 | *R. clamitans* | Crow Wing | MN | Viral infection | Ranavirus |
| 2000 | YES | NO | 2000-073 | *R. septentrionalis* | Crow Wing | MN | Viral infection | Ranavirus |
| 2000 | YES | NO | 2000-073 | *R. pipiens* | Crow Wing | MN | Viral infection | Ranavirus |
| 2000 | YES | NO | 2000-074 | *R. clamitans* | Hampshire | MA | Viral infection | Ranavirus |
| 2000 | YES | NO | 2000-074 | *R. sylvatica* | Hampshire | MA | Viral infection | Ranavirus |
| 2000 | YES | NO | 2000-074 | *Bufo americanus* | Hampshire | MA | Viral infection | Ranavirus |
| 2000 | NO | YES | 2000-084 | *B. boreas* | Larimer | CO | Fungal infection | *B. dendrobatidis* |
| 2000 | YES | NO | 2000-085 | *R. catesbeiana* | Shasta | CA | Toxicosis | glyphosate |
| 2000 | YES | NO | 2000-093 | *R. clamitans* | York | ME | Trauma | NA |
| 2000 | YES | NO | 2000-098 | *R. catesbeiana* | Penobscot | ME | Viral infection | Ranavirus |
| 2000 | YES | NO | 2000-098 | *R. clamitans* | Penobscot | ME | Viral infection | Ranavirus |
| 2000 | YES | NO | 2000-103 | *Rana sphenocephala* | Onslow | NC | Fungal infection | *B. dendrobatidis* |
| 2000 | YES | NO | 2000-103 | *Hyla gratiosa* | Onslow | NC | Fungal infection | *B. dendrobatidis* |
| 2000 | YES | NO | 2000-104 | *R. catesbeiana* | Oxford | ME | Undetermined | NA |
| 2000 | YES | NO | 2000-104 | *B. americanus* | Oxford | ME | Undetermined | NA |
| 2000 | YES | NO | 2000-107 | *R. catesbeiana* | Cheshire | NH | Viral infection | Ranavirus |
| 2000 | YES | NO | 2000-119 | *R. muscosa* | Mono | CA | Fungal infection | *B. dendrobatidis* |
| 2000 | NO | YES | 2000-121 | *B. boreas* | Teton | WY | Fungal infection | *B. dendrobatidis* |
| 2000 | NO | YES | 2000-123 | *Bufo baxteri* | Carbon | WY | Fungal infection | *B. dendrobatidis* |
| 2000 | NO | YES | 2000-123 | *B. baxteri* | Laramie | WY | Fungal infection | *B. dendrobatidis* |
| 2000 | YES | NO | 2000-128 | Unidentified | Santa Cruz | CA | Fungal infection | *B. dendrobatidis* |
| 2000 | YES | NO | 2000-128 | Unidentified | Santa Cruz | CA | Parasitism | *Echinostoma sp.* |
| 2000 | NO | YES | 2000-199 | *R. muscosa* | Fresno | CA | Fungal infection | *B. dendrobatidis* |
| 2000 | NO | YES | 2000-200 | *R. sylvatica* | Stone | AR | Trauma | predation or aggression |
| 2001 | NO | YES | 2001-016 | *R. sylvatica* | Blount | TN | Trauma | NA |
| 2001 | NO | YES | 2001-016 | *Pseudacris cadaverina* | Blount | TN | Trauma | NA |
| 2001 | NO | YES | 2001-016 | *R. sylvatica* | Blount | TN | Trauma | predation or aggression |
| 2001 | NO | YES | 2001-016 | *P. cadaverina* | Blount | TN | Trauma | predation or aggression |
| 2001 | NO | YES | 2001-016 | *R. sylvatica* | Blount | TN | Emaciation | NA |
| 2001 | NO | YES | 2001-016 | *P. cadaverina* | Blount | TN | Emaciation | NA |
| 2001 | YES | NO | 2001-032 | *S. hammondii* | Orange | CA | Undetermined | NA |
| 2001 | YES | NO | 2001-032 | *P. regilla* | Orange | CA | Undetermined | NA |
| 2001 | YES | NO | 2001-035 | *R. catesbeiana* | Barnstable | MA | Undetermined | NA |
| 2001 | YES | NO | 2001-044 | *P. cadaverina* | San Diego | CA | Parasitism | Trematode, not otherwise specified |
| 2001 | YES | NO | 2001-044 | *P. regilla* | San Diego | CA | Parasitism | Trematode, not otherwise specified |
| 2001 | YES | NO | 2001-053 | *R. sylvatica* | Augusta | VA | Parasitism | Perkinsea protozoa |
| 2001 | YES | NO | 2001-054 | *R. catesbeiana* | Hancock | ME | Viral infection | Ranavirus |
| 2001 | YES | NO | 2001-054 | *R. clamitans* | Hancock | ME | Viral infection | Ranavirus |
| 2001 | YES | NO | 2001-054 | *P. crucifer* | Hancock | ME | Viral infection | Ranavirus |
| 2001 | YES | NO | 2001-071 | *R. sylvatica* | Washington | RI | Viral infection | Ranavirus |
| 2001 | YES | NO | 2001-090 | *R. pipiens* | Stutsman | ND | Undetermined | NA |
| 2001 | YES | NO | 2001-095 | *P. cadaverina* | Los Angeles | CA | Undetermined | NA |
| 2001 | YES | YES | 2001-099 | *R. muscosa* | Fresno | CA | Viral infection | Ranavirus |
| 2001 | YES | NO | 2001-107 | *R. aurora* | Del Norte | CA | Undetermined | NA |
| 2001 | YES | NO | 2001-107 | *P. regilla* | Del Norte | CA | Undetermined | NA |
| 2001 | YES | NO | 2001-108 | *R. sphenocephala* | Harrison | MS | Parasitism | Perkinsea protozoa |
| 2001 | NO | YES | 2001-110 | *Rana luteiventris* | Gallatin | MT | Viral infection | Ranavirus |
| 2001 | NO | YES | 2001-110 | *B. boreas* | Gallatin | MT | Viral infection | Ranavirus |
| 2001 | NO | YES | 2001-110 | *R. luteiventris* | Gallatin | MT | Parasitism | *Echinostoma sp.* |
| 2001 | NO | YES | 2001-110 | *B. boreas* | Gallatin | MT | Parasitism | *Echinostoma sp.* |
| 2001 | NO | YES | 2001-116 | *P. cadaverina* | Riverside | CA | Fungal infection | *B. dendrobatidis* |
| 2001 | NO | YES | 2001-116 | *P. cadaverina* | Los Angeles | CA | Fungal infection | *B. dendrobatidis* |
| 2001 | NO | YES | 2001-116 | *P. regilla* | Riverside | CA | Fungal infection | *B. dendrobatidis* |
| 2001 | NO | YES | 2001-116 | *P. regilla* | Los Angeles | CA | Fungal infection | *B. dendrobatidis* |
| 2001 | NA | NA | 2001-118 | Unidentified | Glenn | CA | Natural toxin | *Clostridium botulinum*, type C |
| 2001 | NO | YES | 2001-126 | *R. sphenocephala* | Alachua | FL | Bacterial Infection | NA |
| 2001 | NO | YES | 2001-126 | *Hyla cinerea* | Alachua | FL | Bacterial Infection | NA |
| 2001 | YES | NO | 2001-133 | *Xenopus laevis* | San Diego | CA | Emaciation | NA |
| 2001 | YES | NO | 2001-134 | *R. sylvatica* | Fairbanks North Star | AK | Viral infection | Ranavirus |
| 2001 | NO | YES | 2001-135 | *B. boreas* | Garfield | UT | Fungal infection | *B. dendrobatidis* |
| 2001 | NO | YES | 2001-136 | *R. luteiventris* | Wasatch | UT | Fungal infection | *B. dendrobatidis* |
| 2001 | NO | YES | 2001-137 | *R. muscosa* | Fresno | CA | Fungal infection | *B. dendrobatidis* |
| 2001 | NO | YES | 2001-137 | *P. regilla* | Fresno | CA | Fungal infection | *B. dendrobatidis* |
| 2001 | YES | NO | 2001-149 | *R. sphenocephala* | Mississippi | AR | Undetermined | NA |
| 2001 | NO | YES | 2001-156 | *R. luteiventris* | Ravalli | MT | Exposure | hypothermia, frostbite |
| 2001 | YES | NO | 2001-157 | *B. boreas* | Ravalli | MT | Viral infection | Ranavirus |
| 2001 | NO | YES | 2001-167 | *R. sylvatica* | Stone | AR | Trauma | predation or aggression |
| 2001 | NO | YES | 2001-167 | *R. sylvatica* | Stone | AR | Exposure | hyperthermia |
| 2001 | NO | YES | 2001-179 | *R. pipiens* | Washoe | NV | Undetermined | NA |
| 2001 | NO | YES | 2001-180 | *R. catesbeiana* | Washoe | NV | Undetermined | NA |
| 2002 | NO | YES | 2002-008 | *R. sylvatica* | Stone | AR | Trauma | predation or aggression |
| 2002 | NO | YES | 2002-008 | *R. sylvatica* | Stone | AR | Exposure | hypothermia, frostbite |
| 2002 | NO | YES | 2002-028 | *Osteophilus septentrionalis* | Monroe | FL | Viral infection | Ranavirus |
| 2002 | NO | YES | 2002-028 | *H. cinerea* | Monroe | FL | Viral infection | Ranavirus |
| 2002 | YES | NO | 2002-031 | *Rana berlandieri* | Travis | TX | Undetermined | NA |
| 2002 | NO | YES | 2002-033 | *R. pipiens* | Grand Isle | VT | Trauma | predation or aggression |
| 2002 | NO | YES | 2002-033 | *R. pipiens* | Grand Isle | VT | Undetermined | NA |
| 2002 | YES | NO | 2002-035 | *R. catesbeiana* | Liberty | GA | Fungal infection | *B. dendrobatidis* |
| 2002 | YES | NO | 2002-036 | *R. catesbeiana* | Onslow | NC | Fungal infection | *B. dendrobatidis* |
| 2002 | YES | NO | 2002-042 | *R. sphenocephala* | Avoyelles | LA | Emaciation | starvation |
| 2002 | YES | NO | 2002-050 | *R. sylvatica* | Washington | RI | Viral infection | Ranavirus |
| 2002 | YES | YES | 2002-055 | *R. catesbeiana* | Oxford | ME | Trauma | predation or aggression |
| 2002 | YES | YES | 2002-055 | *R. catesbeiana* | Oxford | ME | Fungal infection | *B. dendrobatidis* |
| 2002 | YES | NO | 2002-063 | *R. clamitans* | Otter Tail | MN | Undetermined | NA |
| 2002 | YES | YES | 2002-096 | *R. catesbeiana* | Waldo | ME | Parasitism | *Echinostoma sp.* |
| 2002 | YES | YES | 2002-096 | *R. clamitans* | Waldo | ME | Parasitism | *Echinostoma sp.* |
| 2002 | YES | NO | 2002-098 | *R. catesbeiana* | Lake | IN | Viral infection | Ranavirus |
| 2002 | NO | YES | 2002-128 | *R. catesbeiana* | Washoe | NV | Undetermined | NA |
| 2002 | NO | YES | 2002-129 | *R. boylii* | Sonoma | CA | Undetermined | NA |
| 2002 | NO | YES | 2002-130 | *Rana chiricahuensis* | Catron | NM | Fungal infection | *B. dendrobatidis* |
| 2002 | NO | YES | 2002-135 | *R. sylvatica* | Graham | NC | Viral infection | Ranavirus |
| 2002 | YES | NO | 2002-149 | *R. sphenocephala* | Wakulla | FL | Parasitism | Perkinsea protozoa |
| 2002 | NO | YES | 2002-176 | *Scaphiopus holbrookii* | Brevard | FL | Undetermined | NA |
| 2002 | NO | YES | 2002-176 | *Hyla squirella* | Brevard | FL | Undetermined | NA |
| 2002 | NO | YES | 2002-184 | *R. luteiventris* | Wasatch | UT | Undetermined | NA |
| 2002 | NO | YES | 2002-184 | *P. cadaverina* | Wasatch | UT | Undetermined | NA |
| 2002 | NO | YES | 2002-191 | *R. catesbeiana* | Benton | OR | Viral infection | Ranavirus |
| 2002 | NO | YES | 2002-191 | *R. catesbeiana* | Douglas | OR | Viral infection | Ranavirus |
| 2002 | NO | YES | 2002-191 | *R. catesbeiana* | Lane | OR | Viral infection | Ranavirus |
| 2002 | YES | NO | 2002-191 | *R. aurora* | Benton | OR | Viral infection | Ranavirus |
| 2002 | YES | NO | 2002-191 | *R. aurora* | Douglas | OR | Viral infection | Ranavirus |
| 2002 | YES | NO | 2002-191 | *R. aurora* | Lane | OR | Viral infection | Ranavirus |
| 2002 | YES | NO | 2002-191 | *P. regilla* | Benton | OR | Viral infection | Ranavirus |
| 2002 | YES | NO | 2002-191 | *P. regilla* | Douglas | OR | Viral infection | Ranavirus |
| 2002 | YES | NO | 2002-191 | *P. regilla* | Lane | OR | Viral infection | Ranavirus |
| 2002 | YES | NO | 2002-192 | *R. catesbeiana* | Marion | IA | Fungal Infection | *Saprolegnia sp.* |
| 2003 | YES | NO | 2003-020 | *B. americanus* | Blount | TN | Undetermined | NA |
| 2003 | YES | NO | 2003-021 | *Rana sevosa* | Harrison | MS | Parasitism | Perkinsea protozoa |
| 2003 | YES | NO | 2003-021 | *R. sphenocephala* | Harrison | MS | Parasitism | Perkinsea protozoa |
| 2003 | YES | NO | 2003-034 | *R. sphenocephala* | St. Tammany | LA | Undetermined | NA |
| 2003 | YES | NO | 2003-034 | *R. sphenocephala* | St. Tammany | LA | Parasitism | Perkinsea protozoa |
| 2003 | YES | NO | 2003-057 | Unidentified | Blount | TN | Undetermined | NA |
| 2003 | YES | NO | 2003-057 | *B. americanus* | Blount | TN | Undetermined | NA |
| 2003 | YES | NO | 2003-068 | *R. palustris* | Prince Georges | MD | Parasitism | Perkinsea protozoa |
| 2003 | YES | NO | 2003-068 | *R. sphenocephala* | Prince Georges | MD | Parasitism | Perkinsea protozoa |
| 2003 | NO | YES | 2003-090 | *R. clamitans* | Prince Georges | MD | Undetermined | NA |
| 2003 | NO | YES | 2003-097 | *O. septentrionalis* | Monroe | FL | Undetermined | NA |
| 2003 | NO | YES | 2003-097 | *H. cinerea* | Monroe | FL | Undetermined | NA |
| 2003 | YES | YES | 2003-099 | *R. catesbeiana* | Aroostook | ME | Viral infection | Ranavirus |
| 2003 | YES | YES | 2003-099 | *R. clamitans* | Aroostook | ME | Viral infection | Ranavirus |
| 2003 | NO | YES | 2003-115 | *R. luteiventris* | Teton | WY | Parasitism | *Diplostomulum sp.* |
| 2003 | YES | NO | 2003-139 | *R. sphenocephala* | Virginia Beach | VA | Viral infection | Ranavirus |
| 2003 | YES | NO | 2003-144 | *R. sevosa* | Harrison | MS | Undetermined | NA |
| 2003 | YES | NO | 2003-144 | *Acris gryllus* | Harrison | MS | Undetermined | NA |
| 2003 | YES | NO | 2003-144 | *H. squirella* | Harrison | MS | Undetermined | NA |
| 2003 | NO | YES | 2003-167 | *R. luteiventris* | Wasatch | UT | Undetermined | NA |
| 2003 | YES | NO | 2003-178 | *Spea multiplicata* | Briscoe | TX | Parasitism | *Hannemania sp.* |
| 2003 | YES | NO | 2003-178 | *S. multiplicata* | Floyd | TX | Parasitism | *Hannemania sp.* |
| 2003 | YES | NO | 2003-178 | *Spea bombifrons* | Briscoe | TX | Parasitism | *Hannemania sp.* |
| 2003 | YES | NO | 2003-178 | *S. bombifrons* | Floyd | TX | Parasitism | *Hannemania sp.* |
| 2004 | YES | NO | 2004-011 | *R. catesbeiana* | Harrison | MS | Parasitism | Perkinsea protozoa |
| 2004 | YES | NO | 2004-011 | *R. sphenocephala* | Harrison | MS | Parasitism | Perkinsea protozoa |
| 2004 | NO | YES | 2004-022 | *P. crucifer* | Anderson | TN | Toxicosis | heavy metal, not otherwise specified |
| 2004 | NO | YES | 2004-022 | *P. cadaverina* | Anderson | TN | Toxicosis | heavy metal, not otherwise specified |
| 2004 | YES | NO | 2004-034 | *R. clamitans* | Rockcastle | KY | Viral infection | Ranavirus |
| 2004 | YES | NO | 2004-034 | *R. sylvatica* | Rockcastle | KY | Viral infection | Ranavirus |
| 2004 | YES | NO | 2004-036 | *R. chiricahuensis* | Sierra | NM | Viral infection | Ranavirus |
| 2004 | YES | NO | 2004-041 | *R. sphenocephala* | Morgan | AL | Parasitism | Trematode, not otherwise specified |
| 2004 | YES | NO | 2004-048 | *R. sylvatica* | Isanti | MN | Viral infection | Ranavirus |
| 2004 | YES | NO | 2004-048 | *R. sylvatica* | Isanti | MN | Parasitism | NA |
| 2004 | YES | NO | 2004-054 | *R. catesbeiana* | Modoc | CA | Parasitism | *Echinostoma sp.* |
| 2004 | YES | NO | 2004-059 | *Hyla arenicolor* | Santa Cruz | AZ | Parasitism | NA |
| 2004 | NO | YES | 2004-063 | *R. pipiens* | Newport | RI | Viral infection | Ranavirus |
| 2004 | YES | NO | 2004-073 | *R. sylvatica* | Kenai Peninsula | AK | Parasitism | Perkinsea protozoa |
| 2004 | YES | NO | 2004-074 | *R. sylvatica* | Bethel | AK | Parasitism | Perkinsea protozoa |
| 2004 | YES | NO | 2004-083 | *R. catesbeiana* | Vilas | WI | Undetermined | NA |
| 2004 | NO | YES | 2004-108 | *R. luteiventris* | Teton | WY | Viral infection | Ranavirus |
| 2004 | NO | YES | 2004-111 | *R. catesbeiana* | Grant | WA | Undetermined | NA |
| 2004 | NO | YES | 2004-126 | *R. pipiens* | Dane | WI | Fungal infection | *B. dendrobatidis* |
| 2004 | NO | YES | 2004-131 | *R. chiricahuensis* | Grant | NM | Fungal infection | *B. dendrobatidis* |
| 2004 | NO | YES | 2004-132 | *B. boreas* | Glacier | MT | Fungal infection | *B. dendrobatidis* |
| 2004 | NO | YES | 2004-142 | *B. boreas* | Baker | OR | Fungal infection | *B. dendrobatidis* |
| 2005 | YES | NO | 2005-016 | *R. catesbeiana* | Hernando | FL | Parasitism | Perkinsea protozoa |
| 2005 | YES | NO | 2005-016 | *Rana capito* | Hernando | FL | Parasitism | Perkinsea protozoa |
| 2005 | YES | NO | 2005-016 | *R. sphenocephala* | Hernando | FL | Parasitism | Perkinsea protozoa |
| 2005 | NO | YES | 2005-022 | *R. chiricahuensis* | Santa Cruz | AZ | Fungal infection | *B. dendrobatidis* |
| 2005 | YES | NO | 2005-028 | *R. catesbeiana* | Putnam | FL | Parasitism | Perkinsea protozoa |
| 2005 | YES | NO | 2005-028 | *R. capito* | Putnam | FL | Parasitism | Perkinsea protozoa |
| 2005 | YES | NO | 2005-028 | *R. sphenocephala* | Putnam | FL | Parasitism | Perkinsea protozoa |
| 2005 | NO | YES | 2005-033 | *R. luteiventris* | Lake | MT | Trauma | predation or aggression |
| 2005 | YES | NO | 2005-037 | *R. sphenocephala* | Morgan | AL | Parasitism | *Diplostomulum sp.* |
| 2005 | YES | NO | 2005-042 | *R. capito* | Okaloosa | FL | Undetermined | NA |
| 2005 | YES | NO | 2005-042 | *R. sphenocephala* | Okaloosa | FL | Undetermined | NA |
| 2005 | YES | NO | 2005-043 | *Hyla chrysoscelis* | Montgomery | MD | Fungal infection | *B. dendrobatidis* |
| 2005 | YES | NO | 2005-043 | *R. clamitans* | Montgomery | MD | Fungal infection | *B. dendrobatidis* |
| 2005 | YES | NO | 2005-043 | *R. sylvatica* | Montgomery | MD | Fungal infection | *B. dendrobatidis* |
| 2005 | YES | NO | 2005-043 | *H. chrysoscelis* | Montgomery | MD | Viral infection | Ranavirus |
| 2005 | YES | NO | 2005-043 | *R. clamitans* | Montgomery | MD | Viral infection | Ranavirus |
| 2005 | YES | NO | 2005-043 | *R. sylvatica* | Montgomery | MD | Viral infection | Ranavirus |
| 2005 | YES | NO | 2005-043 | *H. chrysoscelis* | Montgomery | MD | Parasitism | NA |
| 2005 | YES | NO | 2005-043 | *R. clamitans* | Montgomery | MD | Parasitism | NA |
| 2005 | YES | NO | 2005-043 | *R. sylvatica* | Montgomery | MD | Parasitism | NA |
| 2005 | YES | NO | 2005-050 | *R. sylvatica* | Kenai Peninsula | AK | Parasitism | Perkinsea protozoa |
| 2005 | NO | YES | 2005-051 | *R. sphenocephala* | St. Tammany | LA | Undetermined | NA |
| 2005 | YES | NO | 2005-052 | *R. sylvatica* | Washington | RI | Viral infection | Ranavirus |
| 2005 | YES | NO | 2005-052 | *Hyla versicolor* | Washington | RI | Viral infection | Ranavirus |
| 2005 | NO | YES | 2005-055 | *Pseudacris clarkia* | Briscoe | TX | Viral infection | Ranavirus |
| 2005 | YES | NO | 2005-060 | *R. clamitans* | Hancock | ME | Viral infection | Ranavirus |
| 2005 | YES | NO | 2005-060 | *R. sylvatica* | Hancock | ME | Viral infection | Ranavirus |
| 2005 | YES | NO | 2005-089 | *R. pipiens* | Lake | MT | Trauma | NA |
| 2005 | YES | NO | 2005-098 | *R. catesbeiana* | Benton | AR | Fungal infection | *B. dendrobatidis* |
| 2005 | NO | YES | 2005-101 | *R. pipiens* | Glacier | MT | Fungal infection | *B. dendrobatidis* |
| 2005 | YES | NO | 2005-114 | *R. luteiventris* | Teton | WY | Fungal infection | *B. dendrobatidis* |
| 2005 | NO | YES | 2005-124 | *R. catesbeiana* | Crosby | TX | Undetermined | NA |
| 2005 | YES | NO | 2005-138 | *B. boreas* | Park | WY | Viral infection | Ranavirus |
| 2005 | YES | NO | 2005-139 | *R. luteiventris* | Teton | WY | Viral infection | Ranavirus |
| 2006 | YES | NO | 2006-006 | *R. clamitans* | Bay | FL | Viral infection | Ranavirus |
| 2006 | YES | NO | 2006-012 | *Rana heckscheri* | Miller | GA | Parasitism | Perkinsea protozoa |
| 2006 | NO | YES | 2006-015 | *R. catesbeiana* | Monterey | CA | Fungal infection | *B. dendrobatidis* |
| 2006 | NO | YES | 2006-018 | *R. sylvatica* | Hancock | ME | Trauma | predation or aggression |
| 2006 | YES | NO | 2006-023 | *R. clamitans* | Penobscot | ME | Parasitism | *Ichthyophonus sp.* |
| 2006 | YES | NO | 2006-112 | *R. catesbeiana* | Santa Cruz | AZ | Parasitism | *Clinostomum sp.* |
| 2006 | YES | NO | 2006-116 | *P. regilla* | Monterey | CA | Viral infection | Ranavirus |
| 2006 | YES | NO | 2006-123 | *R. catesbeiana* | Essex | NY | Undetermined | NA |
| 2006 | YES | NO | 2006-123 | *R. sylvatica* | Essex | NY | Undetermined | NA |
| 2007 | NO | YES | 2007-047 | *R. pipiens* | Outagamie | WI | Undetermined | NA |
| 2007 | YES | NO | 2007-049 | *R. clamitans* | Washington | RI | Viral infection | Ranavirus |
| 2007 | YES | NO | 2007-049 | *R. sylvatica* | Washington | RI | Viral infection | Ranavirus |
| 2007 | YES | NO | 2007-050 | *R. draytonii* | Santa Cruz | CA | Viral infection | Ranavirus |
| 2007 | YES | NO | 2007-050 | *P. regilla* | Santa Cruz | CA | Viral infection | Ranavirus |
| 2007 | YES | NO | 2007-050 | *R. draytonii* | Santa Cruz | CA | Parasitism | *Echinostoma sp.* |
| 2007 | YES | NO | 2007-050 | *P. regilla* | Santa Cruz | CA | Parasitism | *Echinostoma sp.* |
| 2007 | NO | YES | 2007-081 | *Rana grylio* | Lake | FL | Trauma | predation or aggression |
| 2007 | YES | YES | 2007-113 | *R. luteiventris* | Teton | WY | Viral infection | Ranavirus |
| 2007 | YES | NO | 2007-121 | *B. boreas* | Baker | OR | Fungal infection | *B. dendrobatidis* |
| 2008 | NO | YES | 2008-080 | *Rana pretiosa* | Wasco | OR | Undetermined | NA |
| 2008 | NO | YES | 2008-080 | *P. regilla* | Wasco | OR | Undetermined | NA |
| 2008 | YES | NO | 2008-086 | *R. sylvatica* | Washington | RI | Viral infection | Ranavirus |
| 2008 | YES | NO | 2008-100 | *R. catesbeiana* | Santa Cruz | CA | Fungal infection | *B. dendrobatidis* |
| 2008 | YES | NO | 2008-100 | *R. catesbeiana* | Monterey | CA | Fungal infection | *B. dendrobatidis* |
| 2008 | YES | NO | 2008-100 | *R. draytonii* | Santa Cruz | CA | Fungal infection | *B. dendrobatidis* |
| 2008 | YES | NO | 2008-100 | *R. draytonii* | Monterey | CA | Fungal infection | *B. dendrobatidis* |
| 2008 | NO | YES | 2008-104 | Unidentified | Sumter | SC | Undetermined | NA |
| 2008 | YES | NO | 2008-122 | *Rana blairi* | Clay | NE | Viral infection | Ranavirus |
| 2008 | YES | NO | 2008-122 | *R. blairi* | Fillmore | NE | Viral infection | Ranavirus |
| 2008 | YES | NO | 2008-122 | *R. blairi* | Clay | NE | Parasitism | NA |
| 2008 | YES | NO | 2008-122 | *R. blairi* | Fillmore | NE | Parasitism | NA |
| 2008 | YES | NO | 2008-172 | *B. boreas* | Shasta | CA | Viral infection | Ranavirus |
| 2008 | YES | NO | 2008-183 | *R. catesbeiana* | Marion | OR | Viral infection | Ranavirus |
| 2008 | YES | NO | 2008-216 | *R. clamitans* | Suffolk | NY | Fungal infection | *B. dendrobatidis* |
| 2008 | YES | NO | 2008-216 | *R. sphenocephala* | Suffolk | NY | Fungal infection | *B. dendrobatidis* |
| 2008 | YES | NO | 2008-216 | *R. clamitans* | Suffolk | NY | Parasitism | Perkinsea protozoa |
| 2008 | YES | NO | 2008-216 | *R. sphenocephala* | Suffolk | NY | Parasitism | Perkinsea protozoa |
| 2008 | YES | NO | 2008-220 | *R. catesbeiana* | Suffolk | NY | Fungal infection | *B. dendrobatidis* |
| 2008 | YES | NO | 2008-220 | *R. catesbeiana* | Richmond | NY | Fungal infection | *B. dendrobatidis* |
| 2008 | YES | NO | 2008-220 | *R. sphenocephala* | Suffolk | NY | Fungal infection | *B. dendrobatidis* |
| 2008 | YES | NO | 2008-220 | *R. sphenocephala* | Richmond | NY | Fungal infection | *B. dendrobatidis* |
| 2008 | YES | NO | 2008-220 | *R. catesbeiana* | Suffolk | NY | Parasitism | *Lernaea sp.* |
| 2008 | YES | NO | 2008-220 | *R. catesbeiana* | Richmond | NY | Parasitism | *Lernaea sp.* |
| 2008 | YES | NO | 2008-220 | *R. sphenocephala* | Suffolk | NY | Parasitism | *Lernaea sp.* |
| 2008 | YES | NO | 2008-220 | *R. sphenocephala* | Richmond | NY | Parasitism | *Lernaea sp.* |
| 2008 | YES | NO | 2008-220 | *R. catesbeiana* | Suffolk | NY | Parasitism | Perkinsea protozoa |
| 2008 | YES | NO | 2008-220 | *R. catesbeiana* | Richmond | NY | Parasitism | Perkinsea protozoa |
| 2008 | YES | NO | 2008-220 | *R. sphenocephala* | Suffolk | NY | Parasitism | Perkinsea protozoa |
| 2008 | YES | NO | 2008-220 | *R. sphenocephala* | Richmond | NY | Parasitism | Perkinsea protozoa |
| 2008 | NO | YES | 2008-242 | *P. cadaverina* | San Bernardino | CA | Deformity | NA |
| 2008 | YES | YES | 2008-243 | *Bufo californicus* | San Diego | CA | Trauma | predation or aggression |
| 2008 | YES | YES | 2008-243 | *B. californicus* | San Diego | CA | Undetermined | NA |
| 2009 | YES | NO | 2009-024 | *R. catesbeiana* | Mendocino | CA | Undetermined | NA |
| 2009 | NO | YES | 2009-038 | *R. pipiens* | Washoe | NV | Fungal infection | *B. dendrobatidis* |
| 2009 | NO | YES | 2009-038 | *R. pipiens* | Washoe | NV | Toxicosis | NA |
| 2009 | YES | NO | 2009-075 | *R. catesbeiana* | Suffolk | NY | Fungal infection | *B. dendrobatidis* |
| 2009 | YES | NO | 2009-075 | *R. catesbeiana* | Burlington | NJ | Fungal infection | *B. dendrobatidis* |
| 2009 | YES | NO | 2009-075 | *R. catesbeiana* | Richmond | NY | Fungal infection | *B. dendrobatidis* |
| 2009 | YES | NO | 2009-075 | *R. catesbeiana* | Putnam | NY | Fungal infection | *B. dendrobatidis* |
| 2009 | YES | NO | 2009-075 | *R. sphenocephala* | Suffolk | NY | Fungal infection | *B. dendrobatidis* |
| 2009 | YES | NO | 2009-075 | *R. sphenocephala* | Burlington | NJ | Fungal infection | *B. dendrobatidis* |
| 2009 | YES | NO | 2009-075 | *R. sphenocephala* | Richmond | NY | Fungal infection | *B. dendrobatidis* |
| 2009 | YES | NO | 2009-075 | *R. sphenocephala* | Putnam | NY | Fungal infection | *B. dendrobatidis* |
| 2009 | YES | NO | 2009-075 | *R. catesbeiana* | Suffolk | NY | Parasitism | Perkinsea protozoa |
| 2009 | YES | NO | 2009-075 | *R. catesbeiana* | Burlington | NJ | Parasitism | Perkinsea protozoa |
| 2009 | YES | NO | 2009-075 | *R. catesbeiana* | Richmond | NY | Parasitism | Perkinsea protozoa |
| 2009 | YES | NO | 2009-075 | *R. catesbeiana* | Putnam | NY | Parasitism | Perkinsea protozoa |
| 2009 | YES | NO | 2009-075 | *R. sphenocephala* | Suffolk | NY | Parasitism | Perkinsea protozoa |
| 2009 | YES | NO | 2009-075 | *R. sphenocephala* | Burlington | NJ | Parasitism | Perkinsea protozoa |
| 2009 | YES | NO | 2009-075 | *R. sphenocephala* | Richmond | NY | Parasitism | Perkinsea protozoa |
| 2009 | YES | NO | 2009-075 | *R. sphenocephala* | Putnam | NY | Parasitism | Perkinsea protozoa |
| 2009 | YES | NO | 2009-075 | *R. catesbeiana* | Suffolk | NY | Undetermined | NA |
| 2009 | YES | NO | 2009-075 | *R. catesbeiana* | Burlington | NJ | Undetermined | NA |
| 2009 | YES | NO | 2009-075 | *R. catesbeiana* | Richmond | NY | Undetermined | NA |
| 2009 | YES | NO | 2009-075 | *R. catesbeiana* | Putnam | NY | Undetermined | NA |
| 2009 | YES | NO | 2009-075 | *R. sphenocephala* | Suffolk | NY | Undetermined | NA |
| 2009 | YES | NO | 2009-075 | *R. sphenocephala* | Burlington | NJ | Undetermined | NA |
| 2009 | YES | NO | 2009-075 | *R. sphenocephala* | Richmond | NY | Undetermined | NA |
| 2009 | YES | NO | 2009-075 | *R. sphenocephala* | Putnam | NY | Undetermined | NA |
| 2009 | NO | YES | 2009-081 | *R. pipiens* | Freeborn | MN | Fungal infection | *B. dendrobatidis* |
| 2009 | YES | NO | 2009-082 | *R. sylvatica* | Hampshire | MA | Viral infection | Ranavirus |
| 2009 | YES | NO | 2009-083 | *P. crucifer* | Blount | TN | Viral infection | Ranavirus |
| 2009 | NO | YES | 2009-083 | *R. sylvatica* | Blount | TN | Viral infection | Ranavirus |
| 2009 | YES | NO | 2009-118 | *R. luteiventris* | Latah | ID | Viral infection | Ranavirus |
| 2009 | YES | NO | 2009-118 | *P. regilla* | Latah | ID | Viral infection | Ranavirus |
| 2009 | YES | NO | 2009-129 | *R. catesbeiana* | Vilas | WI | Viral infection | Ranavirus |
| 2009 | YES | NO | 2009-129 | *R. clamitans* | Vilas | WI | Viral infection | Ranavirus |
| 2009 | YES | NO | 2009-129 | *R. pipiens* | Vilas | WI | Viral infection | Ranavirus |
| 2009 | NO | YES | 2009-130 | *R. clamitans* | Baltimore | MD | Fungal infection | *B. dendrobatidis* |
| 2009 | NO | YES | 2009-130 | *B. americanus* | Baltimore | MD | Fungal infection | *B. dendrobatidis* |
| 2009 | YES | NO | 2009-131 | *B. boreas* | Skamania | WA | Fungal Infection | *Saprolegnia sp.* |
| 2009 | YES | NO | 2009-137 | *R. catesbeiana* | Centre | PA | Viral infection | Ranavirus |
| 2009 | YES | NO | 2009-158 | *R. catesbeiana* | Humboldt | NV | Fungal infection | *B. dendrobatidis* |
| 2009 | YES | NO | 2009-210 | *R. catesbeiana* | Queen Anne’s | MD | Trauma | predation or aggression |
| 2009 | YES | NO | 2009-210 | *R. catesbeiana* | Queen Anne’s | MD | Undetermined | NA |
| 2009 | NO | YES | 2009-224 | *R. sylvatica* | New Haven | CT | Viral infection | Ranavirus |
| 2009 | YES | NO | 2009-244 | *B. boreas* | Douglas | NV | Undetermined | NA |
| 2009 | YES | NO | 2009-244 | *P. regilla* | Douglas | NV | Undetermined | NA |
| 2010 | NO | YES | 2010-027 | *R. blairi* | Hamilton | NE | Undetermined | NA |
| 2010 | NO | YES | 2010-027 | *R. blairi* | Adams | NE | Undetermined | NA |
| 2010 | YES | NO | 2010-067 | *R. clamitans* | Talbot | MD | Viral infection | Ranavirus |
| 2010 | YES | NO | 2010-067 | *R. sphenocephala* | Talbot | MD | Viral infection | Ranavirus |
| 2010 | NO | YES | 2010-129 | *R. catesbeiana* | Humboldt | NV | Trauma | predation or aggression |
| 2010 | NO | YES | 2010-129 | *R. catesbeiana* | Humboldt | NV | Undetermined | NA |
| 2010 | YES | NO | 2010-139 | *R. catesbeiana* | Murray | GA | Viral infection | Ranavirus |
| 2010 | NO | YES | 2010-158 | *R. sylvatica* | New Haven | CT | Viral infection | Ranavirus |
| 2010 | YES | NO | 2010-196 | *R. sylvatica* | Montgomery | MD | Viral infection | Ranavirus |
| 2010 | YES | NO | 2010-196 | *R. sylvatica* | Montgomery | MD | Bacterial Infection | NA |
| 2010 | YES | NO | 2010-207 | *R. catesbeiana* | Plumas | CA | Undetermined | NA |
| 2011 | YES | NO | 2011-033 | *R. sphenocephala* | Morris | NJ | Emaciation | starvation |
| 2011 | YES | NO | 2011-091 | *R. sylvatica* | New London | CT | Viral infection | Ranavirus |
| 2011 | YES | NO | 2011-129 | *R. clamitans* | Frederick | MD | Undetermined | NA |
| 2011 | YES | NO | 2011-129 | *R. clamitans* | Frederick | MD | Parasitism | NA |
| 2011 | YES | NO | 2011-136 | *R. sevosa* | Harrison | MS | Undetermined | NA |
| 2011 | NO | YES | 2011-172 | *R. catesbeiana* | Multnomah | OR | Viral infection | Ranavirus |
| 2011 | NO | YES | 2011-187 | *R. clamitans* | Dakota | MN | Fungal infection | *B. dendrobatidis* |
| 2011 | NO | YES | 2011-187 | *R. pipiens* | Dakota | MN | Fungal infection | *B. dendrobatidis* |
| 2011 | NO | YES | 2011-187 | *R. sylvatica* | Dakota | MN | Fungal infection | *B. dendrobatidis* |
| 2011 | NO | YES | 2011-187 | *B. americanus* | Dakota | MN | Fungal infection | *B. dendrobatidis* |
| 2011 | NO | YES | 2011-187 | *R. clamitans* | Dakota | MN | Viral infection | Ranavirus |
| 2011 | NO | YES | 2011-187 | *R. pipiens* | Dakota | MN | Viral infection | Ranavirus |
| 2011 | NO | YES | 2011-187 | *R. sylvatica* | Dakota | MN | Viral infection | Ranavirus |
| 2011 | NO | YES | 2011-187 | *B. americanus* | Dakota | MN | Viral infection | Ranavirus |
| 2011 | YES | NO | 2011-212 | *R. pipiens* | Will | IL | Emaciation | NA |
| 2011 | YES | NO | 2011-222 | *R. catesbeiana* | Washington | OR | Fungal infection | *B. dendrobatidis* |
| 2012 | NO | YES | 2012-059 | *R. pipiens* | McLeod | MN | Fungal infection | *B. dendrobatidis* |
| 2012 | NA | NA | 2012-108 | Unidentified | Santa Clara | CA | Undetermined | NA |
| 2012 | YES | NO | 2012-125 | *R. luteiventris* | Teton | WY | Viral infection | Ranavirus |
| 2012 | YES | NO | 2012-125 | *R. luteiventris* | Park | WY | Viral infection | Ranavirus |
| 2012 | YES | NO | 2012-125 | *B. boreas* | Teton | WY | Viral infection | Ranavirus |
| 2012 | YES | NO | 2012-125 | *B. boreas* | Park | WY | Viral infection | Ranavirus |
| 2012 | YES | NO | 2012-168 | *R. catesbeiana* | Deschutes | OR | Viral infection | Ranavirus |
| 2012 | YES | NO | 2012-168 | *R. pretiosa* | Deschutes | OR | Viral infection | Ranavirus |
| 2012 | NO | YES | 2012-204 | *R. luteiventris* | Deschutes | OR | Trauma | predation or aggression |
| 2013 | YES | NO | 2013-086 | *P. crucifer* | New Castle | DE | Viral infection | Ranavirus |
| 2013 | YES | NO | 2013-086 | Unidentified | New Castle | DE | Viral infection | Ranavirus |
| 2013 | YES | NO | 2013-086 | *R. sylvatica* | New Castle | DE | Viral infection | Ranavirus |
| 2013 | YES | NO | 2013-086 | *B. americanus* | New Castle | DE | Viral infection | Ranavirus |
| 2013 | YES | NO | 2013-088 | *P. crucifer* | New Castle | DE | Viral infection | Ranavirus |
| 2013 | YES | NO | 2013-088 | *R. sylvatica* | New Castle | DE | Viral infection | Ranavirus |
| 2013 | YES | NO | 2013-109 | *R. sylvatica* | Ulster | NY | Viral infection | Ranavirus |
| 2013 | YES | NO | 2013-114 | *H. versicolor* | Kent | DE | Deformity | NA |
| 2013 | YES | NO | 2013-116 | *R. sylvatica* | Baltimore | MD | Viral infection | Ranavirus |
| 2013 | YES | NO | 2013-117 | *R. sylvatica* | Howard | MD | Viral infection | Ranavirus |
| 2013 | YES | NO | 2013-118 | *R. sylvatica* | Harford | MD | Viral infection | Ranavirus |
| 2013 | YES | NO | 2013-119 | *R. sylvatica* | Montgomery | MD | Viral infection | Ranavirus |
| 2013 | YES | NO | 2013-120 | *R. sylvatica* | Frederick | MD | Viral infection | Ranavirus |
| 2013 | YES | NO | 2013-121 | *S. holbrookii* | Talbot | MD | Viral infection | Ranavirus |
| 2013 | YES | NO | 2013-122 | *R. catesbeiana* | Howard | MD | Viral infection | Ranavirus |
| 2013 | YES | NO | 2013-122 | *B. americanus* | Howard | MD | Viral infection | Ranavirus |
| 2013 | YES | NO | 2013-123 | *R. sylvatica* | Washington | MD | Undetermined | NA |
| 2013 | YES | NO | 2013-134 | *R. draytonii* | Monterey | CA | Fungal infection | *B. dendrobatidis* |
| 2013 | YES | NO | 2013-142 | *R. sylvatica* | Morris | NJ | Viral infection | Ranavirus |
| 2013 | YES | NO | 2013-143 | *R. sylvatica* | Warren | NJ | Undetermined | NA |
| 2013 | NA | NA | 2013-168 | *R. catesbeiana* | Multnomah | OR | Undetermined | NA |
| 2013 | YES | NO | 2013-227 | *R. sylvatica* | Cumberland | ME | Viral infection | Ranavirus |
| 2014 | YES | NO | 2014-066 | *R. catesbeiana* | Sussex | DE | Viral infection | Ranavirus |
| 2014 | YES | NO | 2014-066 | Unidentified | Sussex | DE | Viral infection | Ranavirus |
| 2014 | YES | NO | 2014-066 | *R. sylvatica* | Sussex | DE | Viral infection | Ranavirus |
| 2014 | YES | NO | 2014-069 | *R. pipiens* | Trempealeau | WI | Fungal infection | *B. dendrobatidis* |
| 2014 | YES | NO | 2014-069 | *R. pipiens* | Trempealeau | WI | Emaciation | NA |
| 2014 | YES | NO | 2014-072 | *R. sylvatica* | Kent | DE | Viral infection | Ranavirus |
| 2014 | YES | NO | 2014-074 | *R. sphenocephala* | New Castle | DE | Viral infection | Ranavirus |
| 2014 | YES | NO | 2014-074 | Unidentified | New Castle | DE | Viral infection | Ranavirus |
| 2014 | YES | NO | 2014-074 | *R. sylvatica* | New Castle | DE | Viral infection | Ranavirus |
| 2014 | YES | NO | 2014-095 | *R. sylvatica* | York | ME | Viral infection | Ranavirus |
| 2014 | YES | NO | 2014-095 | *R. sylvatica* | York | ME | Undetermined | NA |
| 2014 | YES | NO | 2014-110 | *R. catesbeiana* | Vilas | WI | Viral infection | Ranavirus |
| 2014 | YES | NO | 2014-138 | *P. crucifer* | Sussex | NJ | Viral infection | Ranavirus |
| 2014 | YES | NO | 2014-138 | *R. sylvatica* | Sussex | NJ | Viral infection | Ranavirus |
| 2014 | YES | NO | 2014-164 | *R. sylvatica* | Warren | NJ | Viral infection | Ranavirus |
| 2014 | YES | NO | 2014-164 | *R. sylvatica* | Warren | PA | Viral infection | Ranavirus |
| 2014 | YES | NO | 2014-189 | *R. sevosa* | Harrison | MS | Parasitism | Perkinsea protozoa |
| 2014 | NO | YES | 2014-201 | *R. clamitans* | Medina | OH | Trauma | predation or aggression |
| 2014 | YES | NO | 2014-204 | *R. sylvatica* | New Castle | DE | Undetermined | NA |
| 2014 | YES | NO | 2014-204 | *B. americanus* | New Castle | DE | Undetermined | NA |
| 2014 | YES | NO | 2014-222 | *R. sylvatica* | Penobscot | ME | Viral infection | Ranavirus |
| 2014 | YES | NO | 2014-238 | *R. clamitans* | Baltimore | MD | Viral infection | Ranavirus |
| 2014 | YES | NO | 2014-238 | *P. crucifer* | Baltimore | MD | Viral infection | Ranavirus |
| 2014 | YES | NO | 2014-238 | *R. sylvatica* | Baltimore | MD | Viral infection | Ranavirus |
| 2014 | YES | NO | 2014-239 | *R. clamitans* | Baltimore | MD | Viral infection | Ranavirus |
| 2014 | YES | NO | 2014-239 | *P. crucifer* | Baltimore | MD | Viral infection | Ranavirus |
| 2014 | YES | NO | 2014-239 | *R. sylvatica* | Baltimore | MD | Viral infection | Ranavirus |
| 2014 | YES | NO | 2014-240 | *R. sylvatica* | Howard | MD | Viral infection | Ranavirus |
| 2014 | YES | NO | 2014-240 | *B. americanus* | Howard | MD | Viral infection | Ranavirus |
| 2014 | YES | NO | 2014-241 | *R. sylvatica* | Howard | MD | Undetermined | NA |
| 2014 | YES | NO | 2014-242 | *R. clamitans* | Albemarle | VA | Trauma | predation or aggression |
| 2015 | YES | NO | 2015-019 | *R. sphenocephala* | Okaloosa | FL | Fungal infection | *B. dendrobatidis* |
| 2015 | YES | NO | 2015-019 | *R. sphenocephala* | Okaloosa | FL | Parasitism | Perkinsea protozoa |
| 2015 | NO | YES | 2015-039 | *R. chiricahuensis* | Grant | NM | Emaciation | starvation |
| 2015 | YES | NO | 2015-048 | *R. grylio* | Wakulla | FL | Viral infection | Ranavirus |
| 2015 | YES | NO | 2015-048 | *R. sphenocephala* | Wakulla | FL | Viral infection | Ranavirus |
| 2015 | NO | YES | 2015-051 | *R. sylvatica* | Fulton | PA | Trauma | NA |
| 2015 | NO | YES | 2015-052 | *R. chiricahuensis* | Sierra | NM | Trauma | predation or aggression |
| 2015 | NO | YES | 2015-052 | *R. chiricahuensis* | Sierra | NM | Undetermined | NA |
| 2015 | YES | NO | 2015-110 | *R. sylvatica* | Fairfax | VA | Viral infection | Ranavirus |
| 2015 | NO | YES | 2015-126 | *R. clamitans* | Buncombe | NC | Fungal infection | *B. dendrobatidis* |
| 2015 | NO | YES | 2015-256 | *H. cinerea* | Kleberg | TX | Natural toxin | brevetoxin |
| 2015 | YES | NO | 2015-271 | *R. sylvatica* | Penobscot | ME | Parasitism | *Ichthyophonus sp.* |
| 2015 | YES | NO | 2015-271 | *R. sylvatica* | Penobscot | ME | Viral infection | Ranavirus |
| 2015 | NO | YES | 2015-277 | *R. pipiens* | Monroe | NY | Viral infection | Ranavirus |
| 2015 | NO | YES | 2015-325 | *R. pipiens* | Rice | MN | Fungal infection | *B. dendrobatidis* |
| 2015 | NO | YES | 2015-325 | Unidentified | Rice | MN | Fungal infection | *B. dendrobatidis* |

| **Table S3:** GenBank accession numbers for DNA sequences used in the phylogenetic analysis of Perkinsea, Novel Alveolate Group 1.  Sequences that were newly generated for this project have listed sample identifiers; sequences already deposited in GenBank from previous work are listed as NA (= not applicable). | | | |
| --- | --- | --- | --- |
| **GenBank Accession Number** | **Sample origin** | **Location** | **Sample Identifier** |
| KP122562 | Environmental | United Kingdom | NA |
| KP122557 | Environmental | United Kingdom | NA |
| KP122534 | Environmental | United Kingdom | NA |
| KP122546 | Environmental | United Kingdom | NA |
| KP122539 | Environmental | United Kingdom | NA |
| KP122563 | Environmental | United Kingdom | NA |
| KP122559 | Environmental | United Kingdom | NA |
| KP122529 | Environmental | United Kingdom | NA |
| KP122530 | Environmental | United Kingdom | NA |
| KP122537 | Environmental | United Kingdom | NA |
| KP122540 | Environmental | United Kingdom | NA |
| KP122536 | Environmental | United Kingdom | NA |
| KP122551 | Environmental | United Kingdom | NA |
| KP122528 | Environmental | United Kingdom | NA |
| KP122552 | Environmental | United Kingdom | NA |
| KP122555 | Environmental | United Kingdom | NA |
| KP122558 | Environmental | United Kingdom | NA |
| KP122544 | Environmental | United Kingdom | NA |
| KP122560 | Environmental | United Kingdom | NA |
| KP122538 | Environmental | United Kingdom | NA |
| KP122556 | Environmental | United Kingdom | NA |
| KP122561 | Environmental | United Kingdom | NA |
| KP122554 | Environmental | United Kingdom | NA |
| KP122553 | Environmental | United Kingdom | NA |
| KP122527 | Environmental | United Kingdom | NA |
| KP122535 | Environmental | United Kingdom | NA |
| KP122564 | Environmental | United Kingdom | NA |
| KP122533 | Environmental | United Kingdom | NA |
| KP122532 | Environmental | United Kingdom | NA |
| KP122542 | Environmental | United Kingdom | NA |
| KP122531 | Environmental | United Kingdom | NA |
| KP122545 | Environmental | French Guiana | NA |
| KP122548 | Environmental | French Guiana | NA |
| KP122547 | Environmental | French Guiana | NA |
| KP122549 | Environmental | French Guiana | NA |
| KP122550 | Environmental | French Guiana | NA |
| KP122543 | Environmental | French Guiana | NA |
| KP122565 | Environmental | French Guiana | NA |
| KP122541 | Environmental | French Guiana | NA |
| DQ244038 | Environmental | France | NA |
| EU162626 | Environmental | France | NA |
| EU162629 | Environmental | France | NA |
| AY919735 | Environmental | New York, USA | NA |
| KP122697 | Amphibian | Cameroon | NA |
| KP122709 | Amphibian | Cameroon | NA |
| KP122632 | Amphibian | Cameroon | NA |
| KP122706 | Amphibian | Cameroon | NA |
| KP122704 | Amphibian | Cameroon | NA |
| KP122715 | Amphibian | Cameroon | NA |
| KP122633 | Amphibian | Cameroon | NA |
| KP122634 | Amphibian | Cameroon | NA |
| KP122584 | Amphibian | Cameroon | NA |
| KP122618 | Amphibian | Cameroon | NA |
| KP122648 | Amphibian | Cameroon | NA |
| KP122626 | Amphibian | Cameroon | NA |
| KP122730 | Amphibian | Cameroon | NA |
| KP122638 | Amphibian | Cameroon | NA |
| KP122667 | Amphibian | Cameroon | NA |
| KP122635 | Amphibian | Cameroon | NA |
| KP122619 | Amphibian | Cameroon | NA |
| KP122700 | Amphibian | Cameroon | NA |
| KP122692 | Amphibian | Cameroon | NA |
| KP122605 | Amphibian | Cameroon | NA |
| KP122570 | Amphibian | Cameroon | NA |
| KP122599 | Amphibian | Cameroon | NA |
| KP122582 | Amphibian | Cameroon | NA |
| KP122642 | Amphibian | Cameroon | NA |
| KP122643 | Amphibian | Cameroon | NA |
| KP122629 | Amphibian | Cameroon | NA |
| KP122649 | Amphibian | Cameroon | NA |
| KP122575 | Amphibian | Cameroon | NA |
| KP122574 | Amphibian | Cameroon | NA |
| KP122650 | Amphibian | Cameroon | NA |
| KP122724 | Amphibian | Cameroon | NA |
| KP122726 | Amphibian | Cameroon | NA |
| KP122639 | Amphibian | Cameroon | NA |
| KP122573 | Amphibian | Cameroon | NA |
| KP122651 | Amphibian | Cameroon | NA |
| KP122646 | Amphibian | Cameroon | NA |
| KP122592 | Amphibian | Cameroon | NA |
| KP122711 | Amphibian | Cameroon | NA |
| KP122658 | Amphibian | Cameroon | NA |
| KP122659 | Amphibian | Cameroon | NA |
| KP122729 | Amphibian | Cameroon | NA |
| KP122572 | Amphibian | Cameroon | NA |
| KP122660 | Amphibian | Cameroon | NA |
| KP122661 | Amphibian | Cameroon | NA |
| KP122590 | Amphibian | Cameroon | NA |
| KP122622 | Amphibian | Cameroon | NA |
| KP122600 | Amphibian | Cameroon | NA |
| KP122662 | Amphibian | Cameroon | NA |
| KP122588 | Amphibian | Cameroon | NA |
| KP122663 | Amphibian | Cameroon | NA |
| KP122586 | Amphibian | Cameroon | NA |
| KP122628 | Amphibian | Cameroon | NA |
| KP122744 | Amphibian | Cameroon | NA |
| KP122743 | Amphibian | Cameroon | NA |
| KP122675 | Amphibian | Cameroon | NA |
| KP122674 | Amphibian | Cameroon | NA |
| KP122676 | Amphibian | Cameroon | NA |
| KP122693 | Amphibian | Cameroon | NA |
| KP122733 | Amphibian | Cameroon | NA |
| KP122677 | Amphibian | Cameroon | NA |
| KP122735 | Amphibian | Cameroon | NA |
| KP122678 | Amphibian | Cameroon | NA |
| KP122637 | Amphibian | Tanzania | NA |
| KP122654 | Amphibian | Tanzania | NA |
| KP122684 | Amphibian | Tanzania | NA |
| KP122656 | Amphibian | Tanzania | NA |
| KP122617 | Amphibian | Tanzania | NA |
| KP122601 | Amphibian | Tanzania | NA |
| KP122680 | Amphibian | Tanzania | NA |
| KP122623 | Amphibian | Tanzania | NA |
| KP122580 | Amphibian | Tanzania | NA |
| KP122721 | Amphibian | Tanzania | NA |
| KP122699 | Amphibian | Tanzania | NA |
| KP122719 | Amphibian | Tanzania | NA |
| KP122596 | Amphibian | Tanzania | NA |
| KP122686 | Amphibian | Tanzania | NA |
| KP122717 | Amphibian | Tanzania | NA |
| KP122640 | Amphibian | Tanzania | NA |
| KP122595 | Amphibian | Tanzania | NA |
| KP122723 | Amphibian | Tanzania | NA |
| KP122641 | Amphibian | Tanzania | NA |
| KP122594 | Amphibian | Tanzania | NA |
| KP122625 | Amphibian | Tanzania | NA |
| KP122666 | Amphibian | Tanzania | NA |
| KP122620 | Amphibian | Tanzania | NA |
| KP122687 | Amphibian | Tanzania | NA |
| KP122630 | Amphibian | Tanzania | NA |
| KP122657 | Amphibian | Tanzania | NA |
| KP122606 | Amphibian | Tanzania | NA |
| KP122645 | Amphibian | Tanzania | NA |
| KP122689 | Amphibian | Tanzania | NA |
| KP122688 | Amphibian | Tanzania | NA |
| KP122691 | Amphibian | Tanzania | NA |
| KP122597 | Amphibian | Tanzania | NA |
| KP122728 | Amphibian | Tanzania | NA |
| KP122631 | Amphibian | Tanzania | NA |
| KP122710 | Amphibian | Tanzania | NA |
| KP122621 | Amphibian | Tanzania | NA |
| KP122603 | Amphibian | Tanzania | NA |
| KP122602 | Amphibian | Tanzania | NA |
| KP122636 | Amphibian | Tanzania | NA |
| KP122644 | Amphibian | Tanzania | NA |
| KP122701 | Amphibian | Tanzania | NA |
| KP122670 | Amphibian | Tanzania | NA |
| KP122669 | Amphibian | Tanzania | NA |
| KP122668 | Amphibian | Tanzania | NA |
| KP122702 | Amphibian | Tanzania | NA |
| KP122653 | Amphibian | Tanzania | NA |
| KP122683 | Amphibian | Tanzania | NA |
| KP122679 | Amphibian | Tanzania | NA |
| KP122652 | Amphibian | Tanzania | NA |
| KP122665 | Amphibian | Tanzania | NA |
| KP122690 | Amphibian | Tanzania | NA |
| KP122614 | Amphibian | Tanzania | NA |
| KP122703 | Amphibian | Tanzania | NA |
| KP122698 | Amphibian | Tanzania | NA |
| KP122579 | Amphibian | Tanzania | NA |
| KP122607 | Amphibian | Tanzania | NA |
| KP122608 | Amphibian | Tanzania | NA |
| KP122609 | Amphibian | Tanzania | NA |
| KP122610 | Amphibian | Tanzania | NA |
| KP122611 | Amphibian | Tanzania | NA |
| KP122713 | Amphibian | Tanzania | NA |
| KP122694 | Amphibian | Tanzania | NA |
| KP122593 | Amphibian | Tanzania | NA |
| KP122695 | Amphibian | Tanzania | NA |
| KP122696 | Amphibian | Tanzania | NA |
| KP122672 | Amphibian | Tanzania | NA |
| KP122578 | Amphibian | Tanzania | NA |
| KP122671 | Amphibian | Tanzania | NA |
| KP122576 | Amphibian | Tanzania | NA |
| KP122577 | Amphibian | Tanzania | NA |
| KP122571 | Amphibian | Tanzania | NA |
| KP122712 | Amphibian | Tanzania | NA |
| KP122681 | Amphibian | Tanzania | NA |
| KP122598 | Amphibian | Tanzania | NA |
| KP122655 | Amphibian | Tanzania | NA |
| KP122581 | Amphibian | French Guiana | NA |
| KP122604 | Amphibian | French Guiana | NA |
| KP122612 | Amphibian | French Guiana | NA |
| KP122613 | Amphibian | French Guiana | NA |
| KP122725 | Amphibian | French Guiana | NA |
| KP122727 | Amphibian | French Guiana | NA |
| KP122747 | Amphibian | French Guiana | NA |
| KP122714 | Amphibian | French Guiana | NA |
| KP122647 | Amphibian | French Guiana | NA |
| KP122616 | Amphibian | French Guiana | NA |
| KP122746 | Amphibian | French Guiana | NA |
| KP122682 | Amphibian | French Guiana | NA |
| KP122731 | Amphibian | São Tomé | NA |
| KP122627 | Amphibian | São Tomé | NA |
| KP122734 | Amphibian | São Tomé | NA |
| KP122740 | Amphibian | São Tomé | NA |
| KP122738 | Amphibian | São Tomé | NA |
| KP122736 | Amphibian | São Tomé | NA |
| KP122673 | Amphibian | São Tomé | NA |
| KP122737 | Amphibian | São Tomé | NA |
| KP122732 | Amphibian | São Tomé | NA |
| KP122708 | Amphibian | São Tomé | NA |
| KP122707 | Amphibian | São Tomé | NA |
| KP122745 | Amphibian | São Tomé | NA |
| KP122741 | Amphibian | São Tomé | NA |
| KP122739 | Amphibian | São Tomé | NA |
| KP122742 | Amphibian | São Tomé | NA |
| KP122591 | Amphibian | United Kingdom | NA |
| KP122589 | Amphibian | United Kingdom | NA |
| KP122587 | Amphibian | United Kingdom | NA |
| KP122685 | Amphibian | United Kingdom | NA |
| KP122585 | Amphibian | United Kingdom | NA |
| KP122722 | Amphibian | United Kingdom | NA |
| KP122705 | Amphibian | United Kingdom | NA |
| KP122716 | Amphibian | United Kingdom | NA |
| KP122720 | Amphibian | United Kingdom | NA |
| KP122718 | Amphibian | United Kingdom | NA |
| KP122583 | Amphibian | United Kingdom | NA |
| KP122624 | Amphibian | United Kingdom | NA |
| EF675616 | Amphibian | Georgia, USA | NA |
| KY679230 | Amphibian | Maine, USA | 4824-341 clone #2 |
| KY679231 | Amphibian | Maine, USA | 4824-341 clone #4 |
| KY679232 | Amphibian | Maine, USA | 4824-341 clone #6 |
| KY679233 | Amphibian | Maine, USA | 4824-341 clone #7 |
| KY679234 | Amphibian | Maine, USA | 4824-341 clone #8 |
| KY679235 | Amphibian | Maine, USA | 4824-341 clone #9 |
| KY679236 | Amphibian | Maine, USA | 4824-341 clone #10 |
| KY679237 | Amphibian | Maine, USA | 4824-341 clone #11 |
| KY679238 | Amphibian | Louisiana, USA | 4957-311 clone #2 |
| KY679239 | Amphibian | Louisiana, USA | 4957-311 clone #3 |
| KY679240 | Amphibian | Louisiana, USA | 4957-311 clone #5 |
| KY679241 | Amphibian | Louisiana, USA | 4957-311 clone #6 |
| KY679242 | Amphibian | Louisiana, USA | 4957-311 clone #9 |
| KY679243 | Amphibian | Louisiana, USA | 4957-311 clone #10 |
| KY679244 | Amphibian | Louisiana, USA | 4957-311 clone #11 |
| KY679245 | Amphibian | Louisiana, USA | 4957-311 clone #12 |
| KY679246 | Amphibian | New Hampshire, USA | 16407-003 clone #4 |
| KY679247 | Amphibian | New Hampshire, USA | 16407-003 clone #7 |
| KY679248 | Amphibian | New Hampshire, USA | 16407-003 clone #18 |
| KY679249 | Amphibian | New Hampshire, USA | 16407-003 clone #20 |
| KY679250 | Amphibian | New Hampshire, USA | 16407-003 clone #32 |
| KY679251 | Amphibian | New Hampshire, USA | 16407-003 clone #33 |
| KY679252 | Amphibian | New Hampshire, USA | 16407-003 clone #34 |
| KY679253 | Amphibian | New Hampshire, USA | 16407-003 clone #36 |
| KY679254 | Amphibian | Virginia, USA | 17272-015 clone #1 |
| KY679255 | Amphibian | Virginia, USA | 17272-015 clone #2 |
| KY679256 | Amphibian | Virginia, USA | 17272-015 clone #3 |
| KY679257 | Amphibian | Virginia, USA | 17272-015 clone #5 |
| KY679258 | Amphibian | Virginia, USA | 17272-015 clone #6 |
| KY679259 | Amphibian | Virginia, USA | 17272-015 clone #7 |
| KY679260 | Amphibian | Virginia, USA | 17272-015 clone #9 |
| KY679261 | Amphibian | Virginia, USA | 17272-015 clone #10 |
| KY679262 | Amphibian | Mississippi, USA | 18587-005 clone #1 |
| KY679263 | Amphibian | Mississippi, USA | 18587-005 clone #2 |
| KY679264 | Amphibian | Mississippi, USA | 18587-005 clone #3 |
| KY679265 | Amphibian | Mississippi, USA | 18587-005 clone #4 |
| KY679266 | Amphibian | Mississippi, USA | 18587-005 clone #5 |
| KY679267 | Amphibian | Mississippi, USA | 18587-005 clone #6 |
| KY679268 | Amphibian | Mississippi, USA | 18587-005 clone #7 |
| KY679269 | Amphibian | Mississippi, USA | 18587-005 clone #8 |
| KY679270 | Amphibian | Maryland, USA | 18709-002 clone #14 |
| KY679271 | Amphibian | Maryland, USA | 18709-002 clone #15 |
| KY679272 | Amphibian | Maryland, USA | 18709-002 clone #17 |
| KY679273 | Amphibian | Maryland, USA | 18709-002 clone #19 |
| KY679274 | Amphibian | Maryland, USA | 18709-002 clone #20 |
| KY679275 | Amphibian | Maryland, USA | 18709-002 clone #29 |
| KY679276 | Amphibian | Maryland, USA | 18709-002 clone #30 |
| KY679277 | Amphibian | Maryland, USA | 18709-002 clone #31 |
| KY679278 | Amphibian | Maryland, USA | 18761-005 clone #1 |
| KY679279 | Amphibian | Maryland, USA | 18761-005 clone #2 |
| KY679280 | Amphibian | Maryland, USA | 18761-005 clone #3 |
| KY679281 | Amphibian | Maryland, USA | 18761-005 clone #4 |
| KY679282 | Amphibian | Maryland, USA | 18761-005 clone #6 |
| KY679283 | Amphibian | Maryland, USA | 18761-005 clone #7 |
| KY679284 | Amphibian | Maryland, USA | 18761-005 clone #8 |
| KY679285 | Amphibian | Maryland, USA | 18761-005 clone #9 |
| KY679286 | Amphibian | New York, USA | 22445-004 clone #1 |
| KY679287 | Amphibian | New York, USA | 22445-004 clone #3 |
| KY679288 | Amphibian | New York, USA | 22445-004 clone #4 |
| KY679289 | Amphibian | New York, USA | 22445-004 clone #7 |
| KY679290 | Amphibian | New York, USA | 22445-004 clone #8 |
| KY679291 | Amphibian | New York, USA | 22445-004 clone #9 |
| KY679292 | Amphibian | New York, USA | 22445-004 clone #12 |
| KY679293 | Amphibian | New York, USA | 22445-004 clone #13 |
| KY679294 | Amphibian | Mississippi, USA | 24988-002 clone #2B |
| KY679295 | Amphibian | Mississippi, USA | 24988-002 clone #9B |
| KY679296 | Amphibian | Mississippi, USA | 24988-002 clone #10B |
| KY679297 | Amphibian | Mississippi, USA | 24988-002 clone #13B |
| KY679298 | Amphibian | Mississippi, USA | 24988-002 clone #14B |
| KY679299 | Amphibian | Mississippi, USA | 24988-002 clone #24 |
| KY679300 | Amphibian | Mississippi, USA | 24988-002 clone #27 |
| KY679301 | Amphibian | Mississippi, USA | 24988-002 clone #30 |
| KY679209 | Amphibian | Maine, USA | 4824-341 |
| KY679210 | Amphibian | Maine, USA | 4824-463 |
| KY679211 | Amphibian | Louisiana, USA | 4957-311 |
| KY679212 | Amphibian | Florida, USA | 4864-003 |
| KY679213 | Amphibian | Maine, USA | 4960-291 |
| KY679214 | Amphibian | New Hampshire, USA | 16407-003 |
| KY679215 | Amphibian | Virginia, USA | 17272-015 |
| KY679216 | Amphibian | Mississippi, USA | 18587-005 |
| KY679217 | Amphibian | Mississippi, USA | 18612-013 |
| KY679218 | Amphibian | Maryland, USA | 18709-002 |
| KY679219 | Amphibian | Maryland, USA | 18761-005 |
| KY679220 | Amphibian | Alaska, USA | 19156-001 |
| KY679221 | Amphibian | Alaska, USA | 19215-002 |
| KY679222 | Amphibian | Florida, USA | 19369-002 |
| KY679223 | Amphibian | Florida, USA | 19384-009 |
| KY679224 | Amphibian | Georgia, USA | 19709-006 |
| KY679225 | Amphibian | New York, USA | 22445-004 |
| KY679226 | Amphibian | Mississippi, USA | 24988-002 |
| KY679227 | Amphibian | Maryland, USA | 44274-004 |
| KY679228 | Amphibian | Florida, USA | 44277-003 |
| KY679229 | Amphibian | Florida, USA | 44277-011 |
| AF126013 | *Perkinsus marinus* reference sequence | NA | NA |
| AF497479 | *Perkinsus marinus* reference sequence | NA | NA |
| AY486141 | *Perkinsus mediterraneus* reference sequence | NA | NA |
| AY486139 | *Perkinsus mediterraneus* reference sequence | NA | NA |
| AY487831 | *Perkinsus mediterraneus* reference sequence | NA | NA |
| AF102171 | *Perkinsus chesapeaki* reference sequence | NA | NA |
| AY305326 | *Perkinsus chesapeaki* reference sequence | NA | NA |
| AF133909 | *Parvilucifera infectans* reference sequence | NA | NA |

| **Table S4:** GenBank accession numbers for DNA sequences used in the phylogenetic analysis of anuran species examined in this study.  Sequences that were newly generated for this project have listed sample identifiers; sequences already deposited in GenBank from previous work are listed as "Reference Sequences." For samples examined in this study, the original identification of the anuran based on morphology is listed ("Original ID") along with the identification based on the phylogenetic analysis of the mitochondrial DNA that was sequenced ("Genetic ID"). | | | | |
| --- | --- | --- | --- | --- |
| **Identifier** | **Original ID** | **Genetic ID** | **GenBank Accession Number** | **Reference** |
| 16407-003 | *Rana catesbeiana* | *R. catesbeiana* | KY677760 | this study |
| 17272-015 | *Rana sylvatica* | *R. sylvatica* | KY677772 | this study |
| 18216-007 | *R. sylvatica* | *R. sylvatica* | KY677779 | this study |
| 18487-008 | *Rana sphenocephala* | *R. sphenocephala* | KY677780 | this study |
| 18587-005 | *Rana sevosa* | *R. sevosa* | KY677762 | this study |
| 18610-057 | *R. sphenocephala* | *R. sphenocephala* | KY677792 | this study |
| 18612-013 | *R. sphenocephala* | *R. sevosa* | KY677763 | this study |
| 18613-022 | *R. sphenocephala* | *R. sphenocephala* | KY677816 | this study |
| 18626-001 | *R. sphenocephala* | *R. sphenocephala* | KY677783 | this study |
| 18709-002 | *R. sphenocephala* | *R. sphenocephala* | KY677764 | this study |
| 18761-005 | *R. sphenocephala* | *R. sphenocephala* | KY677766 | this study |
| 19156-001 | *R. sylvatica* | *R. sylvatica* | KY677774 | this study |
| 19215-002 | *R. sylvatica* | *R. sylvatica* | KY677767 | this study |
| 19323-008 | *R. catesbeiana* | *R. catesbeiana* | KY677817 | this study |
| 19369-002 | *Rana capito* | *R.capito* | KY677768 | this study |
| 19384-009 | *R. sphenocephala* | *R. sphenocephala* | KY677775 | this study |
| 19384-014 | *R. sphenocephala* | *R. sphenocephala* | KY677781 | this study |
| 19423-006 | *R. sphenocephala* | *R. sphenocephala* | KY677818 | this study |
| 19709-006 | *Rana heckscheri* | *R.heckscheri* | KY677776 | this study |
| 22445-004 | *R. sphenocephala* | *R.kauffeldi* | KY677771 | this study |
| 24988-002 | *R. sevosa* | *R. sevosa* | KY677778 | this study |
| 44274-002 | *Acris crepitans* | *A. crepitans* | KY677787 | this study |
| 44274-004 | *R. sphenocephala* | *R. catesbeiana* | KY677788 | this study |
| 44274-005 | *R. sphenocephala* | *R.clamitans* | KY677789 | this study |
| 44277-003 | *R. sphenocephala* | *R. sphenocephala* | KY677777 | this study |
| 44614-019 | *R. catesbeiana* | *R. catesbeiana* | KY677815 | this study |
| 44617-004 | *R. catesbeiana* | *R. catesbeiana* | KY677782 | this study |
| 4756-014 | *Pseudacris regilla* | *P. regilla* | KY677793 | this study |
| 4766-038 | *R. sylvatica* | *R. sylvatica* | KY677794 | this study |
| 4766-047 | *R. sylvatica* | *R. sylvatica* | KY677785 | this study |
| 4772-007 | *Rana cascadae* | *R. cascadae* | KY677795 | this study |
| 4823-062 | *R. catesbeiana* | *R. catesbeiana* | KY677796 | this study |
| 4823-082 | *Rana clamitans* | *R.clamitans* | KY677797 | this study |
| 4824-069 | *R. sylvatica* | *R. sylvatica* | KY677790 | this study |
| 4824-077 | *R. sylvatica* | *R. sylvatica* | KY677798 | this study |
| 4824-303 | *R. sylvatica* | *R. sylvatica* | KY677799 | this study |
| 4824-339 | *R. sylvatica* | *R. sylvatica* | KY677800 | this study |
| 4824-341 | *R. sylvatica* | *R. sylvatica* | KY677773 | this study |
| 4824-375 | *R. catesbeiana* | *R. catesbeiana* | KY677801 | this study |
| 4824-454 | *R.clamitans* | *R.clamitans* | KY677802 | this study |
| 4824-463 | *R.clamitans* | *R.clamitans* | KY677765 | this study |
| 4864-003 | *R. sphenocephala* | *R. sphenocephala* | KY677761 | this study |
| 4865-021 | *Rana palustris* | *R. sylvatica* | KY677786 | this study |
| 4866-021 | *P. regilla* | *P. regilla* | KY677803 | this study |
| 4866-091 | *Xenopus laevis* | *X. laevis* | KY677804 | this study |
| 4893-034 | *R. aurora* | *R. aurora* | KY677805 | this study |
| 4893-057 | *R. aurora* | *R. aurora* | KY677806 | this study |
| 4893-060 | *P. regilla* | *P. regilla* | KY677807 | this study |
| 4893-102 | *R. catesbeiana* | *R. catesbeiana* | KY677808 | this study |
| 4893-270 | *P. regilla* | *P. regilla* | KY677809 | this study |
| 4912-159 | *R. sphenocephala* | *R. sphenocephala* | KY677810 | this study |
| 4957-186 | *R. sphenocephala* | *R. sphenocephala* | KY677784 | this study |
| 4957-311 | *R. sphenocephala* | *R. sphenocephala* | KY677769 | this study |
| 4960-244 | *R.clamitans* | *R.clamitans* | KY677791 | this study |
| 4960-291 | *R.clamitans* | *R.clamitans* | KY677770 | this study |
| 4963-070 | *R. pipiens* | *R. pipiens* | KY677811 | this study |
| 4963-077 | *R. pipiens* | *R. pipiens* | KY677812 | this study |
| 4963-082 | *R. pipiens* | *R.clamitans* | KY677813 | this study |
| 4963-091 | *R. pipiens* | *R. pipiens* | KY677814 | this study |
| Reference Sequence | NA | *Ascaphus truei* | AJ871087 | Gissi et al. (2006) |
| Reference Sequence | NA | *Scaphiopus couchii* | JX564894 | Zhang et al. (2013) |
| Reference Sequence | NA | *Spea bombifrons* | JX564896 | Zhang et al. (2013) |
| Reference Sequence | NA | *X. laevis* | HM991335 | Irisarri et al. (2011) |
| Reference Sequence | NA | *Bufo americanus* | FJ882827 | Van Bocxlaer et al. (2009) |
| Reference Sequence | NA | *Bufo nebulifer* | HQ290945 | Santos and Cannatella (2011) |
| Reference Sequence | NA | *Bufo quercicus* | DQ283153 | Frost et al. (2006) |
| Reference Sequence | NA | *Bufo terrestris* | FJ882829 | Van Bocxlaer et al. (2009) |
| Reference Sequence | NA | *A. crepitans* | AY843559 | Faivovich et al. (2005) |
| Reference Sequence | NA | *A. gryllus* | AY843560 | Faivovich et al. (2005) |
| Reference Sequence | NA | *Hyla avivoca* | AY843605 | Faivovich et al. (2005) |
| Reference Sequence | NA | *Hyla cinerea* | AY549327 | Faivovich et al. (2005) |
| Reference Sequence | NA | *Hyla versicolor* | AY843682 | Faivovich et al. (2005) |
| Reference Sequence | NA | *P. cadaverina* | AY843734 | Faivovich et al. (2005) |
| Reference Sequence | NA | *P. crucifer* | AY843735 | Faivovich et al. (2005) |
| Reference Sequence | NA | *P. maculata* | AY291082 | Moriarty and Cannatella (2004) |
| Reference Sequence | NA | *P. regilla* | AY843737 | Faivovich et al. (2005) |
| Reference Sequence | NA | *P. triseriata* | AY843738 | Faivovich et al. (2005) |
| Reference Sequence | NA | *R. areolata* | AY779229 | Hillis and Wilcox (2005) |
| Reference Sequence | NA | *R. aurora* | AY779196 | Hillis and Wilcox (2005) |
| Reference Sequence | NA | *R. blairi* | AY779237 | Hillis and Wilcox (2005) |
| Reference Sequence | NA | *R. boylii* | AY779192 | Hillis and Wilcox (2005) |
| Reference Sequence | NA | *R.capito* | AY779231 | Hillis and Wilcox (2005) |
| Reference Sequence | NA | *R.cascadae* | AY779197 | Hillis and Wilcox (2005) |
| Reference Sequence | NA | *R. catesbeiana* | AY779206 | Hillis and Wilcox (2005) |
| Reference Sequence | NA | *R.clamitans* | AY779204 | Hillis and Wilcox (2005) |
| Reference Sequence | NA | *R. draytonii* | KP013110 | Renshaw et al. (unpublished) |
| Reference Sequence | NA | *R. grylio* | AY779201 | Hillis and Wilcox (2005) |
| Reference Sequence | NA | *R. hecksheri* | AY779205 | Hillis and Wilcox (2005) |
| Reference Sequence | NA | *R. luteiventris* | AY779193 | Hillis and Wilcox (2005) |
| Reference Sequence | NA | *R. mucosa* | AY779195 | Hillis and Wilcox (2005) |
| Reference Sequence | NA | *R. okaloosae* | AY779203 | Hillis and Wilcox (2005) |
| Reference Sequence | NA | *R. palustris* | AY779228 | Hillis and Wilcox (2005) |
| Reference Sequence | NA | *R. pipiens* | AY779221 | Hillis and Wilcox (2005) |
| Reference Sequence | NA | *R. septentrionalis* | AY779200 | Hillis and Wilcox (2005) |
| Reference Sequence | NA | *R. sevosa* | AY779230 | Hillis and Wilcox (2005) |
| Reference Sequence | NA | *R. sierra* | KX269211 | Yuan et al. (2016) |
| Reference Sequence | NA | *R. sphenocephala* | AY779251 | Hillis and Wilcox (2005) |
| Reference Sequence | NA | *R. sylvatica* | AY779198 | Hillis and Wilcox (2005) |
| Reference Sequence | NA | *R. virgatipes* | AY779202 | Hillis and Wilcox (2005) |

**Supplementary figures:**

**Figure S1:**

**
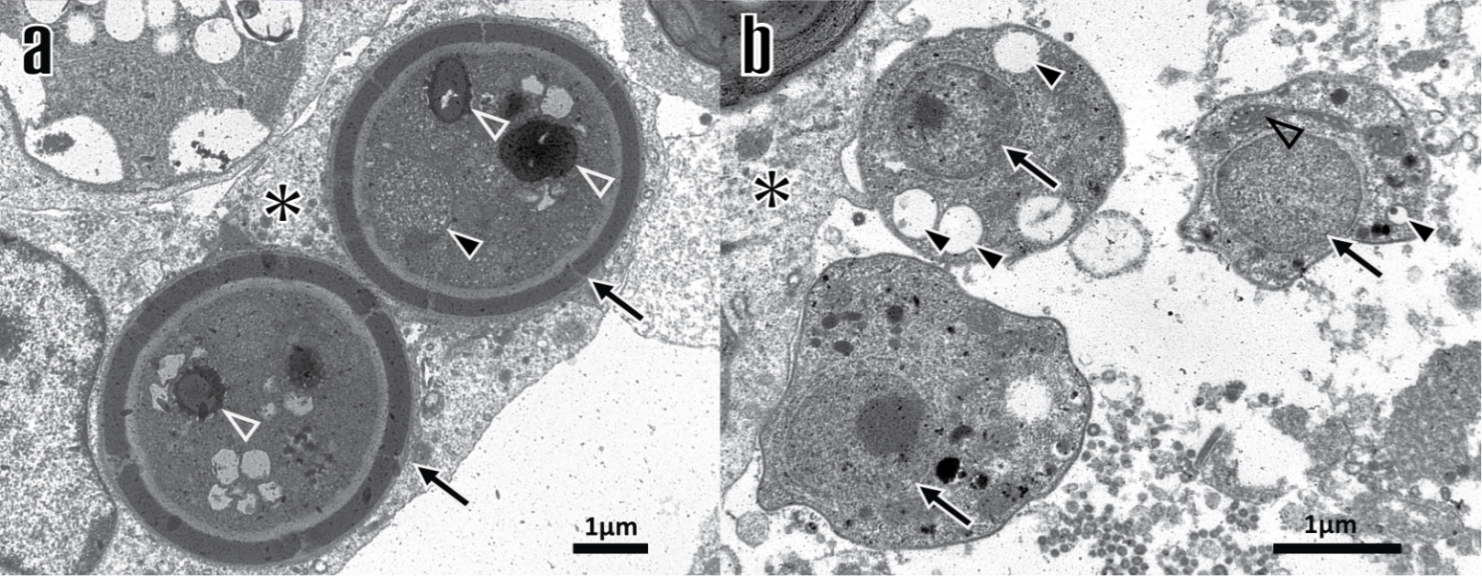
**

Figure S1: Transmission electron photomicrograph of liver from an SPI-positive American bullfrog tadpole (*Rana catesbeiana*; #16407-009). a) The cytoplasm of one hepatocyte (asterisk) is occupied by two Perkinsea-like protozoans compatible with spore-like stages with 0.6 to 0.8 µm thick trilaminar cell walls with electron-dense plates separated by transmural channels (arrows), single nucleus (arrowhead), and several lipid vacuoles up to 0.7µm in diameter (empty arrowheads). b) In the extracellular space, and attached to the cell membrane of one hepatocyte (asterisk) or free, there are three Perkinsea-like protozoans compatible with trophozoite-like stages with pleomorphic and amoeboid morphology, thin cell membranes, euchromatic nuclei (arrows) with prominent nucleoli, mitochondria (arrow), and several cytoplasmic vacuoles up to 0.4µm in diameter (arrowheads) with occasionally visible electro-dense vacuoplast.

**Figure S2:**

**
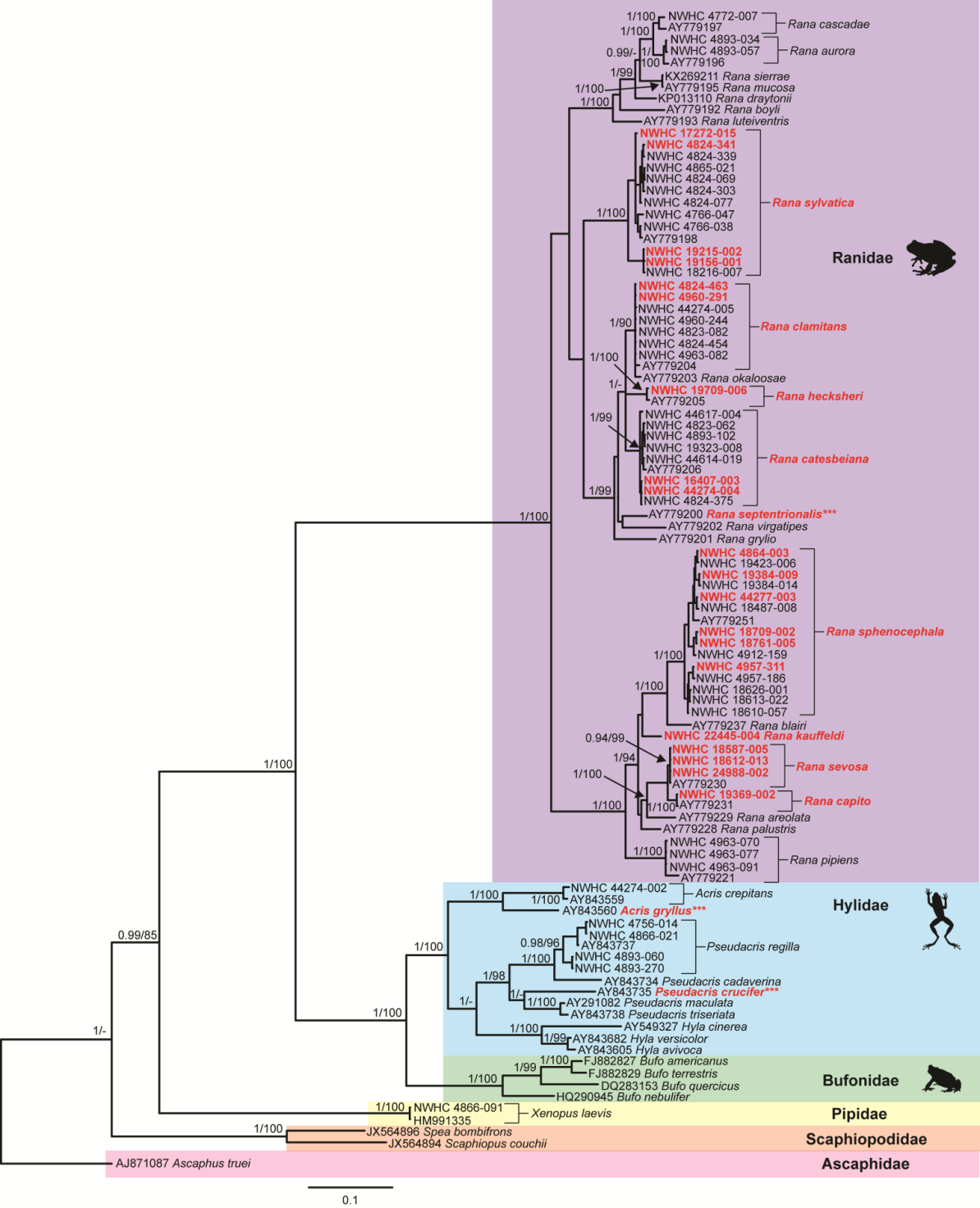
**

Figure S2: Phylogenetic tree demonstrating the currently-recognized host range of severe Perkinsea infections (SPI) in frogs. The final alignment used for the analysis included 1,718 characters of the approximately 2 kb portion of host mitochondrial genome. The tree from the Bayesian analysis is shown (the tree from the maximum likelihood analysis had a similar topology). Posterior probabilities (Bayesian)/bootstrap values (maximum likelihood) are shown at nodes when the support values were above 0.9 and 80, respectively. Sequences derived in this study are labelled as NWHC and have a unique identifier that is cross-listed in **Table S4**. Representative sequences from GenBank (denoted with a GenBank accession number) of frog species occurring in the known range of SPI were also included for reference purposes. Individual frogs with confirmed SPI by histopathology and PCR are colored in red. Species with confirmed infections are also colored in red. Species for which the infections were observed in histopathology but could not be tested with PCR are marked with asterisks (***). Note that SPI spans many taxa, including at least two families of Anura.
